# Circulating miRNAs and Multivariate Biomarker Signatures in Ovarian Cancer: A Systematic Review

**DOI:** 10.3390/ijms27198823

**Published:** 2026-10-02

**Authors:** Karen Ayache, Ana Vieira, Sofia Fragoso, Siobhan Brushett, Ioannis Gallos, Pushpa Patel, Jonathan Krell, Paula Cunnea, Dimitra Dionysiou, Fátima Vaz, Paula M. Mendes, Myrtani Pieri, Christos Papaneophytou

**Affiliations:** 1 Department of Life Sciences, School of Life and Health Sciences, University of Nicosia, Nicosia 2417, Cyprus; ayachkaren@gmail.com; 2 Non-Coding RNA Research Laboratory, School of Life and Health Sciences, University of Nicosia, Nicosia 2417, Cyprus; 3 Instituto Português de Oncologia de Lisboa Francisco Gentil, 1099-023 Lisbon, Portugal; acvieira@ipolisboa.min-saude.pt (A.V.); afragoso@ipolisboa.min-saude.pt (S.F.); fvaz@ipolisboa.min-saude.pt (F.V.); 4 Institute of Communication and Computer Systems, National Technical University of Athens, Zografos Campus, 15780 Athens, Greece; siobhan.brushett@iccs.gr (S.B.); ioannis.gallos@iccs.gr (I.G.); dimitra.dionysiou@iccs.gr (D.D.); 5 School of Chemical Engineering, University of Birmingham, Edgbaston, Birmingham B15 2TT, UK; p.patel.2@bham.ac.uk (P.P.); p.m.mendes@bham.ac.uk (P.M.M.); 6 Department of Surgery and Cancer, Imperial College London, London W12 0NN, UK; j.krell@imperial.ac.uk (J.K.); p.cunnea@imperial.ac.uk (P.C.)

**Keywords:** ovarian cancer, circulating microRNAs, liquid biopsy, biomarkers, early detection, prognosis

## Abstract

Ovarian cancer (OC) is the most lethal gynecological malignancy, largely because non-specific symptoms contribute to advanced-stage diagnosis and poor prognosis, underscoring the need for improved non-invasive biomarkers. Circulating microRNAs (c-miRNAs) have emerged as promising candidates for OC detection, risk stratification, prognosis, and treatment monitoring. This systematic review, prospectively registered in PROSPERO (CRD420251230898), evaluated the diagnostic and prognostic performance of c-miRNAs and miRNA-based multivariate signatures. MEDLINE/PubMed, Scopus, and CENTRAL were searched from inception to 30 May 2026. Eligible studies assessed c-miRNAs in serum, plasma, whole blood, or extracellular vesicle fractions from women with confirmed or suspected OC including studies using prediagnostic samples from women subsequently diagnosed with OC. Records were screened, data extracted, and risk of bias evaluated according to the type of result using QUADAS-2 for diagnostic accuracy, QUIPS for prognostic-factor associations, and PROBAST for multivariable prediction models. Of 697 records identified, 91 publications met the inclusion criteria. Most studies used case–control or observational designs and analysed serum or plasma samples. Single c-miRNAs generally showed moderate diagnostic accuracy, whereas multivariate miRNA panels and multimodal models incorporating CA-125 or HE4 generally reported higher AUC values, including in some early-stage cohorts. Frequently reported candidates included members of the miR-200 family, miR-21, miR-1246, miR-125b, miR-145, miR-205, and miR-130a. Prognostic studies associated c-miRNAs with survival, recurrence, treatment response, and chemoresistance, although findings were more heterogeneous than diagnostic findings. Risk-of-bias concerns were common, particularly for patient selection in diagnostic studies, confounding in prognostic-factor analyses, and statistical analysis in prediction-model studies. Overall, c-miRNAs represent promising non-invasive biomarkers for OC, particularly when integrated into multivariate or multimodal models. However, methodological heterogeneity, small cohort sizes, limited external validation, and inconsistent reporting currently restrict clinical translation. Future studies should prioritize standardized pre-analytical and analytical workflows, adequately powered prospective cohorts, and independent multicenter validation.

## 1. Introduction

Ovarian cancer (OC) is one of the most lethal gynecological malignancies, with approximately 42% of patients surviving five years after diagnosis, largely because most cases are detected at an advanced stage [1]. Circulating CA-125 and human epididymis protein 4 (HE4) are established serum biomarkers for OC; however, their sensitivity is limited in early-stage disease, with CA-125 being elevated in only approximately 50% of patients with stage I OC [1]. Moreover, their diagnostic performance varies across populations, stages, assay thresholds, and comparator groups [2,3,4,5,6]. These limitations have prompted the search for novel, non-invasive biomarkers that may improve early detection and risk stratification.

Circulating microRNAs (c-miRNAs) are small non-coding RNAs, generally approximately 20–25 nucleotides long, that are detectable in blood and other biofluids. Their stability in circulation and disease-associated expression profiles have prompted their investigation as minimally invasive biomarkers for OC [1,7,8,9]. Dysregulated circulating miRNAs reported in OC include members of the miR-200 family, miR-21, miR-1246, and miR-150-5p [9,10,11]. However, the direction and statistical significance of these associations have not always been reproduced across cohorts or analytical approaches. For example, an EV-based study identified preliminary differences in miR-21-5p and miR-150-5p, but these findings were not confirmed during technical replication and validation [7]. Circulating miRNA profiles have also been associated with adverse clinicopathological features and prognosis [9,12]. In one case–control study, higher total plasma cf-miRNA concentrations were associated with distant metastasis and poorer overall survival and remained independently associated with overall survival in multivariable analysis [9]. Individual circulating miRNAs have also been associated with tumour characteristics [11], while other extracellular miRNA profiles have been associated with progression-free and overall survival [12].

Multivariable circulating-miRNA panels have demonstrated promising diagnostic performance, and some studies have reported greater accuracy for miRNA panels than for individual miRNAs [8,13]. Nevertheless, performance estimates vary substantially among cohorts and study designs. Kumar et al. reported an AUC of 0.952, with 90.5% sensitivity and 100% specificity, for a three-miRNA serum panel distinguishing patients with stage I–II epithelial ovarian cancer from healthy controls [13]. In contrast, the seven-miRNA panel developed by Gahlawat et al. achieved an AUC of 0.81 for early-stage ovarian cancer, which increased to 0.93 when the panel was combined with CA-125 [9]. Yokoi et al. reported an AUC of 1.00, 99% sensitivity, and 100% specificity for a ten-miRNA model in an independent validation cohort; the model classified 95.1% of stage I cases as positive, although a separate stage I-specific AUC was not reported [8]. In another independent validation set, a two-miRNA classifier achieved an AUC of 0.789 for stage I–II disease, whereas integration of the miRNAs with CA-125 and HE4 increased the AUC to 1.00, with 88.9% sensitivity and 100% specificity [2]. The OCaMIR signature achieved stage I AUCs of 0.90 and 0.86 in two retrospective serum cohorts and 0.81 in a prospective serum cohort; its AUC for all-stage disease in the prospective cohort was 0.92 [14]. Although these findings demonstrate the potential of multianalyte miRNA-based models, their clinical applicability requires confirmation through larger, independently conducted prospective studies.

Despite these promising findings, the clinical translation of circulating miRNA biomarkers remains limited. Previous systematic evidence has identified small study populations, inconsistent normalization strategies, differences in patient selection and control groups, and substantial methodological heterogeneity as major barriers to reproducibility and cross-study comparison [1]. Standardization of sample collection, extracellular-vesicle isolation, miRNA quantification, normalization, and analytical reporting is therefore required, particularly for extracellular-vesicle-based biomarkers [1,15]. Previous systematic reviews and meta-analyses have primarily examined the diagnostic performance of circulating miRNAs [1,16] or focused specifically on exosomal miRNAs [17]. Consequently, an updated synthesis encompassing diagnostic, prognostic, treatment-response, longitudinal-monitoring, and multivariable biomarker studies is warranted. Conducted within the framework of the DISARM project, a Horizon Europe co-funded initiative focused on improving ovarian cancer risk assessment and early detection, this systematic review evaluates the diagnostic and prognostic performance of circulating miRNAs and multivariable biomarker signatures. It also examines their integration with established protein biomarkers, including CA-125 and HE4, and critically assesses the methodological and validation requirements necessary for their translation into clinical practice.

## 2. Results

### 2.1. Study Selection Results

**Figure 1:**
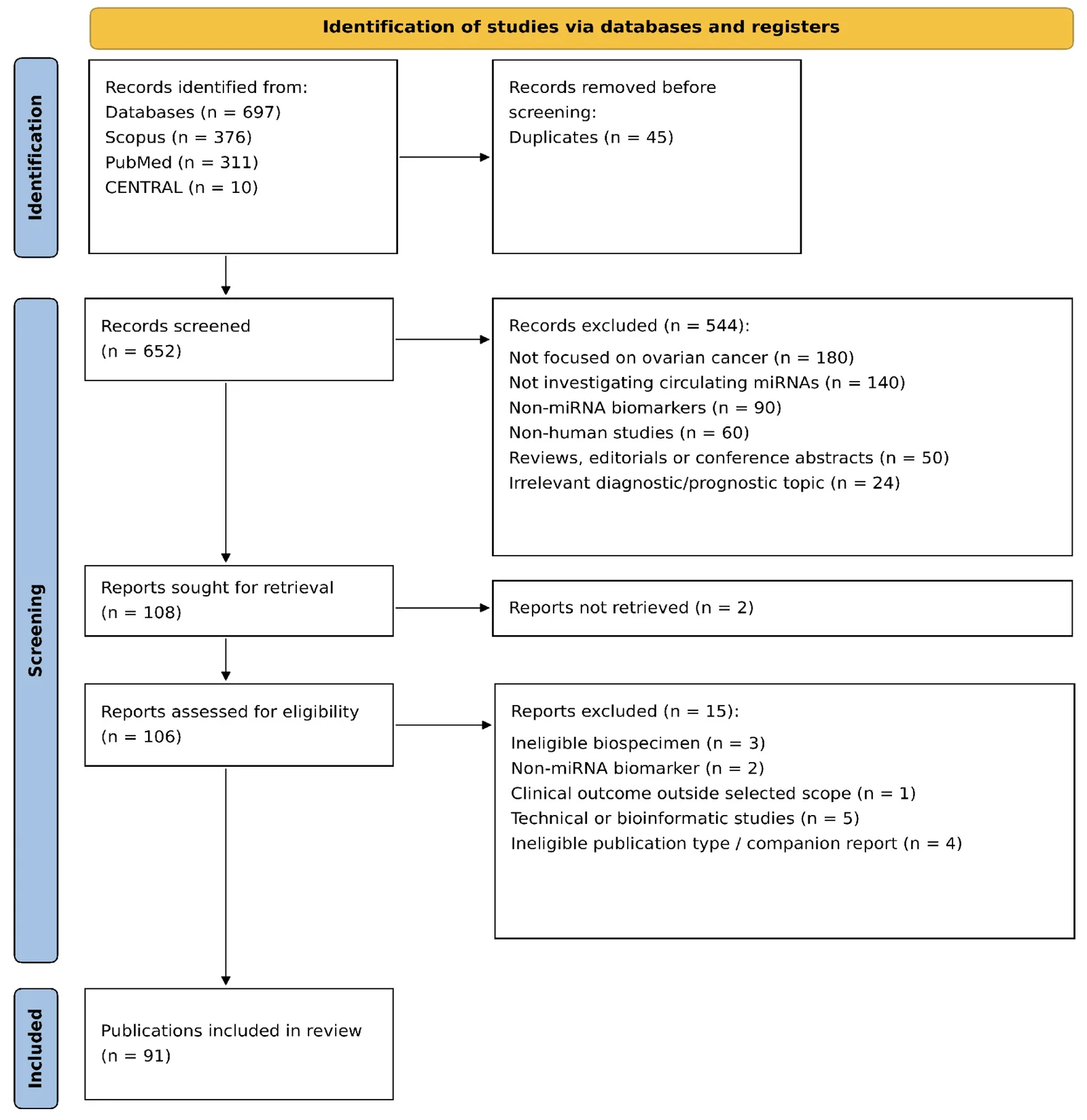
PRISMA flow diagram of study selection process. The diagram outlines the identification, screening, eligibility assessment, and inclusion of studies evaluating c-miRNAs as diagnostic and/or prognostic biomarkers in OC. A total of 697 records were retrieved from database searches, with 45 duplicates removed prior to screening. After title and abstract screening (*n* = 652), 544 records were excluded. Of 108 reports sought, two were not retrieved; 106 underwent full-text assessment and 15 were excluded. A final total of 91 publications were included in the systematic review.

The literature search identified a total of 697 records, including 376 from Scopus, 311 from MEDLINE/PubMed, and 10 from CENTRAL. After removing 45 duplicate records, 652 reports were screened for eligibility based on their titles and abstracts. Of these, 544 records were excluded. The primary reasons for exclusion at this stage were: studies not focused on OC (*n* = 180), studies not investigating c-miRNAs (*n* = 140), studies evaluating non-miRNA biomarkers (*n* = 90), non-human studies (*n* = 60), review articles/editorials/conference abstracts (*n* = 50), and irrelevant topics (*n* = 24). A total of 108 reports were sought for retrieval. Two reports could not be obtained, leaving 106 reports for full-text assessment. Of these, 15 reports were excluded: ineligible publication type or companion report (*n* = 4), ineligible biospecimen (*n* = 3), non-miRNA biomarker (*n* = 2), technical or bioinformatic study (*n* = 5), and clinical outcome outside the selected scope (*n* = 1). A referenced list of all reports excluded following full-text assessment, together with the primary reason for exclusion, is provided in Supplementary Table S6, which separately identifies the two reports not retrieved. Ultimately, 91 publications met all eligibility criteria and were included in the systematic review. The complete study selection process is summarized in the PRISMA flow diagram (Figure 1). Reassessment during revision added 18 eligible publications among the reports originally sought [18,19,20,21,22,23,24,25,26,27,28,29,30,31,32,33,34,35].

### 2.2. Study Characteristics

**Figure 2:**
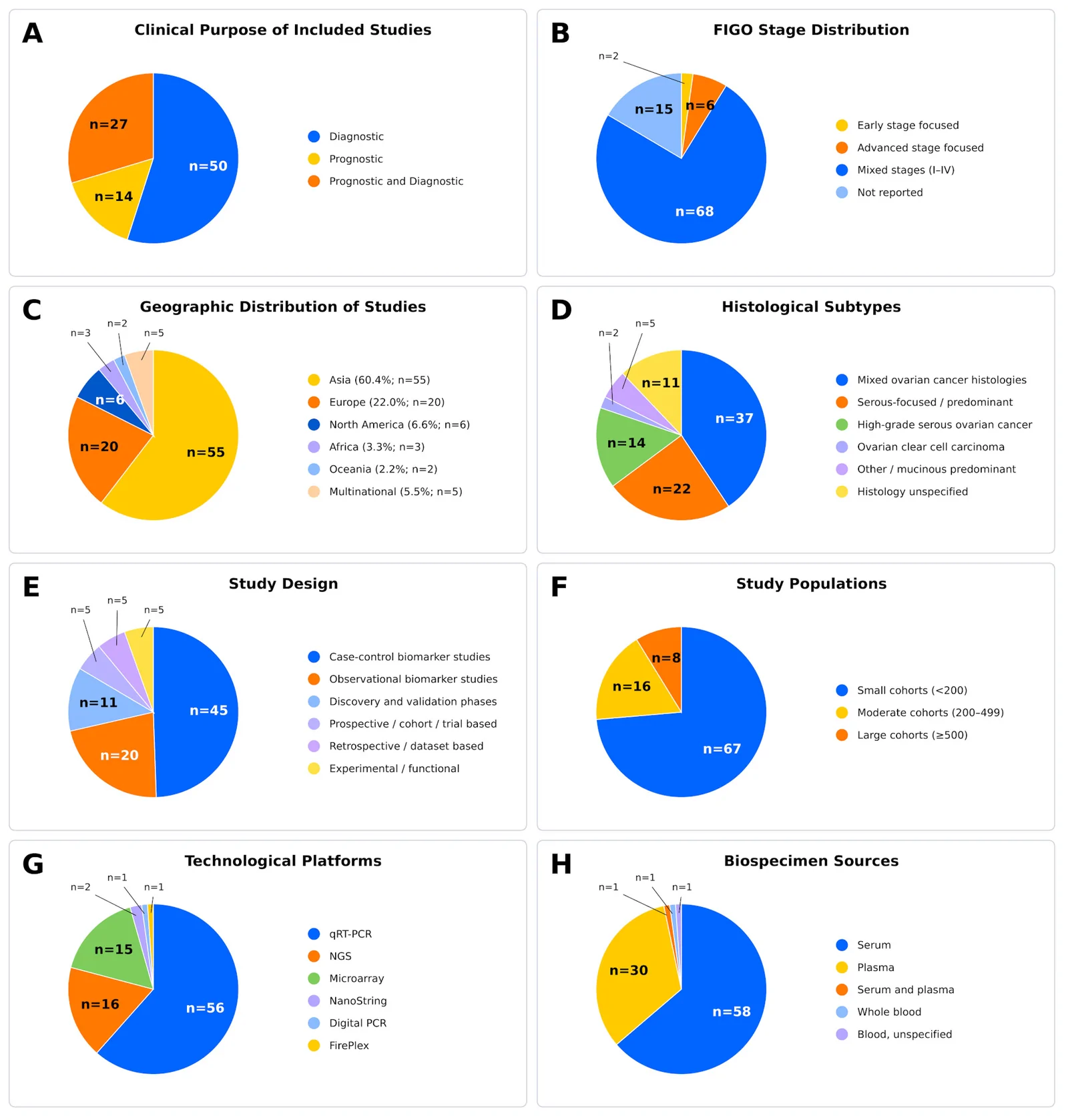
Clinical and methodological characteristics of the 91 publications included in the systematic review. (**A**) Clinical purpose of the included studies, classified as diagnostic, prognostic, or combined diagnostic-and-prognostic investigations. (**B**) Distribution of studies by FIGO stage within the study populations. (**C**) Geographic distribution of the included studies. (**D**) Histological subtypes investigated, including mixed-histology OC cohorts, serous-predominant disease, high-grade serous OC, ovarian clear cell carcinoma, and other or unspecified histologies. (**E**) Study designs used across the included literature. (**F**) Distribution of studies according to cohort or sample size. (**G**) Main technological platforms used for circulating miRNA profiling and validation. (**H**) Biospecimen sources used for miRNA analysis, classified as serum, plasma, both serum and plasma, whole blood, or blood with an unspecified fraction. Numbers shown within each chart represent the number of studies in each category.

A total of 91 publications were included in this systematic review. Their principal clinical and methodological characteristics are summarized in Figure 2A–H. Cohort-overlap assessment identified probable reuse among Kumar [13,36], Wang [37,38], Meng [25,39,40], and Zuberi [19,41,42] publications; the Zuberi reports had matching cohort sizes and stage distributions from the same institution. Ruan [27] reanalysed Fukagawa’s serum dataset [43]. Overlap remained unconfirmed between Márton [44] and Penyige [45], among the Istanbul reports [21,33,35,46], and between the Timofeeva reports [31,32]. These reports were retained for their distinct biomarkers, biospecimen fractions, or analyses, but were not treated as independent validation. Counts represent publications, not necessarily independent cohorts.

As shown in Figure 2A, 50 publications primarily investigated diagnostic applications of circulating miRNAs, 14 focused on prognostic or predictive outcomes, and 27 evaluated both diagnostic and prognostic applications. This distribution demonstrates that most of the available literature has concentrated on the potential use of circulating miRNAs for OC detection, although a substantial proportion of studies also examined their association with survival, recurrence, treatment response, or other clinical outcomes.

The distribution of disease stages is presented in Figure 2B. Most studies enrolled patients across mixed FIGO stages I–IV (*n* = 68). Six publications focused mainly on advanced-stage disease, whereas only two (*n* = 2) studies specifically concentrated on early-stage OC. Disease stage was not reported or was insufficiently described in fifteen publications. This predominance of mixed-stage and advanced-stage cohorts should be considered when interpreting reported diagnostic performance, particularly the applicability of candidate biomarkers to early disease detection.

The geographical distribution of the included studies is illustrated in Figure 2C. Most investigations were conducted in Asia (*n* = 55), followed by Europe (*n* = 20), North America (*n* = 6), Africa (*n* = 3), and Oceania (*n* = 2). Five studies involved multinational or multicontinental cohorts. The strong geographical concentration in Asia may limit the generalizability of some biomarker signatures, particularly where external validation across ethnically and geographically diverse populations was not performed.

As shown in Figure 2D, mixed-histology OC cohorts represented the largest category (*n* = 37). Twenty-two publications included serous-focused or serous-predominant cohorts, while 14 specifically investigated high-grade serous OC. Two studies focused exclusively on ovarian clear cell carcinoma, five investigated other specified histologies, and eleven did not clearly report histological subtype. The predominance of mixed epithelial and serous cohorts reflects the clinical frequency of epithelial and high-grade serous OCs but provides comparatively limited evidence for rarer histological subtypes.

The methodological designs of the included studies are summarized in Figure 2E. Case–control biomarker studies were the most common design (*n* = 45), followed by observational biomarker studies (*n* = 20). Eleven publications used distinct discovery and validation phases, five were prospective cohort or trial-based biomarker investigations, five were retrospective biomarker or dataset-based studies, and five were experimental or functional biomarker studies. The predominance of case–control designs may contribute to inflated estimates of diagnostic performance because patients with established OC are often compared with clearly defined healthy controls rather than clinically representative populations presenting with adnexal masses. Günel and Suryawanshi were classified as case–control studies because independence of discovery and confirmation samples was unclear or absent [21,22].

Study population sizes varied considerably, as shown in Figure 2F. Sixty-seven publications included fewer than 200 participants or samples, sixteen publications included between 200 and 499 participants, and eight involved at least 500 participants, samples, or multicentre validation datasets. The large proportion of relatively small studies indicates that many reported biomarker findings remain exploratory and require confirmation in adequately powered prospective cohorts.

The primary analytical platforms are summarized in Figure 2G: qRT-PCR (56 publications), NGS (16), hybridization microarray (15), NanoString (2), digital PCR (1), and FirePlex (1). PCR-based screening arrays were classified as qRT-PCR. The classification of each study, including whether it was a discovery or validation study, is shown in Supplementary Table S3.

The clinical blood matrices are summarized in Figure 2H: serum (58 publications), plasma (30), both serum and plasma (1), whole blood (1), and blood with an unspecified fraction (1). These mutually exclusive categories describe the clinical blood specimen; ancillary non-blood experiments do not create additional categories. Gumusoglu-Acar et al. [46] was coded as plasma because samples were collected in EDTA tubes, although the source inconsistently also uses “serum”. The same EDTA-based classification was applied to Günel et al. [35].

### 2.3. Diagnostic Performance of c-miRNAs in OC

The diagnostic performance of c-miRNAs in OC was evaluated across multiple studies using predominantly case–control and multi-cohort designs. The results in Table 1 show varying but generally moderate-to-good diagnostic accuracy.

Overall, single miRNA biomarkers demonstrated moderate diagnostic performance, with AUC values typically ranging from approximately 0.70 to 0.85. For example, miR-193a-5p and miR-205 showed modest discrimination between OC and controls, with reported AUCs of 0.708 and 0.715, respectively [3,47], while members of the miR-200 family and miR-1246 achieved comparatively higher accuracy with AUC values up to 0.89 for miR-1246 [48]. Serum miR-1246 demonstrated an AUC of 0.89, with 87% sensitivity and 77% specificity, for differentiating patients with high-grade serous ovarian carcinoma from healthy controls and was evaluated using separate training and validation cohorts [48]. These estimates should not be compared directly because the studies differed in histological composition, specimen type, comparator selection, analytical methods, and validation design. Although some individual c-miRNAs demonstrated promising diagnostic performance, many studies used modest sample sizes and lacked independent external validation. Larger prospective studies employing standardized methods and clinically relevant comparator groups are therefore required before individual c-miRNAs can be implemented in clinical practice.

Multivariable miRNA signatures often demonstrated higher apparent diagnostic performance than individual miRNAs, and several studies reported improved AUCs when multiple miRNAs were combined within the same cohort [13,36,49,50]. Across the included studies, several multi-miRNA signatures achieved AUCs above 0.85 [14,49,51,52,53], while selected miRNA or multianalyte models reported AUCs of approximately 0.95 or higher [4,8,11,13,36,50]. However, these estimates were obtained using different patient populations, comparator groups, analytical platforms, and validation strategies and therefore should not be compared directly. Notably, the ten-miRNA model developed by Yokoi et al. achieved an AUC of 1.00, with 99% sensitivity and 100% specificity, in an independent validation cohort [8]. Other computationally derived models also demonstrated high apparent diagnostic accuracy [52,54,55]. The modelling approaches employed across the included studies included logistic regression [13,36,50,55], random forests, artificial neural networks, and XGBoost [55], as well as neural-network classifiers [52] and relative-expression-ordering classifiers [54]. Collectively, these findings indicate that multivariable miRNA signatures are promising diagnostic tools; however, differences in model development, internal and external validation, and comparator selection limit direct comparisons and preclude firm conclusions regarding their relative clinical performance.

Combining circulating miRNAs with established protein biomarkers, particularly CA-125 and HE4, frequently improved diagnostic performance within individual studies. In an independent validation cohort, a model combining miR-320b and miR-141-3p with CA-125 and HE4 achieved an AUC of 0.989, compared with 0.723 for the miRNA-only classifier; among patients with stage I–II disease, the corresponding AUCs were 1.00 and 0.789, respectively [2]. Similarly, a model combining miR-193a-5p, CA-125, and HE4 achieved an AUC of 0.996 for distinguishing epithelial ovarian cancer from healthy controls, although it was evaluated in a single cohort without independent validation [3]. A multimodal model incorporating serum miRNAs, protein biomarkers, and clinical variables achieved an AUC of 0.95 (95% CI: 0.90–0.98), with 92% sensitivity at 80% specificity, in an external validation cohort [4].

A subset of studies specifically evaluated early-stage OC detection, which remains a major unmet clinical need. Reported sensitivities for early-stage disease varied across studies and models. A 14-miRNA neural-network model achieved 73% sensitivity and 91% specificity in its validation subset [51], whereas a miRNA–protein model achieved 88.9% sensitivity and 100% specificity [2]. The multimodal model developed by Webber et al. achieved 80% sensitivity for early-stage cancers in external validation [4]. A combination of plasma exosomal miR-223 and CA-125 distinguished stage I–II epithelial ovarian cancer from healthy controls with an AUC of 0.944 (95% CI: 0.899–0.990), 97.8% sensitivity, and 88.5% specificity [56]. These estimates were derived using different comparator groups and validation approaches and therefore are not directly comparable.

Extracellular-vesicle-derived miRNA signatures also demonstrated promising diagnostic performance. The eight-miRNA OCEM signature achieved an AUC of 0.9762 in the plasma training set and 0.9375 in the internal plasma validation set for distinguishing OC from benign disease [53]. Although miR-1246 and miR-1290 were elevated in early-stage samples, the study did not report a separate early-stage-specific AUC for the OCEM signature [53].

Substantial heterogeneity was evident across the included studies in study design, cohort size, specimen source, comparator composition, analytical platform, and validation strategy. Some exploratory studies did not report complete diagnostic performance measures. For example, Penyige et al. identified differentially expressed plasma miRNAs but did not report ROC AUC, sensitivity, or specificity [45]. In contrast, the Bayesian analysis by Wiczling et al. reported limited cross-validated discrimination, with AUCs of 0.65 for three selected individual miRNAs (reported credible intervals spanning 0.62–0.67), an AUC of 0.65 for a three-miRNA combination, and an AUC of 0.72 when a larger miRNA subset was evaluated [57].

Overall, the descriptive findings suggest that multi-miRNA and multimodal models frequently achieved higher apparent diagnostic accuracy than individual miRNAs, including in some early-stage analyses. However, no quantitative meta-analysis or formal cross-study comparison of single-miRNA and multivariable models was performed, and the heterogeneity of the study populations, comparator groups, analytical methods, and validation designs limits conclusions regarding their relative performance.

**Table 1:** Summary of diagnostic-focused c-miRNA studies included in the systematic review.

Author	miRNA/Signature	Control Composition/Comparator	Diagnostic Performance (AUC, Sensitivity, Specificity and 95% CI)	Diagnostic Interpretation of the Findings	Study Population and Sample Size	Early-Stage Cases (FIGO I–II, *n*)	Validation Type and Validation Sample Size
Ali et al. (2022) [58]	miR-204	OC vs. benign ovarian tumours vs. healthy controls; compared with CA-125/CA19-9	Sensitivity ≈80%; specificity ≈86.7%; combination improved accuracy	miR-204 was dysregulated in OC and showed good diagnostic value; combining it with CA-125/CA19-9 improved accuracy.	150 participants: 120 patients with benign, early, or late ovarian disease and 30 healthy controls; patient-subgroup sizes not reported.	Not reported numerically; early disease defined as FIGO I–II.	None reported; validation sample size not reported.
Choi et al. (2020) [49]	miR-200 family: miR-200a/b/c, miR-429, miR-141	OC vs. healthy controls	AUC = 0.904 American model; 0.901 Chinese model	miR-200 family was elevated in OC; multi-miRNA models showed high accuracy with ethnic differences.	Source cohort: 230 participants (112 controls, 118 ovarian cancer); primary human analysis *N* = 214 (84 American, 130 Chinese). The unexplained 16-participant difference is retained. Separate laying-hen samples: 11 normal, 15 cancer.	Not reported.	Internal 7:3 training/test split, with American and Chinese models reported separately; numerical split sizes not reported.
Cirillo et al. (2021) [2]	miR-320b + miR-141-3p; also with CA-125/HE4	OC vs. healthy controls; early-/late-stage OC vs. controls	miRNA model AUC = 0.723; early-stage AUC = 0.789; miRNA + protein AUC = 0.989; early-stage combined AUC = 1.000, sensitivity 88.9%, specificity 100%	The 2-miRNA model had moderate accuracy; adding CA-125/HE4 markedly improved OC detection, especially early-stage disease.	177 participants: discovery/training *N* = 115 (47 ovarian cancer, 68 controls) and clinical validation *N* = 62 (25 ovarian cancer, 37 controls).	30 cases: discovery/training *n* = 20; clinical validation *n* = 10.	Internal clinical holdout *N* = 62; discovery/training LOOCV *N* = 115; four external GEO series, sample sizes not reported.
Elias et al. (2017) [52]	Neural-network miRNA panel: miR-29a-3p, miR-92a-3p, miR-200c-3p, miR-320c, miR-335-5p, miR-450b-5p, miR-1307-5p	OC vs. benign disease, borderline tumours, healthy controls	Sequencing model AUC ≈0.90; NN AUC ≈0.93 vs. CA-125 AUC ≈0.74; qPCR validation AUC ≈0.85; PPV 91.3%, NPV 78.6%, specificity up to 100%	Serum multi-miRNA neural-network model outperformed CA-125 and remained robust across stages, subtypes, and platforms.	Sequencing/model cohort *N* = 179; overlapping qPCR model cohort *N* = 325; independent Polish cohort *N* = 51. Phase totals must not be summed as unique participants.	Initial *N* = 179 cohort: 57 by stage fields, 51 by histology rows, or 53 after assignment; qPCR *n* = 75; Polish *n* = 10.	Internal sequencing test *n* = 44; randomized 3:1 qPCR training/testing, test *n* not reported; independent external Polish validation *n* = 51.
Frisk et al. (2025) [7]	EV-miRNAs: discovery included miR-21-5p, miR-92a-3p, miR-140-5p/3p; validation tested 9 EV-miRNAs including miR-21-5p, miR-146b-5p, miR-106b-3p, miR-328-3p, miR-26a-5p, miR-150-5p, miR-342-3p, miR-485-3p, miR-29a-3p	OC vs. benign tumours; OC/pre-diagnostic samples vs. healthy controls	No AUC/sensitivity/specificity; discovery differences were not significant after correction and not confirmed in validation	EV-miRNAs did not reliably distinguish OC, benign tumours, controls, or pre-diagnostic samples; EV proteins CD9, CD151, and CD81 were more promising.	48 participants, 12 each with ovarian cancer, benign tumour, healthy control, or pre-diagnostic status; discovery used 24 participants, and same-cohort technical validation drew from all groups.	Clinical ovarian-cancer group: 0/12; pre-diagnostic group stage not reported.	Same-cohort RT-qPCR technical validation; no independent cohort or holdout. Exact successfully assayed n not reported; source cohort *N* = 48.
Gockley et al. (2022) [51]	14-miRNA panel, e.g., miR-23b-3p, miR-29a-3p, miR-92a-3p, miR-200a/c, miR-150, miR-1246, miR-1307; reduced 7-miRNA model	Benign adnexal masses + healthy controls	Validation AUC = 0.87; sensitivity 73%; specificity 91% for early-stage detection	14-miRNA neural-network model detected early-stage OC with good accuracy, including low tumour volume disease.	Human cohort *N* = 275: 75 ovarian cancer, 100 benign masses, 100 healthy controls. Separate experiments used 40 mice for training/testing and 30 mice for independent validation.	72/75 human cases were FIGO I–II; three additional cases were IIIA1–IIIA2.	Human internal 3:1:1 training/testing/validation split; validation number not reported. Independent animal validation *N* = 30 mice (15 controls, 15 xenografts).
Gumusoglu-Acar et al. (2023) [46]	Circulating miR-1909-5p, miR-885-5p and let-7d-3p; ancillary tissue miR-200c-3p.	Healthy individuals; simple ovarian cyst tissue controls	Circulating AUCs: miR-1909-5p, 1.00; miR-885-5p, 0.78; let-7d-3p, 0.70. Tissue miR-200c-3p AUC 0.83 is not a blood-based result.	Identified circulating and tissue miRNAs with diagnostic potential, but findings were limited by small sample size.	RT-qPCR cohort: 30 participants, 40 specimens (10 EOC patients with paired serum/tissue, 10 healthy serum controls, 10 benign-tissue controls). Overlapping microarray subset: 24 participants, 32 specimens.	3/10 EOC participants; the overlapping microarray subset contains the same three cases.	Same-cohort RT-qPCR assay validation: 30 participants contributing 40 specimens; no independent cohort.
Hamidi et al. (2021) [55]	10-miRNA signature: miR-5100, miR-6800-5p, miR-1233-5p, miR-4532, miR-4783-3p, miR-4787-3p, miR-1228-5p, miR-1290, miR-3184-5p, miR-320b	OC vs. non-cancer controls	AUC up to 100% using LR/RF/ANN/XGBoost; ≥95% in external validation	Machine-learning models using 10 circulating miRNAs achieved excellent OC classification.	Public serum datasets: GSE106817 *N* = 3079 (320 ovarian cancer, 2759 non-cancer) and GSE113486 *N* = 92 (40 ovarian cancer, 52 non-cancer); 3171 samples across datasets.	Not reported; stage data were unavailable in GSE106817.	Internal test *n* = 923 and training *n* = 2156, with five-fold training cross-validation; external independent validation *n* = 92.
Hong et al. (2022) [54]	13-miRPairs REO classifier; 20 miRNAs total; 17-miRPairs for OC vs. other cancers	Non-cancer serum samples; other cancer types	Sensitivity 97.5–98.33%; specificity 99.21–100%; PPV up to 100%, NPV up to 99.8%; AUC > 0.92 for multi-cancer discrimination	REO-based classifiers showed very high accuracy, early-stage performance, and resistance to batch/normalization effects.	GSE106817: training *n* = 2200 (200 ovarian cancer, 2000 non-cancer) and internal test *n* = 879 (120 ovarian cancer, 759 non-cancer). External GSE113486: *n* = 140 (40 ovarian cancer, 100 healthy controls).	GSE106817: stage I *n* = 81; stage II not reported, precluding a complete FIGO I–II count.	Internal holdout *n* = 879 (120 ovarian cancer, 759 non-cancer); external independent validation *n* = 140 (40 ovarian cancer, 100 healthy controls).
Kan et al. (2012) [59]	miR-200a, miR-200b, miR-200c ± miR-103 normalization	OC vs. healthy controls	AUC = 0.784 for miR-200b + miR-200c model	miR-200 family was elevated in SEOC; combined markers improved accuracy over single miRNAs.	56 participants/serum samples: 28 serous epithelial ovarian cancer and 28 healthy controls.	1/28 cancer cases (FIGO I *n* = 0; FIGO II *n* = 1).	Internal leave-one-out cross-validation of all 56 participants; no separate validation cohort.
Kandimalla et al. (2021) [14]	OCaMIR 8-miRNA signature: MIR182, MIR183, MIR202, MIR205, MIR508, MIR509-3, MIR513B, MIR513C	OC vs. healthy controls; specificity tested against other diseases/cancers; compared with CA-125	AUCs: tissue stage I 0.99; TCGA stage I 0.96; TCGA all stages 0.89; GSE65819 0.83; serum training 0.89; retrospective validation 0.82–0.86; prospective 0.92; stage I serum 0.81–0.90	OCaMIR showed strong reproducible diagnostic accuracy across retrospective and prospective cohorts and performed well for early-stage OC.	Tissue: discovery *n* = 31; validation *n* = 543 and *n* = 87. Serum: training *n* = 640; retrospective validation *n* = 85, *n* = 140, and *n* = 192; prospective validation *n* = 95. Total: 661 tissue and 1152 serum specimens.	Discovery tissue: stage I *n* = 22, stage II not reported; TCGA *n* = 37; serum training *n* = 111; Czech *n* = 27; West China *n* = 13.	External validation: tissue cohorts *n* = 543 and *n* = 87; retrospective serum cohorts *n* = 85, *n* = 140, and *n* = 192; prospective serum cohort *n* = 95.
Khan et al. (2023) [60]	miR-223 with TPD52, KLF9, PKCε	OC vs. healthy controls	AUC: miR-223 = 0.7915; TPD52 = 0.7769; PKCε = 0.7630; KLF9 = 0.5608	miR-223/TPD52 were upregulated; KLF9/PKCε downregulated; biomarkers showed moderate accuracy and correlated with stage/metastasis.	300 participants: 150 ovarian cancer patients and 150 healthy controls; blood samples were analysed.	54/150 ovarian-cancer participants.	No participant-level validation; sample size not applicable. Technical qPCR triplicates only.
Kumar et al. (2021) [13]	miR-205, miR-200c, miR-141 panel	EOC vs. healthy controls	Serum individual AUCs: advanced disease, 0.876/0.782/0.860; early disease, 0.881/0.789/0.867. Three-miRNA panel: 0.959 advanced and 0.952 early (Results section and Figure 7 of Kumar et al. [13]); abstract reports a conflicting early value of 0.981.	Three hypomethylated miRNAs were upregulated in EOC; the combined panel showed excellent early-stage diagnostic accuracy.	Tissue cohort: 115 specimens (85 EOC, 30 normal); serum cohort: 65 matched specimens (51 EOC, 14 normal); MeDIP-NGS subset: 8 tissue specimens (6 EOC, 2 normal).	Tissue *n* = 41; serum *n* = 21; MeDIP-NGS subset *n* = 2. Phase-specific counts should not be combined.	No separate participant cohort, split-sample validation, or resampling; validation sample size not reported.
Kumar et al. (2022) [36]	miR-34a, let-7f, miR-31 panel	Healthy controls; normal ovarian tissue and serum	Tissue AUCs: 92.1–97.0; serum AUCs: 85.6–92.0; combined panel AUC: tissue 98.6–98.9, serum 95.5–96.9	Downregulated hypermethylation-linked miRNAs strongly discriminated against EOC from controls, including early-stage disease.	Tissue cohort: 115 specimens (85 EOC, 30 normal); serum cohort: 65 matched specimens (50 EOC, 15 normal); unique participants across phases not determinable. MeDIP-NGS subset: 8 tissue specimens.	Tissue *n* = 41; serum *n* = 20; MeDIP-NGS subset *n* = 2. Phase-specific counts should not be combined.	Internal specimen-type validation using 65 serum specimens (50 EOC, 15 normal), including 20 FIGO I–II; independence from the tissue cohort not demonstrated.
Langhe et al. (2015) [61]	let-7i-5p, miR-122-5p, miR-152, miR-25-3p	OC vs. benign ovarian disease	Significant differential expression; fold change ≈ 1.7–2.2; *p* < 0.05; no ROC/AUC/sensitivity/specificity	4-miRNA serum panel was downregulated in OC and distinguished malignant from benign disease but lacked ROC validation.	50 participants: pilot/training *n* = 10 (5 ovarian carcinoma, 5 benign cystadenoma) and validation *n* = 40 (20 ovarian carcinoma, 20 benign cystadenoma).	6/25 malignant cases (FIGO IC *n* = 3, IIB *n* = 1, IIC *n* = 2).	Internal holdout validation *n* = 40 (20 ovarian carcinoma, 20 benign cystadenoma); not an external independent cohort.
Liang et al. (2020) [5]	miR-183 + HE4 + CA-125	OC vs. benign disease vs. healthy controls	miR-183 AUC = 0.77; combined model AUC = 0.951, sensitivity 0.97, specificity 0.85	Combining miR-183 with HE4/CA-125 improved diagnostic accuracy over single markers.	Training *N* = 201 (101 ovarian cancer, 50 benign disease, 50 healthy controls); separately reported validation *N* = 201 (98 ovarian cancer, 50 benign disease, 53 healthy controls); 402 observations overall.	Training *n* = 40 (stage I *n* = 18; II *n* = 22); validation *n* = 39 (stage I *n* = 19; II *n* = 20).	Separate same-setting clinical validation *N* = 201 (98 ovarian cancer, 50 benign disease, 53 healthy controls); allocation procedure not reported.
Liu et al. (2021) [62]	Plasma exosomal miR-4732-5p	OC vs. healthy controls	Validation AUC 0.889; sensitivity 85.7%, specificity 82.4%; 95% CI not reported in the text.	Single exosomal miR-4732-5p discriminated OC from healthy controls after sequencing-based discovery; no multimiRNA diagnostic panel was evaluated.	55 participants: discovery *n* = 10 (6 EOC, 4 healthy controls) and validation *n* = 45 (28 EOC, 17 healthy controls).	6/34 EOC participants: discovery 0/6; validation 6/28.	Separate additional validation dataset *n* = 45 (28 EOC, 17 healthy controls); external or independent recruitment not reported.
Márton et al. (2019) [44]	EMT-miRNAs: miR-200 family, miR-34 family, miR-203a	Healthy controls; non-malignant ovarian masses; CA-125/HE4 comparison	Best: miR-200c AUC = 0.861; others: miR-200a 0.845, miR-200b 0.822, miR-141 0.825, miR-203a 0.831, miR-34b 0.832; sensitivity up to 85.7%, specificity up to 90%	EMT-associated miRNAs, especially miR-200c, showed good accuracy and may complement CA-125/HE4.	100 participants: 28 malignant ovarian tumours, 12 non-malignant ovarian masses, and 60 healthy controls.	Not unambiguously reported because stage labels are absent from the PDF table.	None reported; no separate validation cohort, holdout, resampling, or external validation.
Niemira et al. (2023) [11]	miR-1246 ^1^, and miR-150-5p ↓ ^1^	HGSOC vs. healthy controls	Serum miR-1246/miR-150-5p model: NanoString training AUC ≈0.98, test 1.00; refitted qPCR test 0.946; refitted GEO test 0.895 (95% CI 0.848–0.941).	Promising case–control discrimination. qPCR and public-data analyses refitted the model; they do not establish external validation of an unchanged model.	NanoString *N* = 70, with conflicting case/control allocations; Table 1 training *n* = 49 and testing *n* = 21. RT-qPCR *N* = 88 (42 HGSOC, 46 controls), split 62/26. External GEO *N* = 640 (320 ovarian cancer, 320 controls).	NanoString *n* = 4; RT-qPCR *n* = 4; external GEO *n* = 110/320 cases, not the external test-subset count.	Internal tests: NanoString *n* = 21; qPCR *n* = 26. Public GEO data *n* = 640 were split 70:30 and a new model was trained. The three analyses are not locked-model external validations.
Oliveira et al. (2019) [10]	cf-miRNA panel: miR-200c-3p, miR-221-3p, miR-21-5p, miR-484, miR-195-5p, miR-451a	OC vs. benign pelvic masses	miR-200c-3p AUC = 0.78; miR-221-3p AUC ≈0.65; 2-miRNA panel AUC ≈0.82; with CA-125 AUC ≈0.96, sensitivity ≈80%, specificity ≈95%	Plasma miRNAs distinguished OC from benign pelvic masses; CA-125 combination substantially improved performance.	380 enrolled: discovery *n* = 190 (95 ovarian cancer, 95 benign mass) and validation *n* = 190 (95 ovarian cancer, 95 benign mass). Across cohorts, 14 cancer and 13 benign participants were excluded; analysed cohort-specific denominators not reported.	Enrolled: discovery *n* = 13; validation *n* = 0. Post-exclusion counts not reported.	Independent validation cohort: 190 enrolled (95 ovarian cancer, 95 benign mass); analysed n after exclusions not reported.
Paliwal et al. (2020) [63]	miR-21 ↑ ^1^, miR-22 ↓ ^1^	OC vs. healthy controls	No AUC reported	miR-21 was upregulated 7.85-fold and miR-22 downregulated 2.1-fold; both correlated with stage/subtype.	160 participants: 80 epithelial ovarian cancer and 80 healthy controls.	32/80 ovarian-cancer participants.	None reported; validation sample size not applicable.
Penyige et al. (2019) [45]	Plasma miRNA profile: 45 differentially expressed; 17 significant	OC vs. healthy controls	No ROC/AUC/sensitivity/specificity; exploratory profiling only	Differential plasma miRNAs, including early-stage-specific candidates, suggested biomarker potential but require validation.	24 participants: 18 ovarian cancer and 6 healthy controls.	6/18 ovarian-cancer participants (FIGO I *n* = 6; II *n* = 0).	No participant-level validation cohort; same-sample RT-qPCR analytical confirmation n not separately reported.
Ren et al. (2018) [3]	miR-193a-5p ± HE4 + CA-125	EOC vs. benign ovarian tumours and healthy controls	miR-193a-5p AUC = 0.708, sensitivity 66.7%, specificity 72.5%; HE4 AUC = 0.877; CA-125 AUC = 0.900; combined AUC = 0.996, sensitivity 93.3%, specificity 97.5%	miR-193a-5p was downregulated in EOC; combining with HE4/CA-125 produced excellent diagnostic accuracy.	115 participants: 45 primary EOC, 30 benign ovarian tumour, and 40 healthy controls.	12/45 EOC participants; III–IV *n* = 26 and stage unknown *n* = 7.	None reported; model development and ROC evaluation used the same sample set.
Ritter et al. (2020) [64]	Candidate miRNAs; main validated gynaecological candidates: serum miR-484 and urine miR-205-5p	Healthy controls; mainly EC+OC vs. controls	No ROC/AUC/sensitivity/specificity; serum miR-484 significant in EC+OC vs. controls, *p* < 0.001; urine miR-205-5p nonsignificant after correction	Mixed breast/gynaecological cancer study; OC-specific conclusions limited.	Discovery: 37 participants (27 patients, 10 controls), with serum and urine collected. Validation: 144 participants (82 cancer, 62 controls); overlapping matrix subsets comprised 91 serum and 75 urine samples.	Discovery: 0/4 confirmed ovarian cancers; validation: serum 5/15 and urine 2/10; unique participant total not reported.	Separate independent same-center RT-qPCR validation: 144 participants (82 cancer, 62 controls); 91 serum and 75 overlapping urine samples.
Stawiski et al. (2024) [65]	14-miRNA panel: miR-23b-3p, miR-29a-3p, miR-32-5p, miR-92a-3p, miR-150-5p, miR-200a-3p, miR-200c-3p, miR-203a-3p, miR-320c/d, miR-335-5p, miR-450b-5p, miR-1246, miR-1307-5p	Cases close to diagnosis <2 years vs. distant ≥2–7 years; no healthy controls	Median AUC = 0.771; average AUC ≈0.781, 95% CI 0.72–0.84	Signature detected temporal proximity to diagnosis; miR-200a-3p was most important, but true-control validation was lacking.	Analytic dataset: 80 ovarian-cancer participants, one serum sample each (40 collected <2 years and 40 collected 2–7 years before diagnosis). Source dataset: 80 participants, 106 samples; controls excluded.	Not reported; localized/regional categories cannot be converted to FIGO I–II.	Internal repeated 70:30 training/test holdout, five repetitions, with five-fold training cross-validation; exact split counts not reported for the 80-sample dataset.
Su et al. (2019) [6]	Exosomal miR-1307 and miR-375	Benign ovarian tumours and healthy controls; compared with CA-125/HE4	miR-1307 AUC = 0.671–0.694; miR-375 AUC = 0.788; CA-125 AUC = 0.905–0.906; HE4 AUC = 0.820; miR-1307+miR-375 AUC = 0.837; +CA-125 AUC ≈0.970–0.977	Exosomal miR-1307/miR-375 were upregulated; combination with CA-125/HE4 markedly improved accuracy.	Conflicting totals: Methods *N* = 150 (50 ovarian cancer, 50 benign tumour, 50 healthy); Table 2 *N* = 115 (50 serous ovarian cancer, 40 benign, 25 controls).	Table 2: *n* = 8 (FIGO I *n* = 4; II *n* = 4).	Internal staged screening and further cohort analysis; distinct independent validation not demonstrated. Separate phase sizes not reported, and Methods/Table 2 totals conflict.
Takamizawa et al. (2024) [66]	miR-146a-5p, miR-191-5p; screening included miR-484, miR-574-3p	Healthy controls and endometriosis patients	miR-146a-5p AUC = 0.762, sensitivity 79.3%, specificity 50%; miR-191-5p AUC = 0.830, sensitivity 89.7%, specificity 50%	Markers were upregulated in ovarian clear cell carcinoma vs. controls but did not clearly distinguish carcinoma from endometriosis.	64 participants: 29 ovarian clear-cell carcinoma, 17 endometriosis, 18 healthy controls. Array discovery used 20 samples (16 carcinoma, 4 healthy); 12 carcinoma samples were reanalysed.	21/29 carcinoma cases.	Internal staged same-population confirmation; no separate validation sample. qPCR used 64 participants; ROC contrasts used 29 carcinoma cases and 18 healthy controls.
Todeschini et al. (2017) [48]	miR-1246; also miR-595, miR-2278	Healthy controls	miR-1246 AUC = 0.89, sensitivity 87%, specificity 77%, accuracy 84%	miR-1246 was upregulated in HGSOC serum and validated across cohorts/platforms.	233 participants/serum samples: training *n* = 162 (110 HGSOC, 52 controls) and validation *n* = 71 (58 HGSOC, 13 controls). Array-profiling subset: 110 HGSOC and 19 control sera.	0/168 HGSOC cases; all were FIGO III–IV.	External independent validation *n* = 71 participants/serum specimens (58 HGSOC, 13 healthy controls).
Wang et al. (2019a) [37]	Serum miR-200c-3p, miR-346, miR-127-3p, miR-143-3p and miR-205-5p	OC vs. healthy controls	Five-miRNA serum panel: training AUC 0.783 (95% CI 0.674–0.892); validation AUC 0.745 (0.670–0.821).	Five-miRNA serum signature showed moderate discrimination between OC and healthy controls. Exosomal/tissue expression analyses were supporting analyses, not external validation.	Serum cohort: 226 participants (110 ovarian cancer, 116 controls); training *n* = 66 and testing/validation *n* = 160. Additional exosome and tissue confirmation subsets each contained 32 + 32 and 22 + 22 specimens, respectively.	Serum cases *n* = 37: training *n* = 15; testing/validation *n* = 22.	Internal serum holdout *n* = 160 (80 ovarian cancer, 80 controls); no external independent cohort.
Wang et al. (2019b) [38]	Plasma miR-205-5p, miR-145-5p, miR-10a-5p, miR-346 and miR-328-3p	OC versus healthy controls.	Five-miRNA plasma panel: training AUC 0.788 (95% CI 0.666–0.911); validation AUC 0.763 (0.682–0.844).	Moderate diagnostic discrimination. Tumour-grade association does not establish prognostic accuracy. No survival or treatment-outcome prediction was reported.	201 participants in training and validation: 101 OC and 100 healthy controls. Training: 32 OC/34 controls; validation: 69 OC/66 controls. Additional pooled discovery, tissue and exosome experiments are supporting analyses.	34/101 cases: training 13 (12 stage I, 1 stage II); validation 21 (14 stage I, 7 stage II).	Separate within-study training *n* = 66 and validation *n* = 135; no independent external clinical validation.
Wang et al. (2022) [53]	OCEM 8-miRNA EV signature: miR-1246, miR-1290, miR-483-5p, miR-429, miR-34b-3p, miR-34c-5p, miR-449a, miR-145-5p	Benign peritoneal fluid + benign plasma controls	Ascites AUC = 1.00; plasma AUC ≈0.98 training, 0.94 validation; external datasets AUC ≈0.90–0.96	Robust EV-based signature detected in ascites and blood; miR-1246/miR-1290 also promoted invasion/migration via RORα regulation.	In-house specimens: ascites EV, 10 malignant and 9 benign; plasma EV, 31 malignant and 24 benign. Participant overlap not reported. Public datasets: *N* = 3121, 140, 87, 20, and 10, reported separately.	Not reported.	Internal split-sample validation plus replication in five public datasets; exact validation splits not reported. Whole-dataset totals—3121, 140, 87, 20, and 10—are not validation-set sizes.
Webber et al. (2024) [4]	10-miRNA signature from 180 candidates, e.g., miR-320e, miR-195-5p, miR-103-3p, miR-200c-3p, miR-424-5p; combined with CA-125, HE4, ApoA1, B2M, TRF, prealbumin, FSH, metadata	Benign adnexal masses + healthy women; miRNA vs. protein/metadata vs. combined model	External validation AUC = 0.95, 95% CI 0.90–0.98; sensitivity 92%; specificity 80%; early-stage sensitivity 80%; late-stage 100%	Multimodal model improved diagnostic accuracy, especially for early-stage and non-serous OC, supporting adnexal mass triage.	678 participants: training *n* = 468 (191 cases, 277 controls), internal validation *n* = 100 (44 cases, 56 controls), and independent external validation *n* = 110 (51 cases, 59 controls).	59 cases overall: Aspira *n* = 39; external validation *n* = 20. Stage was unavailable for many Aspira participants.	Internal holdout *n* = 100 (44 cases, 56 controls); external independent validation *n* = 110 (51 cases, 59 controls); training cross-validation *n* = 468.
Wiczling et al. (2019) [57]	49 circulating miRNAs; key candidates miR-101-3p, miR-142-5p, miR-148a-3p	OC vs. healthy controls	Individual AUC ≈0.65; combined subset AUC ≈0.72; overall limited performance	Differential miRNAs had modest diagnostic performance; Bayesian model highlighted uncertainty and need for validation.	Two cohorts: discovery *n* = 59 (16 controls, 43 ovarian cancer) and second-stage *n* = 178 (119 controls, 59 ovarian cancer); 237 participants overall. Duplicate measurements were not additional participants.	Not reported.	Internal 10-fold cross-validation in the second-stage dataset *n* = 178; no fixed validation cohort, per-fold sizes, or external validation reported.
Yang et al. (2024) [56]	Plasma exosomal miR-223	EOC vs. benign tumours vs. healthy controls	miR-223 AUC = 0.812; CA-125 AUC = 0.933; miR-223+CA-125 AUC = 0.937, sensitivity ≈ 94.9%, specificity ≈ 81.5%; early-stage AUC = 0.94, sensitivity ≈ 97.8%, specificity ≈ 88.5%	Exosomal miR-223 was upregulated and correlated with stage/metastasis; combination with CA-125 improved early-stage detection.	170 participants: 78 epithelial ovarian cancer, 40 benign epithelial ovarian tumours, and 52 healthy controls.	39/78 ovarian-cancer participants (FIGO I *n* = 30; II *n* = 9).	None reported; no separate, split-sample, resampling, temporal, or external validation sample.
Yokoi et al. (2017) [50]	8-miRNA panel including miR-200a-3p, miR-766-3p, miR-26a-5p, miR-142-3p, let-7d-5p, miR-328-3p	OC vs. healthy; early OC vs. benign	AUC = 0.97 for OC vs. healthy; AUC = 0.90 for early OC vs. benign	Multi-miRNA model showed high accuracy for early detection and subtype classification.	Discovery *n* = 20; trial *n* = 45; development *n* = 269. Component cohorts total 250 tumour and 84 control samples, whereas Figure 1 reports 250 tumour and 83 prepared controls; discrepancy unresolved.	Discovery *n* = 6; development *n* = 65 (FIGO I *n* = 52; II *n* = 13); trial and overall totals not reported.	Internal repeated two-fold cross-validation of development *n* = 269; exact split sizes not reported. Trial *n* = 45 preceded final model development and was not final-model validation.
Yokoi et al. (2018) [8]	Integrated extracellular 10-miRNA OC detection panel	OC vs. benign tumours vs. healthy controls	AUC ≈ 0.99; sensitivity/specificity > 95%; strong stage I–II performance	10-miRNA serum signature showed excellent early-stage and overall OC detection, outperforming conventional biomarkers.	4046 participants/serum samples after exclusions: 333 ovarian carcinoma, 66 borderline tumour, 29 benign tumour, 2759 non-cancer controls, and 859 other cancers. Model 1 used *n* = 3187.	115 cases (FIGO I *n* = 82; II *n* = 33): discovery *n* = 54; validation *n* = 61.	Internal holdout *n* = 1648; primary ovarian-carcinoma versus non-cancer comparison *n* = 1540 (160 and 1380, respectively).
Yoshimura et al. (2018) [67]	miR-99a-5p	OC vs. benign vs. healthy	AUC = 0.88; sensitivity 0.85; specificity 0.75	miR-99a-5p was elevated in OC, decreased post-surgery, reflected tumours burden, and promoted invasion via exosomal signalling.	108 participants: 62 epithelial ovarian cancer, 26 benign ovarian tumour, and 20 healthy controls. Paired pre-/post-surgery subset: 26 participants with repeated specimens.	31/62 ovarian-cancer participants (FIGO I *n* = 26; II *n* = 5).	None reported; no separate model-validation sample. The 26-participant paired post-surgery subset was not validation.
Zhang & Hu (2022) [68]	4-miRNA panel: miR-5100, miR-1343-3p, miR-1290, miR-4787-3p	Non-cancer controls	Ovarian-cancer evaluation: AUC 0.967; sensitivity 68.2% at the transferred model threshold, with non-cancer specificity 99.3%. Lung-cancer AUC 0.999 is not an OC result.	A lung-trained four-miRNA model discriminated ovarian cancers in reused public serum data; prospective ovarian-cancer screening utility was not established.	Four public datasets: 7536 unique participants (3604 cancer, 3932 non-cancer). Deduplicated non-lung validation set: 3792 participants (2038 cancers, 1754 controls), including 333 ovarian-cancer cases reused from GSE106817.	115/333 cases (FIGO I *n* = 82; II *n* = 33), carried forward from reused GSE106817.	External independent-dataset evaluation: combined non-lung *n* = 3792; ovarian-specific contrast *n* = 2087 (333 ovarian cases, shared 1754-control pool).
Zhu et al. (2022) [47]	Exosomal miR-205 with CA-125, HE4, TCF21	OC vs. benign vs. healthy controls	miR-205 AUC = 0.715, sensitivity 66.7%, specificity 78.1%; combined miR-205+CA-125+HE4 AUC up to 0.951	miR-205 was upregulated in OC, higher in advanced stage and lymph node metastasis; combination with CA-125/HE4 improved diagnostic accuracy.	99 participants/plasma specimens: 36 ovarian cancer, 31 benign ovarian lesions, and 32 healthy controls.	Not reported.	None reported; no separate, split-sample, resampling, temporal, or external validation sample.
Günel et al. (2018) [21]	Serum hsa-miR-1273g-3p	Recurrent serous EOC versus healthy controls	AUC 0.70; threshold-specific sensitivity/specificity and AUC CI not reliably reported.	Detects existing recurrence; no prospective recurrence prediction or serial monitoring.	Microarray: 8 cases/8 controls; qPCR: 10/10. Overlap between phases unclear; do not sum as unique participants.	Not reported.	Microarray followed by qPCR confirmation; no established external validation.
Suryawanshi et al. (2013) [22]	Plasma three-miRNA LDA classifiers	Healthy controls or endometriosis; EAOC and serous OC evaluated separately.	EAOC vs. healthy: AUC 0.96, Se 86%, Sp 85%; EAOC vs. endometriosis: 0.92, 57%, 91%; serous OC vs. healthy: 0.90, 90%, 55%; serous OC vs. endometriosis: 0.88, 90%, 73%. CIs not reported.	Exploratory histotype-specific discrimination; no stage-specific accuracy or screening validation.	88 total: 14 EAOC, 21 serous OC, 33 endometriosis, 20 healthy controls.	12/35: 9 EAOC and 3 serous OC.	Discovery *n* = 20 is included in expanded *n* = 88; LOOCV, not independent validation.
Kumari et al. (2022) [24]	Serum miR-145; six candidate miRNAs assayed	OC expression comparisons with benign controls; ROC comparator composition is not unambiguously specified.	miR-145 AUC 0.790 (95% CI 0.653–0.927); source sensitivity 90%, specificity 73% (Table IV of Kumari et al. [24]).	No independent validation. AUC is reported for OC, but a pure cancer-versus-noncancer comparison cannot be confirmed; this estimate was excluded from the descriptive comparison of diagnostic AUCs.	40 women: 10 endometrioid OC, 10 endometrial cancer, 10 endometriosis and 10 benign gynecological controls.	Not reported.	No established independent clinical validation.
Meng et al. (2016) [25]	Cell-free miR-373 and miR-200a/b/c; three-miR-200 panel	OC versus noncancer comparators; see population column.	miR-200a/b/c AUCs 0.853/0.953/0.903; combined panel 0.959 (Figure 1.1 of Meng et al. [25])	Book-chapter report; possible reuse of the Hamburg cohort in [39,40]. No independent validation; comparator labels differ between figure and narrative.	60 OC, 20 benign ovarian tumours and 32 healthy controls.	8 stage I–II; 41 stage III–IV; 11 not reported.	No established independent clinical validation.
Xiong et al. (2021) [26]	Plasma exosomal miR-200b	OC versus noncancer comparators; see population column.	Increased expression in OC-derived plasma exosomes; no eligible AUC or sensitivity/specificity estimate.	Human plasma component only; cell-line, macrophage and animal experiments not counted as clinical prediction evidence.	6 epithelial OC and 3 healthy controls.	Not reported.	No established independent clinical validation.
Ruan et al. (2018) [27]	Serum miR-1181 and miR-4314; differential-expression profile	OC versus noncancer comparators; see population column.	Differential expression and same-dataset clustering; no diagnostic ROC AUC. Reported clustering accuracy is not independent validation.	Secondary analysis of the Fukagawa cohort [43]; do not count as independent recruitment. Group counts retained despite the source total discrepancy.	GSE79943: 29 OC and 3 healthy controls (32 listed samples); source also states 34.	7 stage I–II and 22 stage III–IV.	No established independent clinical validation.
Ji et al. (2014) [28]	Serum miR-22, miR-93, miR-106b and miR-451	OC versus noncancer comparators; see population column.	Differential expression; no diagnostic AUC.	Pooled sequencing discovery followed by qPCR; independence of confirmation samples not established. Treatment-history wording conflicts with the source clinical table.	31 OC, 23 benign ovarian tumours and 8 healthy controls.	7 stage I–II and 24 stage III–IV.	No established independent clinical validation.
Tian et al. (2025) [29]	miR-338-3p; CMD model combining it with three circRNAs	OC versus noncancer comparators; see population column.	miR-338-3p AUC 0.905 overall and 0.600 early-stage; CMD AUC 0.9789 overall and 1.000 early-stage (Figure 8B,C of Tian et al. [29]).	Same-cohort logistic models; no independent validation. CMD is a miRNA–circRNA model, not a multi-miRNA or miRNA–protein model.	22 OC and 28 healthy controls; early-stage ROC uses 5 early OC and 5 selected controls.	5 stage I–II; 9 stage III; 8 stage IV.	No established independent clinical validation.
Gumusoglu et al. (2021) [33]	Serum miRNA microarray profile; 75 miRNAs differing between OC and benign tumours	OC versus noncancer comparators; see population column.	Expression profiling; no diagnostic AUC or miRNA-specific clinical prognostic estimate.	No independent validation. Source Table 1 lists 9 serous and 1 endometrioid case, despite narrower histology wording in Methods. Possible cohort overlap with Istanbul reports.	36 recruited: 10 OC, 11 benign tumours, 15 healthy; quality-selected microarray subset: 8 per group (24).	3/10 OC stage I–II; 7 stage III.	No established independent clinical validation.
An et al. (2020) [34]	Serum miR-454	OC versus noncancer comparators; see population column.	Lower serum expression in OC; stage association does not establish prediction of a future outcome. No AUC.	Only serum results retained; ancillary tissue/cell experiments excluded from clinical biomarker counts.	13 OC and 6 healthy controls in the circulating-miRNA component.	7 stage I–II and 6 stage III–IV.	No established independent clinical validation.
Günel et al. (2019) [35]	Circulating hsa-let-7d-3p	OC versus noncancer comparators; see population column.	AUC 0.700 (95% CI 0.4595–0.9405); no independently validated threshold performance.	qPCR confirmation does not establish an independent cohort. Source calls the specimen serum but describes EDTA collection; coded plasma consistently with [46]. Possible Istanbul-cohort overlap.	Microarray component: 8 epithelial OC and 8 healthy controls; ancillary gene-expression samples not summed as independent participants.	Not reported.	No established independent clinical validation.

^1^ Arrows indicate increase ↑ and decrease ↓.

### 2.4. Prognostic Value of c-miRNAs

The prognostic relevance of c-miRNAs in OC was evaluated across a range of observational and cohort-based studies with varying clinical contexts and patient populations. As shown in Table 2, c-miRNAs were associated with key clinical outcomes, including OS, PFS, treatment response, and disease recurrence.

Several individual circulating miRNAs were associated with clinical outcomes. Higher serum miR-183 expression was associated with shorter OS and remained independently associated with OS in a multivariable Cox model [69]. Conversely, lower miR-148a expression was associated with shorter OS and remained an independent adverse prognostic factor after adjustment for clinicopathological variables [70]. Higher serum miR-21 and lower serum miR-145 levels were observed among patients with poorer chemotherapy response and among those who died within three years; however, this study evaluated three-year vital status using logistic regression rather than time-to-event survival analysis [71]. Higher serum miR-135a-3p expression was associated with longer PFS in an unadjusted Kaplan–Meier analysis, although an HR and 95% CI were not reported [43].

Evidence concerning multimiRNA prognostic signatures was more limited. Pal et al. reported that five circulating miRNAs—miR-27a, miR-182, miR-199a, miR-214, and miR-591—differed across chemotherapy-response groups and were associated with OS in unadjusted analyses [72]. However, the reported individual miRNA associations were not statistically significant in the multivariable Cox models; the adjusted covariates were not specified, and the signature was not evaluated in an independent validation cohort [72]. Therefore, the available evidence does not establish that multimiRNA signatures provide greater prognostic accuracy than individual circulating miRNAs.

Longitudinal measurements provided potentially useful information in some studies. Time-dependent plasma miR-200b levels were independently associated with PFS, whereas baseline miR-200b was not significantly associated with PFS, suggesting that serial measurements may provide information beyond a single baseline assessment [73]. In ovarian clear-cell carcinoma, serum miR-130a decreased following surgery and increased in all four patients who subsequently developed recurrence, sometimes before CA-125 increased [74]. However, the recurrence analysis was descriptive, involved only four recurrent cases, and did not report an HR, OR, or formal recurrence-prediction statistic.

Findings were not consistent across studies. In a first-line-treatment cohort, the kinetics of an 11-miRNA panel were heterogeneous and generally provided less effective treatment monitoring than CA-125, although selected miRNAs were associated with OS or PFS in univariable analyses [75]. Overall, c-miRNAs have potential as non-invasive prognostic and treatment-monitoring biomarkers in OC, particularly when evaluated longitudinally. Nevertheless, the evidence is limited by modest sample sizes, inconsistent outcome definitions, incomplete confounder adjustment, limited external validation, and the frequent reliance on univariable or exploratory analyses. Large, well-characterized prospective cohorts with prespecified models and independent validation are required before clinical utility can be established.

**Table 2:** Prognostic and treatment-response studies of circulating miRNAs in ovarian cancer.

Author	miRNAs	Outcome Measured and Effect Estimate	Statistical Analysis	Key Findings	Study Population and Sample Size	Early-Stage Cases (FIGO I–II, *n*)	Control/Comparison Group	Adjustment Variables	Validation Type and Validation Sample Size
Benson et al. (2015) [76]	78 circulating miRNAs; key: miR-193a-5p, miR-375, miR-339-3p, miR-340-5p, miR-532-3p, miR-133a-3p, miR-25-3p, miR-10a-5p, miR-616-5p, miR-148b-5p	Lower day-29 miR-148b-5p was associated with shorter PFS (log-rank *p* = 0.015); HR and 95% CI not reported.	Paired & two-tailed *t*-tests, Spearman correlation, Kaplan–Meier, log-rank	Chemotherapy altered circulating miRNA profiles; distinct responder/non-responder patterns observed. Lower post-treatment miR-148b-5p associated with worse PFS.	Recurrent platinum-resistant ovarian cancer: 14 of 17 phase II trial participants had paired plasma available (14 baseline and 14 day-29 samples; 28 samples total).	Not reported for the 14-patient miRNA cohort; all had recurrent platinum-resistant disease.	Within-patient baseline versus day 29; responders (*n* = 6) versus non-responders (*n* = 8). The paper describes response as progression after versus before six treatment cycles and also uses >6 versus <6 months in a table footnote.	None; survival analysis was an unadjusted Kaplan–Meier/log-rank comparison.	Exploratory single-cohort pilot study; no independent validation cohort.
Chao et al. (2014) [74]	miR-130a (main); also miR-138, miR-187, miR-202	miR-130a increased in all four recurrent cases and decreased in 3/6 non-recurrent cases; no HR/OR, 95% CI or formal recurrence-prediction *p*-value reported.	ΔCt quantification, Wilcoxon, Mann–Whitney U, fold-change analysis	miR-130a decreased after surgery and rose during recurrence, often earlier than CA-125, suggesting recurrence-monitoring potential.	Ovarian clear-cell carcinoma: 25 patients according to the Results and phase totals (discovery 4 paired sera; validation 11 paired sera; longitudinal application 10 patients).	19/25 (FIGO IC *n* = 17, IIB *n* = 1, IIC *n* = 1).	No healthy/benign control group; paired preoperative versus postoperative sera, then recurrent (*n* = 4) versus non-recurrent (*n* = 6) longitudinal patterns.	None; recurrence monitoring was descriptive and no regression-based prognostic model was reported.	Independent sample validation in 11 paired pre-/postoperative sera after discovery in 4 pairs; longitudinal application in 10 further patients. A TaqMan check used 12 pairs, but overlap with earlier phases is unclear.
Chen et al. (2016) [69]	miR-183	High serum miR-183 was associated with shorter OS. The author table labels the contrast ‘Low vs. High’ and reports univariable HR 7.541 (95% CI 5.885-11.329; *p* = 0.001) and multivariable HR 3.862 (95% CI 2.774–7.963; *p* = 0.003).	*t*-test, ANOVA, Chi-square, Kaplan–Meier, log-rank, Cox regression	miR-183 upregulated in advanced OC; associated with metastasis and poor OS; independent prognostic factor promoting proliferation and drug resistance.	Epithelial ovarian cancer: 75 patients (serum plus paired tumour/adjacent-normal tissue); prognostic groups were low serum miR-183 *n* = 36 and high *n* = 39.	34/75; FIGO III–IV *n* = 41.	For prognosis, low versus high serum miR-183 (mean cut-off); tissue expression used paired adjacent-normal tissue. No separate healthy serum control cohort.	Clinical stage, histology, lymph-node metastasis and serum miR-183 expression; age and histological grade were not entered/reported in the multivariable columns.	Single-cohort association/prognostic analysis; no independent validation cohort.
Fukagawa et al. (2017) [43]	miR-135a-3p; screened miR-630, miR-1207	Higher serum miR-135a-3p was associated with better PFS (log-rank *p* < 0.05); HR and 95% CI not reported.	Mann–Whitney U, Kaplan–Meier, log-rank, Pearson correlation	miR-135a-3p decreased in OC; higher levels linked with better PFS, enhanced chemosensitivity, and reduced tumour growth.	Discovery array: 12 ovarian-cancer patients (poor prognosis *n* = 6; favourable prognosis *n* = 6). Prognostic qRT-PCR assessment: 98 ovarian-cancer patients. Additional biomarker subsets comprised *n* = 30 and *n* = 17.	58/98 (FIGO I, 48; II, 10) in the prognostic qPCR cohort; source Supplementary Table S2. Additional groups: 20/30 and 13/17 early-stage cases.	Discovery: poor versus favourable prognosis (6 vs. 6). PFS: high versus low serum miR-135a-3p (median cut-off; group sizes not stated). Other expression analyses included benign gynaecological disease *n* = 7, benign ovarian tumour *n* = 24 and normal-ovary tissue RNA *n* = 5.	None; PFS analysis used unadjusted Kaplan–Meier/log-rank comparisons.	Separate 98-patient qRT-PCR prognostic assessment after a 12-patient array discovery; described as assessment rather than an external validation cohort.
Gong et al. (2016) [70]	miR-148a	Low versus high miR-148a: univariable OS HR 1.696 (95% CI 1.178–2.441; *p* = 0.005) and multivariable HR 1.699 (95% CI 1.175–2.456; *p* = 0.005); mean OS 31.6 versus 40.3 months (log-rank *p* = 0.002).	*t*-test, Chi-square, Kaplan–Meier, log-rank, Cox regression	miR-148a downregulated in OC; low levels associated with metastasis, advanced stage, and poor survival; independent prognostic biomarker.	Ovarian cancer *n* = 102 and age-matched healthy controls *n* = 70; prognostic groups were low miR-148a *n* = 47 and high *n* = 55.	60/102 had stage I–II disease, reported as TNM rather than explicitly as FIGO.	Expression comparison: 70 healthy volunteers. Prognosis: low miR-148a (*n* = 47) versus high miR-148a (*n* = 55).	Age, histopathological grade, TNM stage, lymph-node metastasis, tumour location, recurrence and miR-148a group.	Single clinical cohort; no independent prognostic validation. Cell-line experiments were functional studies, not clinical validation.
Halvorsen et al. (2017) [77]	miR-200b, miR-141, miR-1274A, miR-200c; screened 754 miRNAs	Validation-set multivariable PFS: miR-200b HR 0.798 (95% CI 0.68–0.94; *p* = 0.006); miR-1274A HR 0.846 (0.70–1.02; *p* = 0.085); miR-141 HR 0.914 (0.81–1.03; *p* = 0.153). High-grade serous miR-1274A HR 0.78 (0.62–0.99; *p* = 0.041). In low-miR-200c patients, bevacizumab versus standard treatment yielded median PFS 19.5 versus 14.5 months (*p* = 0.006); the paper reports adjusted HR 4.33 (1.96–9.58; *p* < 0.0001), but does not clearly state the HR reference direction.	SAM analysis, *t*-test, Kaplan–Meier, log-rank, multivariate Cox regression	miR-200b was a robust prognostic marker. Low miR-200b/miR-141/miR-1274A associated with better survival. miR-200c predicted bevacizumab benefit.	ICON7 translational cohort: 207 patients with epithelial ovarian cancer; discovery *n* = 91 and separate validation *n* = 116.	43/207 (discovery *n* = 25; validation *n* = 18).	No healthy control group. Standard carboplatin/paclitaxel versus the same regimen plus bevacizumab; prognostic comparisons used high versus low plasma miRNA abundance (median cut-off).	Full Cox model: age, histology, grade, FIGO stage, residual disease, treatment type and four candidate miRNAs. The final displayed model retained FIGO stage, residual disease, treatment, miR-1274A, miR-200b and miR-141.	Internal independent-sample validation within ICON7: *n* = 116 after discovery *n* = 91; not external validation.
Kapetanakis et al. (2015) [73]	miR-200b; also miR-21, miR-34a, miR-205	Positive versus negative miR-200b change, adjusted HR 2.326 (95% CI 0.828–6.535; *p* = 0.109); after additionally including baseline miR-200b, adjusted HR 2.953 (0.939–9.281; *p* = 0.064). Continuous time-dependent miR-200b: adjusted HR 1.057 per 10-unit increase (1.020–1.096; *p* = 0.003).	Kruskal–Wallis, linear regression, Kaplan–Meier, log-rank, Cox models, landmark analysis	miR-200b elevated in OC vs. benign lesions; longitudinal changes, rather than baseline levels, were associated with PFS and provided information beyond CA-125.	Prospective study of 101 women: ovarian cancer *n* = 51, benign pelvic tumours/lesions *n* = 25 and healthy women *n* = 25. Paired longitudinal samples were available for 33 patients; landmark analysis *n* = 24.	6/51 (FIGO I *n* = 4; II *n* = 2).	Expression: benign pelvic lesions *n* = 25 and healthy women *n* = 25. Prognosis: positive versus negative pre-/post-treatment miR-200b change; continuous time-dependent analysis used the 33 longitudinal patients.	FIGO stage (I–II vs. III–IV) and primary-treatment category (unresectable/chemotherapy only vs. resectable immediately or after neoadjuvant chemotherapy). The extended change model additionally included baseline miR-200b.	No independent validation cohort; longitudinal within-cohort assessment in *n* = 33 (landmark subset *n* = 24).
Pal et al. (2024) [72]	miR-27a, miR-182, miR-199a, miR-214, miR-591	Response-group OS differed for all five miRNAs (log-rank *p* = 0.001 to <0.001). Reported multivariable HRs were nonsignificant: miR-27a 1.091 (95% CI 0.458–2.601; *p* = 0.843), miR-214 1.621 (0.010–3.247; *p* = 0.172), miR-591 1.602 (0.740–3.467; *p* = 0.232), miR-182 1.250 (0.453–3.450; *p* = 0.667) and miR-199a 0.773 (0.198–3.023; *p* = 0.711).	*t*-test, MANOVA, Kaplan–Meier, Cox regression, ROC analysis	Individual miRNAs differed across treatment-response groups; all reported adjusted miRNA OS associations were nonsignificant. Response-group ROC analyses do not establish OC-detection accuracy or validate a combined five-miRNA model.	Prospective comparative study: 208 newly diagnosed advanced ovarian-cancer patients and 40 healthy controls; complete responders *n* = 72, partial responders *n* = 78 and non-responders *n* = 58.	0; eligibility was restricted to FIGO IIIB-IV.	Pretreatment expression/diagnostic comparison used 40 healthy controls; prognostic comparisons used complete, partial and non-responder groups within the 208-patient cohort.	Not reported. Table 3 labels Cox results multivariate/multivariable but does not identify the covariates in the miRNA models.	No development/validation split and no independent validation; ROC and survival analyses used the same cohort.
Robelin et al. (2020) [75]	11-miRNA panel: miR-15b-5p, miR-16-5p, miR-20a-5p, miR-21-5p, miR-93-5p, miR-122-5p, miR-150-5p, miR-195-5p, miR-200b-3p, miR-148b-5p, miR-34a-5p	No HRs reported. PFS medians were 13.2 versus 20.8 months for miR-15b-5p strata and 13.7 versus 24.8 months for miR-34a-5p strata; OS medians were 34.2 versus 52.0 months for miR-15b-5p and 34.3 months versus not reached for miR-93-5p (all *p* < 0.01).	ROC analysis, Wilcoxon signed-rank, Kaplan–Meier, log-rank, c-index, multivariate modelling	miRNA kinetics were variable and inferior to CA-125 for monitoring treatment; limited prognostic value overall.	CHIVA phase II trial subset: 119 patients with FIGO IIIC-IV ovarian carcinoma and 756 serial blood samples; 8 patients were used for 84-miRNA screening and the remaining 111 for 11-miRNA kinetic analyses.	0; all were FIGO IIIC or IV.	No cancer-free control. Within-cohort comparisons included complete versus incomplete interval debulking, RECIST CR/PR versus SD/PD, biomarker-decline strata and miRNA versus CA-125 kinetics; the parent trial compared placebo versus nintedanib added to chemotherapy.	None; reported survival associations were univariable c-index/Kaplan–Meier/log-rank analyses.	Within-cohort two-stage candidate selection: 84-miRNA screen in *n* = 8 followed by testing 11 selected miRNAs in *n* = 111; no independent external validation.
Uehara et al. (2024) [78]	PDS 11-miRNA model; NACT 3-miRNA model	Actual PFS did not differ significantly between matched PDS and NACT groups (log-rank *p* = 0.096). The main PDF reports LASSO coefficients, not individual HRs/95% CIs; those are referenced to Supplementary Table S5. Estimated median PFS improvement under the alternative recommended treatment was 11.7 months for seven actual-PDS patients and 32.6 months for 14 actual-NACT patients.	Cox regression (LOO-LASSO), Kaplan–Meier, log-rank, ROC, propensity score matching	Retrospective serum-miRNA models estimated PFS under alternative initial treatments. Predicted treatment gains were counterfactual model estimates, not observed benefits of miRNA-guided treatment; prospective validation is required.	Retrospective HGSOC source cohort *n* = 165; 130 had clinical stage IIIB-IVB. Main analysis *n* = 108 (PDS *n* = 36; NACT *n* = 72); propensity-score-matched analysis *n* = 48 (24 per treatment).	0 by pretreatment clinical eligibility in the 108-patient main analysis. One matched PDS patient was pathologically downstaged to IIB after surgery.	Primary debulking surgery versus neoadjuvant chemotherapy; propensity-score-matched comparison 24 versus 24.	The PDS and NACT PFS models used LOO-LASSO-selected miRNAs without conventional clinical covariates. Propensity matching used age, pretreatment CA-125, performance status, ascites, pleural fluid, peritoneal dissemination, upper-abdominal dissemination, retroperitoneal lymph-node metastasis, distant metastasis and clinical stage category.	Internal leave-one-out LASSO modelling in the matched cohort (PDS *n* = 24; NACT *n* = 24); no independent external validation.
Yoshida et al. (2021) [12]	miR-187-5p, miR-6870-5p, miR-1908-5p, miR-6727-5p, miR-6850-5p	Adjusted OS: index-OS2 HR 2.343 (95% CI 1.182–4.641; *p* = 0.015). Adjusted PFS: index-PFS2 HR 2.357 (1.289–4.311; *p* = 0.005). Individual univariable miRNA HRs were reported, but their 95% CIs appear only graphically.	Kaplan–Meier, log-rank, univariate/multivariate Cox regression, prognostic index modelling	Higher miR-187-5p and miR-6870-5p associated with poorer OS/PFS; combined miRNA indices were independent prognostic factors.	Retrospective GSE106817 analysis: 175 patients with HGSOC for OS and 173 for PFS (two lacked recurrence details). The source dataset also contained 969 healthy controls used for expression comparisons.	14/175 (FIGO I *n* = 4; II *n* = 10).	Prognosis: high versus low miRNA/index groups within HGSOC. The 969 healthy controls were used for expression comparisons, not survival modelling.	OS model: age, FIGO stage, residual tumour volume and index-OS2. PFS model: age, FIGO stage, residual tumour volume and index-PFS2.	Single retrospective dataset with no independent validation; the authors state that sample-based validation was not possible.
Zhong et al. (2025) [71]	miR-21, miR-145	Three-year binary prognosis (ORs, not survival HRs): miR-21 adjusted OR 1.882 (95% CI 1.526–2.856; *p* < 0.001) and miR-145 adjusted OR 1.522 (1.123–2.011; *p* < 0.001). The dependent-outcome/reference coding is not stated, and the positive miR-145 coefficient conflicts with the descriptive finding that lower miR-145 occurred among deceased patients.	Chi-square, *t*-test, logistic regression, ROC analysis	High miR-21 and low miR-145 associated with poor chemotherapy response and survival; combined model improved prognostic prediction (AUC 0.898).	Single-centre ovarian-cancer cohort *n* = 139; chemotherapy groups were partial response *n* = 62, stable disease *n* = 41 and progressive disease *n* = 36; three-year status was alive *n* = 103 and deceased *n* = 36.	86/139 (14 deceased plus 72 surviving patients).	No cancer-free control; comparisons were chemotherapy-response groups and deceased versus surviving patients at three years.	Age, differentiation, tumour stage, lymph-node metastasis, refractory ascites, miR-21 and miR-145.	Single-cohort model development/evaluation *n* = 139; no internal split or external validation.
Zhou et al. (2023) [20]	sEV miR-320a-3p, miR-378a-3p, miR-1307-3p and let-7d-3p; four-miRNA model with/without CA-125	Residual disease after cytoreductive surgery (R0 versus non-R0). Validation AUC: four-miRNA + CA-125, 0.903 (95% CI 0.846–0.945); Se 89.7%, Sp 91.0%.	LASSO selection; logistic modelling; leave-one-out cross-validation and separate validation set.	Predicts surgical residual disease; not a diagnostic cancer-versus-control AUC or a survival model.	284 participants/plasma samples: 221 advanced HGSOC, 20 early OC, 21 benign pelvic disease and 22 advanced colorectal cancer. Surgical tissues were additional specimens, not additional participants.	20 early-stage OC comparators; none in the advanced-HGSOC surgical model.	221 advanced HGSOC: 99 R0 and 122 non-R0.	Four-miRNA score and CA-125 in combined prediction model; clinical association analyses also considered age, stage, lymph-node status and TP53.	Advanced-HGSOC screening *n* = 37, discovery *n* = 30, validation *n* = 154; multicentre recruitment, but external validation by centre was not established.
Timofeeva et al. (2023) [31]	Plasma miR-17-5p, miR-20a-5p or miR-93-5p combined with piRNAs	Clinical chemotherapy response (RECIST).	Six miRNA-containing response models: source Table 8 models 2,3,5,6,7,8; sensitivity 64.29–92.86%, specificity 75–100%.	RECIST response prediction; same-cohort fitting without independent validation. piRNA-only models and tissue/PGR-phenotype analyses are not clinical circulating-miRNA outcome models.	38 women overall: 10 benign serous cystadenomas, 6 borderline tumours, 8 LGSOC and 14 HGSOC; treatment-response models use the cancer groups.	5/22 cancers stage I–II.	Responding versus nonresponding cancers.	No demonstrated adjustment for established clinical predictors.	Same-cohort model development; no independent validation.

Note: “Not reported” indicates that the item was not explicitly available in the assessed full text. Internal validation means a separate sample within the same study or parent trial, not external validation.

### 2.5. Dual Diagnostic and Prognostic Performance of Circulating miRNAs in OC

A subset of studies (*n* = 27) evaluated c-miRNAs with both diagnostic and prognostic relevance in OC (Table 3). These studies predominantly analysed serum or plasma samples across multiple FIGO stages and investigated both individual miRNAs and multi-miRNA signatures, with several incorporating established biomarkers such as CA-125.

Individual miRNAs demonstrated variable diagnostic performance, with reported AUCs generally ranging from approximately 0.70 to 0.93. Serum miR-145 distinguished patients with epithelial ovarian cancer from healthy controls with an AUC of 0.928 (95% CI: 0.86–0.95), whereas plasma miR-106a achieved an AUC of 0.879 (95% CI: 0.830–0.928) [41,79]. Serum miR-200b demonstrated an AUC of 0.743 when evaluated individually; its combination with CA-125 achieved 100% sensitivity and 93.1% specificity, although the AUC of the combined model was not reported [80]. Serum exosomal miR-484 achieved an AUC of 0.821, which increased to 0.912 when miR-484 was combined with CA-125 [81].

Selected multi-miRNA and multimodal models achieved high diagnostic performance, particularly when miRNAs were combined with CA-125 or evaluated using multivariable modelling. A seven-miRNA panel combined with CA-125 achieved an AUC of 0.97 overall and 0.93 for early-stage disease [9]. A model incorporating CA-125, miR-375, and miR-210 achieved an AUC of 0.984 [82], while a seven-miRNA small-extracellular-vesicle signature achieved AUCs of 0.913, 0.973, and 0.924 in its training, testing, and validation cohorts, respectively [83]. However, other multi-miRNA signatures in Table 3 demonstrated only moderate performance, and differences in cohort composition and validation design prevent direct comparisons with individual miRNAs.

Additional eligible reports provided heterogeneous evidence. Xu et al. reported a serum miR-21 survival association, but the reported ΔCt hazard-ratio direction was inconsistent with the narrative [18]. Zuberi et al. reported a serum miR-125b diagnostic AUC of 0.728 (95% CI 0.64–0.81), with no significant overall-survival association [19]. Zhou et al. evaluated a four-sEV-miRNA/CA-125 model for surgical residual disease, achieving an AUC of 0.903 (95% CI 0.846–0.945) in a separate validation set of 154 advanced-HGSOC patients [20]. This surgical-outcome AUC was excluded from the diagnostic comparison in Figure 8.

Günel et al. reported an AUC of 0.70 for detecting established recurrent EOC, without demonstrating prediction of future recurrence [21]. Suryawanshi et al. evaluated plasma classifiers for endometriosis-associated and serous OC; the expanded cohort included the discovery samples, so the findings do not constitute independent validation [22].

Several of these miRNAs were also associated with clinical outcomes. High serum miR-221 was reported as an independent adverse prognostic factor for OS [84], while higher exosomal miR-200b and miR-200c levels were associated with poorer OS, and higher miR-200c was additionally associated with shorter disease-free survival [39]. Lower miR-145 expression was associated with advanced disease and poorer survival, although an adjusted survival estimate was not reported [41]. Lower miR-125b expression was associated with adverse clinicopathological characteristics, poorer prognosis, and chemotherapy resistance [85]. Conversely, higher circulating miR-125b was associated with longer PFS and a favourable OS trend in another cohort [86]. Higher miR-26b expression was associated with a higher three-year survival rate, whereas lower miR-21 expression was associated with better survival; these findings were based on unadjusted survival comparisons without reported HRs [87].

Some studies also examined treatment-related or recurrence outcomes. A model combining miR-34a-5p with CA-125 was associated with the likelihood of optimal surgical cytoreduction, although this represented prediction of a surgical outcome rather than a time-to-event prognostic analysis [82]. Low serum exosomal miR-34a was associated with lymph-node metastasis and disease recurrence, but the study did not evaluate serial miRNA measurements for longitudinal monitoring [88].

**Figure 3:**
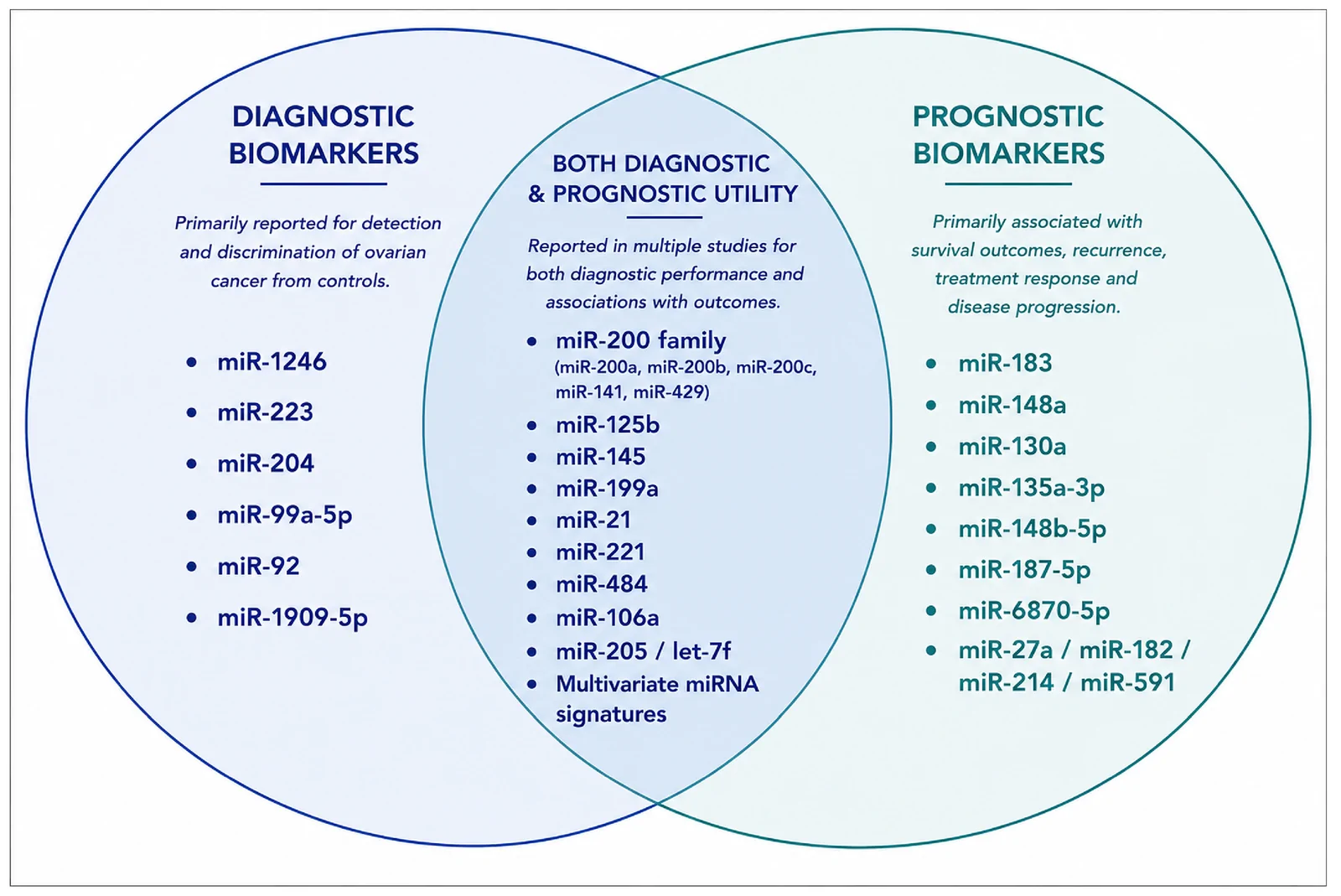
Venn diagram of circulating microRNAs with diagnostic and prognostic relevance in OC. The diagram illustrates the overlap of circulating microRNAs identified across studies as having diagnostic and/or prognostic utility in OC. The left circle represents miRNAs reported primarily in diagnostic studies, while the right circle represents those associated with prognostic outcomes, including overall survival, progression-free survival, treatment response, or recurrence. The overlapping region highlights miRNAs with dual diagnostic and prognostic relevance. Notably, several miRNAs, including members of the miR-200 family, as well as miR-21, miR-125b, and miR-145, were consistently reported across both domains, suggesting potential as multifunctional biomarkers. This is a selected-marker overview, not an exhaustive inventory; surgical-outcome model components are reported separately in Table 2.

Collectively, these findings suggest that c-miRNAs may have complementary diagnostic, prognostic, treatment-predictive, and recurrence-related applications in OC. The highest reported diagnostic AUCs were observed in selected multi-miRNA or multimodal models; however, this apparent advantage was not consistent across all studies and was not assessed through a formal pooled comparison. Furthermore, most prognostic findings lacked independent validation. To illustrate the overlap between the different clinical applications of c-miRNAs, a Venn diagram was constructed (Figure 3).

**Table 3:** Summary of c-miRNAs with combined diagnostic and prognostic utility in OC.

Author (Year)	miRNA/Signature	Diagnostic Performance (Including 95% CI)	Prognostic Findings (HR/OR, 95% CI, *p*-Value)	Key Conclusion	Study Population and Sample Size	Early-Stage Cases (FIGO I–II, *n*)	Control Composition	Validation Type and Validation Sample Size	Adjustment Variables
Chen et al. (2020) [85]	miR-125b ± CA-125	miR-125b: Se 76% (95% CI 75–85), Sp 41.6% (95% CI 26–55); combined with CA-125: AUC ≈ 0.90, Se 83.6%, Sp 95.6% (95% CI for AUC not reported).	Low miR-125b linked to advanced stage, LN metastasis, chemoresistance, poorer prognosis; higher post-surgery	miR-125b is downregulated in EOC; combining with CA-125 markedly improves diagnostic accuracy and predicts treatment response	Diagnostic analysis: 162 EOC, 35 borderline ovarian tumours, 40 benign ovarian disease and 42 healthy controls (*n* = 279). An additional treatment-monitoring cohort of 120 EOC patients was described; overlap with the diagnostic cohort was not stated.	30 FIGO I–II cases in the clinicopathological table (that table accounts for 152 EOC cases, whereas 162 EOC cases were reported for the diagnostic group).	35 borderline tumours, 40 benign ovarian disease and 42 healthy controls.	No independent validation cohort reported; postoperative/treatment-response assessment used an additional EOC cohort (*n* = 120; relationship to diagnostic cohort unclear).	Multivariate analysis was mentioned, but the variables entered and adjusted effect estimates were not reported.
Gahlawat et al. (2022) [9]	Total cf-miRNA + 7-miRNA panel	Seven-miRNA panel: AUC 0.87; benign vs. healthy AUC 0.77; early-stage AUC 0.81; panel+CA-125 AUC 0.97 overall and 0.93 early-stage. 95% CIs were not reported in the main article.	Higher total cf-miRNA was independently associated with worse OS (HR 1.83, *p* = 0.001; 95% CI not reported in the main article); no significant association with PFS.	cf-miRNAs show strong diagnostic and prognostic utility, particularly for early detection	244 participants: 100 ovarian cancer, 45 benign ovarian lesions and 99 age-matched healthy controls.	30 FIGO I–II cases (69 FIGO III–IV; stage unavailable for 1 case).	45 benign ovarian lesions and 99 age-matched healthy women.	Internal bootstrap validation (500 repeats; 80/20 training/test resampling). Diagnostic panel also examined in an external public sequencing dataset; no new external clinical cohort was recruited.	OS model adjusted for age, tumour grade, FIGO stage and CA-125; residual tumour was excluded because available for only 42 patients.
Gao & Wu (2015) [89]	miR-200c, miR-141	miR-200c: AUC 0.79, Se 72%, Sp 70%; miR-141: AUC 0.75, Se 69%, Sp 72%. 95% CIs not reported.	Low miR-200c: HR 3.14 (95% CI 1.67–5.93), *p* < 0.001; low miR-141: HR 1.83 (95% CI 1.00–3.33), *p* = 0.049 (unadjusted log-rank estimates).	Both miRNAs differentiate OC from controls and correlate with disease progression	143 participants: 74 epithelial ovarian cancer, 19 borderline ovarian tumours and 50 healthy controls.	54 FIGO I–II cases (33 stage I, 21 stage II); 20 FIGO III–IV cases.	19 borderline ovarian tumours and 50 healthy women.	Single case–control cohort; no separate validation sample reported.	Survival comparisons used Kaplan–Meier/log-rank analysis; no adjusted multivariable survival model was reported.
Guo et al. (2013) [90]	miR-92	AUC 0.803 (95% CI 0.735–0.871), Se 80.7%, Sp 75%.	Higher levels linked to advanced stage and LN metastasis	miR-92 is upregulated in EOC and shows moderate diagnostic accuracy	100 participants: 50 epithelial ovarian cancer and 50 healthy controls.	35 clinical stage I–II cases; 15 stage III–IV cases (UICC staging).	50 healthy controls; demographic matching was not performed.	Single prospective case–control cohort; no separate validation sample reported.	No multivariable prognostic model; associations were examined using Fisher’s exact test.
Habel et al. (2023) [91]	miR-21, miR-125b	Expression differences only; ROC/AUC, sensitivity, specificity and 95% CIs were not reported.	Lower miR-125b linked to chemotherapy resistance	miR-21 and miR-125b may serve as diagnostic and treatment-response biomarkers	74 participants: 49 newly diagnosed EOC patients and 25 age-matched healthy controls.	14 FIGO I–II cases; 35 FIGO III–IV cases.	25 age-matched healthy women with no personal/family cancer history.	Single observational case–control cohort; no separate validation sample reported.	No multivariable diagnostic or prognostic model reported.
Helmy et al. (2025) [80]	miR-200b ± CA-125/HE4/CRP	miR-200b: AUC 0.743, Se 63.3%, Sp 71%; miR-200b+CA-125: Se 100%, Sp 93.1%. AUC 95% CIs not reported.	High miR-200b linked to shorter EFS	miR-200b shows moderate standalone diagnostic value but excellent performance combined with CA-125	63 participants: 30 newly diagnosed EOC, 19 benign ovarian disease and 14 healthy controls.	Exact total not clearly reported in extractable text; treatment table lists early-stage categories but not a complete stage-count distribution.	19 benign ovarian disease and 14 healthy women.	Single cohort; no independent validation sample reported.	Cox regression included variables statistically significant in univariate analysis; the exact final covariate set was not clearly reported.
Hong et al. (2013) [84]	miR-221	Discriminant-function correct classification: cases/controls 88%, stage 77%, grade 76%; ROC/AUC and 95% CIs not reported.	High miR-221 was an independent adverse OS factor; multivariable model also retained FIGO stage (stage I–II vs. III–IV HR 5.075, *p* < 0.001). A 95% CI for miR-221 was not reported.	miR-221 shows both diagnostic and prognostic potential, though specificity may be limited	131 participants: 96 primary EOC and 35 healthy age-matched controls.	32 FIGO I–II cases (9 stage I, 23 stage II); 64 FIGO III–IV cases.	35 healthy age-matched volunteers.	Single retrospective cohort; discriminant classification was not tested in a separate validation cohort.	Cox model evaluated age, FIGO stage, tumour grade, histological type and serum miR-221.
Li et al. (2023) [83]	7-miRNA sEV signature	AUC 0.913 (training), 0.973 (testing), 0.924 (validation), and 0.903 for stage I disease; 95% CIs not reported.	High miR-141-3p and miR-200c-3p were associated with worse OS; RiskScore predicted PFS. HRs and 95% CIs were not reported.	sEVmiR-EOC model strongly differentiates EOC from benign disease and predicts prognosis	79 participants with adnexal masses: 46 malignant EOC and 33 benign tumours.	8 FIGO I–II cases; 38 FIGO III–IV cases.	33 histologically benign ovarian tumours; no healthy controls.	Chronological internal cohorts: training *n* = 29 (16 malignant, 13 benign), testing *n* = 20 (15 malignant, 5 benign), validation *n* = 30 (15 malignant, 15 benign).	Diagnostic model used LASSO-derived RiskScore. No clinical-covariate adjustment was reported for the miR-141-3p/miR-200c-3p survival comparisons.
Maeda et al. (2020) [88]	Exosomal miR-34a	No diagnostic ROC/AUC analysis.	Low miR-34a linked to LN metastasis and recurrence	miR-34a may serve as a biomarker for recurrence and disease progression	58 ovarian cancer patients; no non-cancer comparator group.	The text reports an early-stage comparison group of *n* = 25, while the stage table lists 21 stage I and 5 stage II cases; this internal discrepancy requires author clarification.	No healthy/benign control; within-cohort comparisons by recurrence and clinicopathological factors.	Single ovarian-cancer cohort; no separate validation sample reported.	Kaplan–Meier/log-rank and clinicopathological comparisons; no adjusted multivariable survival model reported.
Meng et al. (2015) [40]	Four-miRNA serum classifier: miR-7, miR-25, miR-93 and miR-429 (seven miRNAs assayed)	Four-miRNA classifier: AUC 0.980 (source Figure 1); sensitivity 93%, specificity 92%; AUC 95% CI not reported.	miR-429: univariate HR 1.7837 (95% CI 1.13–2.81); it remained independently prognostic with FIGO stage and residual tumour in multivariable analyses.	Multi-miRNA panel demonstrates strong diagnostic and prognostic value	246 participants: 180 EOC patients and 66 healthy women.	32 FIGO I–II cases; 147 FIGO III–IV cases; stage unavailable for 1 case.	66 healthy women.	Internal random split of the EOC cohort: training 60% (*n* = 108) and test 40% (*n* = 72); allocation of healthy controls between sets was not clearly reported.	Cox models evaluated miR-429 with FIGO stage and residual tumour; clinical factors including age, grade, histology and CA-125 were also assessed.
Meng et al. (2016) [39]	Exosomal miR-373/200 family panel	miR-200a AUC 0.914; miR-200b AUC 0.815; combined panel AUC 0.925, Se 88%, Sp 90%. 95% CIs not reported.	Adjusted OS HRs: miR-373, 2.9 (95% CI 1.3–6.8), *p* = 0.012; miR-200b, 2.8 (1.1–6.8), *p* = 0.029; miR-200c, 2.5 (1.1–6.1), *p* = 0.038. Adjusted recurrence-free survival for miR-200c was nonsignificant: HR 1.7 (0.8–3.6), *p* = 0.171.	Exosomal miRNAs discriminate malignant vs. benign disease and correlate with progression	215 participants: 163 EOC, 20 benign ovarian disease and 32 healthy women.	27 FIGO I–II cases (16 stage I, 11 stage II); 118 stage III–IV; stage unknown for 18/163 (source Table 2).	20 benign ovarian disease and 32 healthy women.	Single clinical cohort; analyses were repeated in a high-grade EOC subgroup (*n* = 112), not an independent validation cohort.	The reported multivariable survival analysis included FIGO stage, CA-125, residual tumour and eligible miRNAs; grade was assessed in univariable analysis but has no multivariable estimate in source Table 1.
Minareci et al. (2024) [92]	miR-1181, miR-4314	miR-4314 AUC 0.78 (95% CI 0.69–0.85); miR-1181 AUC 0.76 (95% CI 0.67–0.86); combined AUC 0.81 (95% CI 0.70–0.90).	High miR-4314: OS HR 2.78 (95% CI 1.3–6.0), *p* = 0.007; DFS HR 2.3 (95% CI 1.3–4.1), *p* < 0.004. High miR-1181: OS HR 5.4 (95% CI 2.0–14.9), *p* < 0.001; DFS HR 3.2 (95% CI 1.6–6.2), *p* < 0.001.	Both miRNAs show moderate diagnostic accuracy and strong prognostic relevance	135 participants: 69 EOC and 66 healthy controls.	19 FIGO I–II cases (15 stage I, 4 stage II); 50 FIGO III–IV cases.	66 healthy controls.	Single case–control cohort; no separate validation sample reported.	Kaplan–Meier/log-rank estimates; no adjusted multivariable survival model reported.
Ranmale et al. (2025) [93]	Three-miRNA diagnostic panel: miR-182, miR-106a and miR-23b (ten miRNAs assayed)	Three-miRNA serum panel: AUC 0.743; sensitivity 81.6%, specificity 60.5%; 95% CI not reported.	Serum miR-130a: adjusted PFS HR 0.64 (95% CI 0.46–0.90), *p* = 0.010; *n* = 78, 41 PFS events. The source’s tissue miR-20a association is not circulating-miRNA prognostic evidence.	Three-miRNA diagnostic discrimination was moderate; serum miR-130a was associated with PFS in the same cohort.	127 participants: 83 ovarian cancer and 44 healthy controls.	36 FIGO I–II cases (11 stage I, 25 stage II); 47 FIGO III–IV cases (44 stage III, 3 stage IV).	44 healthy controls.	Single observational cohort; no independent validation sample reported.	Multivariable Cox models included 10 miRNAs, age, treatment mode, FIGO stage, CA-125, lymph-node metastasis, malignant ascites and chemotherapy-response score.
Shah et al. (2018) [82]	Multi-miRNA panel + CA-125	CA-125+miR-375 AUC 0.956; adding miR-210 AUC 0.984. 95% CIs not reported.	miR-34a-5p with CA-125 predicted surgical cytoreduction outcome; no time-to-event HR was reported.	Combining miRNAs with CA-125 improves diagnostic and prognostic assessment	86 participants: 56 HGSOC and 30 age-matched healthy volunteers.	9 FIGO I–II cases (4 stage I, 5 stage II); stage unknown for 8 cases.	30 age-matched healthy women.	Internal random split: training *n* = 43 (28 HGSOC, 15 healthy) and test *n* = 43 (28 HGSOC, 15 healthy).	Stepwise logistic models combined CA-125 with selected miRNAs (miR-375±miR-210 for diagnosis; miR-34a-5p+CA-125 for cytoreduction outcome).
Song et al. (2020) [87]	miR-26b, miR-21	miR-26b AUC 0.753 (95% CI 0.708–0.825); miR-21 0.826 (0.821–0.932). Combined AUC 0.905 (0.817–0.953) in source Table III; the source narrative instead states 0.916. Table III [87] is used for extraction; the discrepancy is unresolved.	miR-26b and miR-21 expression groups were associated with survival by Kaplan–Meier analysis; HRs and adjusted estimates were not reported.	Combined miRNA signature improves diagnosis and correlates with tumour progression	172 participants: 86 ovarian cancer and 86 healthy controls.	34 FIGO I–II cases (12 stage I, 22 stage II); 52 FIGO III–IV cases.	86 individuals undergoing physical examination; described as healthy controls.	Single case–control cohort; no separate validation sample reported.	Survival comparisons were unadjusted; no multivariable Cox model reported.
Yunusova et al. (2023) [94]	Exosomal miR-24-3p, miR-101	Expression/correlation analyses only; ROC/AUC and 95% CIs not reported.	miR-101 was associated with FIGO stage; no survival HR/OR was reported.	Exosomal miRNAs may serve as liquid biopsy biomarkers but require validation	39 participants: 20 ovarian cancer and 19 healthy controls.	2 FIGO I–II cases (both stage IIB); 18 stage III cases (3 IIIB, 15 IIIC).	19 healthy women.	Single case–control cohort; no separate validation sample reported.	No multivariable diagnostic or prognostic model reported.
Zhang et al. (2020) [81]	Exosomal miR-484 ± CA-125	miR-484 AUC 0.821, Se 77.9%, Sp 80%; miR-484+CA-125 AUC 0.912. 95% CIs not reported.	For OS: miR-484 RR 4.34 (95% CI 2.75–6.28), *p* = 0.010. For PFS: RR 3.96 (95% CI 2.28–5.92), *p* = 0.014; both adjusted for the listed clinicopathological variables.	miR-484 is a promising diagnostic and independent prognostic biomarker	173 participants: 113 ovarian cancer and 60 healthy controls.	64 FIGO I–II cases; 49 FIGO III–IV cases.	60 healthy women.	Single case–control cohort; no separate validation sample reported.	Cox models included histological grade, lymph-node metastasis, FIGO stage and serum exosomal miR-484.
Zhao et al. (2018) [79]	miR-106a	AUC 0.879 (95% CI 0.830–0.928), Se 72.9%, Sp 98.0%.	High miR-106a independently predicted poor OS (adjusted HR 4.985, *p* = 0.009); FIGO stage also remained independent (HR 3.976, *p* = 0.021). 95% CIs not reported.	miR-106a shows strong diagnostic and prognostic utility	195 participants: 95 EOC and 100 healthy controls.	44 FIGO I–II cases; 51 FIGO III–IV cases.	100 healthy volunteers.	Single case–control cohort; no separate validation sample reported.	Cox analysis evaluated age, histological type, tumour differentiation, FIGO stage, CA-125 and miR-106a; significant variables entered the multivariable model.
Zheng et al. (2013) [95]	miR-205 + let-7f	Panel phase I: AUC 0.831 (95% CI 0.772–0.880); stage-I AUC 0.895 (0.822–0.967). Phase II: AUC 0.813 (0.759–0.860); stage-I AUC 0.783 (0.699–0.853).	Lower let-7f was associated with poorer survival; a fully adjusted HR with 95% CI was not reported in the extractable main text.	miR-205 and let-7f improve OC detection and show prognostic relevance	560 plasma samples from two institutions: 360 EOC and 200 healthy controls.	Phase-specific stage-I analyses were reported, but an overall FIGO I–II total across all 360 cases was not explicitly stated.	200 healthy controls across screening, training and validation phases.	Multiphase design: screening pools; training 76 EOC/30 controls; validation phase I and an independent validation phase II. Phase II included 150 EOC and 100 controls.	Survival analyses assessed let-7f with clinicopathological variables; the exact adjusted covariate set was not clearly reported in the main-text extraction.
Zhu et al. (2017) [86]	miR-125b	AUC 0.737, Se 75.6%, Sp 68.5%; 95% CI not reported.	Higher miR-125b was associated with longer PFS and a favourable OS trend; HRs and 95% CIs were not reported.	miR-125b shows moderate diagnostic accuracy and prognostic potential	189 participants: 135 EOC and 54 benign ovarian tumour controls.	33 FIGO I–II cases; 102 FIGO III–IV cases.	54 benign ovarian tumour patients; no healthy controls.	Microarray discovery (18 EOC/16 benign) followed by qRT-PCR assessment in the full cohort (135 EOC/54 benign); this was technical/internal validation, not independent external validation.	Survival analyses were unadjusted; no multivariable Cox model reported.
Zuberi et al. (2016) [42]	miR-199a	miR-199a AUC 0.704, Se 69.1%, Sp 95.7%; 95% CI not reported.	Low miR-199a was associated with metastasis and poorer survival; HR/OR and 95% CI were not reported.	miR-199a is downregulated and associated with advanced disease	140 participants: 70 EOC and 70 healthy controls.	33 FIGO I–II cases (23 stage I, 10 stage II); 37 FIGO III–IV cases.	70 healthy women.	Single case–control cohort; no separate validation sample reported.	No adjusted multivariable survival model reported.
Zuberi et al. (2020) [41]	miR-145	miR-145 AUC 0.928 (95% CI 0.86–0.95), Se 69.1%, Sp 95.7%.	Low miR-145 was associated with advanced disease and poorer survival; no adjusted survival HR with 95% CI was reported.	miR-145 demonstrates strong diagnostic accuracy and prognostic relevance	140 participants: 70 EOC and 70 healthy controls.	33 FIGO I–II cases (23 stage I, 10 stage II); 37 FIGO III–IV cases.	70 healthy women.	Single case–control cohort; no separate validation sample reported.	No adjusted multivariable survival model reported.
Xu et al. (2013) [18]	Serum miR-21	Higher expression in EOC than healthy controls; no diagnostic ROC-AUC, sensitivity or specificity reported.	Adjusted OS HR 2.327, *p* = 0.019; 95% CI not reported. HR labelled for continuous ΔCt; direction/coding is inconsistent with the narrative and cannot be interpreted reliably as high versus low expression.	Exploratory prognostic association; diagnostic accuracy not quantified.	134 participants: 94 primary EOC and 40 healthy controls; retrospective Chinese cohort.	32/94 (stage I, 9; stage II, 23).	40 age-matched healthy women.	Single cohort; no independent validation.	Age, FIGO stage, tumour grade and histological type.
Zuberi et al. (2016) [19]	Serum miR-125b	AUC 0.728 (95% CI 0.64–0.81); Se 62.3%, Sp 77.1%; cutoff 3.76.	No significant OS association by Kaplan–Meier analysis; adjusted HR and 95% CI not reported.	Moderate case–control discrimination; no demonstrated survival association.	140 participants: 70 EOC and 70 healthy controls; Indian single-centre cohort.	33/70 (stage I, 23; stage II, 10).	70 age-matched healthy women.	Single case–control cohort; probable overlap with Zuberi reports [41,42], not independent validation.	No adjusted multivariable survival model.
Shapira et al. (2014) [23]	Plasma miRNA profiles; miR-760, miR-223, miR-195, miR-122 and miR-497 in survival comparisons	Plasma expression differences versus benign/healthy comparators; no diagnostic AUC.	Differential expression versus healthy/benign comparators; no diagnostic AUC. Survival-group AUCs 0.13, 0.15, 0.73, 0.74 and 0.87 are not diagnostic AUCs.	Exploratory same-cohort analysis; no independent validation. Ultracentrifuged plasma supernatant, not the isolated EV fraction.	42 serous OC, 36 benign masses and 23 healthy controls; survival comparison: 19 surviving <2 years versus 7 >4 years.	6/42 stage II in Table 1; source stage listings differ between tables.	Noncancer controls for diagnosis; clinical outcome groups within OC.	No independent validation.	No demonstrated clinical-confounder-adjusted validation.
Sun et al. (2024) [30]	Plasma miR-296-3p	Higher plasma expression in OC and in paclitaxel-resistant versus sensitive cases	Higher plasma expression in OC and in paclitaxel-resistant versus sensitive cases; no eligible AUC or adjusted clinical effect estimate.	Only patient plasma comparisons retained; CAF/EV functional experiments do not establish clinical EV diagnostic accuracy.	46 OC and 23 healthy controls; paclitaxel-response comparison: 13 sensitive and 8 resistant cases. Overlap not specified.	Not reported for the diagnostic plasma cohort; response subset advanced-stage.	Noncancer controls for diagnosis; clinical outcome groups within OC.	No independent validation.	No demonstrated clinical-confounder-adjusted validation.
Timofeeva et al. (2022) [32]	Plasma miR-16-5p, miR-17-5p, miR-20a-5p and miR-93-5p	Plasma expression differences; no cancer-versus-noncancer AUC.	Plasma expression comparisons; no cancer-versus-noncancer AUC. Surgical-outcome model AUCs 0.77/0.77/0.74/0.73; chemotherapy-response model AUCs 0.91/0.90/0.90/0.90 (Figure 7).	Four models per clinical endpoint, fitted and evaluated in the same small cohort; no independent validation. Tissue-derived diagnostic AUCs excluded.	122 women overall across healthy, benign, borderline and serous-cancer groups; clinical plasma model cohort: 20 HGSOC (source Table 5).	6/20 HGSOC stage I–II in Table 5.	Noncancer controls for diagnosis; clinical outcome groups within OC.	No independent validation.	No demonstrated clinical-confounder-adjusted validation.

Abbreviations: Se, sensitivity; Sp, specificity.

### 2.6. Integrated Evidence Map of c-miRNA Biomarkers

**Figure 4:**
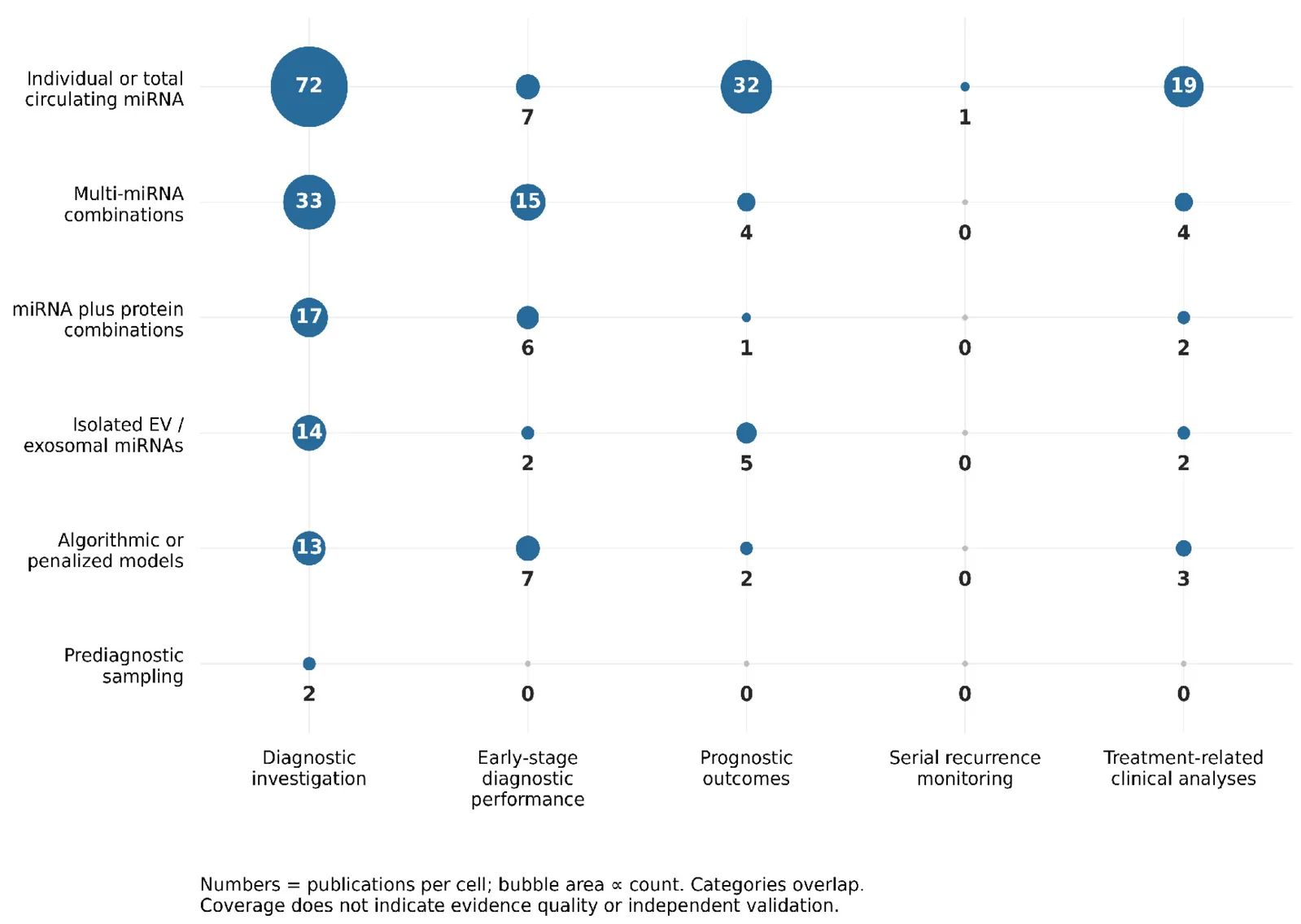
Descriptive evidence map of circulating-miRNA research in ovarian cancer. Numbers indicate publications contributing an eligible result to each cell; blue bubble area is proportional to the count, and grey markers indicate zero. Publications can contribute to multiple cells. Counts represent coverage, not evidence strength, risk of bias or independent validation. Early-stage performance requires a stage-specific cancer-versus-noncancer analysis. Prediagnostic investigation includes a case-only time-to-diagnosis study. Operational definitions, supporting references and study-level qualifications are provided in Supplementary Table S3.

An evidence gap map was created to show how the included publications were distributed across the main circulating miRNA biomarker categories and their clinical applications in OC (Figure 4). Study-level classifications used to generate the map are provided in Supplementary Table S3.

Most of the evidence focused on diagnostic applications, particularly studies evaluating individual or total circulating miRNAs, multi-miRNA combinations, and miRNA–protein combinations. Fewer studies specifically investigated diagnostic performance in early-stage OC, although multi-miRNA combinations were the most widely studied approach in this setting. Prognostic studies mainly focused on individual or total circulating miRNAs. In contrast, relatively few studies evaluated EV- or exosome-derived miRNAs, algorithm-based models, or combined miRNA–protein approaches for prognostic purposes. Clear evidence gaps were identified in serial monitoring for recurrence and in the analysis of samples collected before diagnosis. Studies related to treatment outcomes also focused mainly on individual circulating miRNAs.

Overall, the evidence was unevenly distributed across clinical applications. Longitudinal monitoring, prediagnostic evaluation, and validation in clinically relevant patient populations therefore remain important priorities for future research.

The technical landscape of the included studies reveals significant methodological variations across biofluid sources, analytical platforms, and data processing standards. These findings highlight a critical lack of standardization within the field, which may impede the clinical reproducibility of c-miRNA biomarkers in OC (Table 4).

### 2.7. Risk of Bias

Risk of bias was assessed at the level of the eligible result because several studies contributed diagnostic-accuracy, prognostic-factor, and/or prediction-model evidence and therefore appeared in more than one assessment. In total, 132 result-level assessments are tabulated. Domain judgments and supporting notes are provided in Supplementary Table S4.

**Figure 5:**
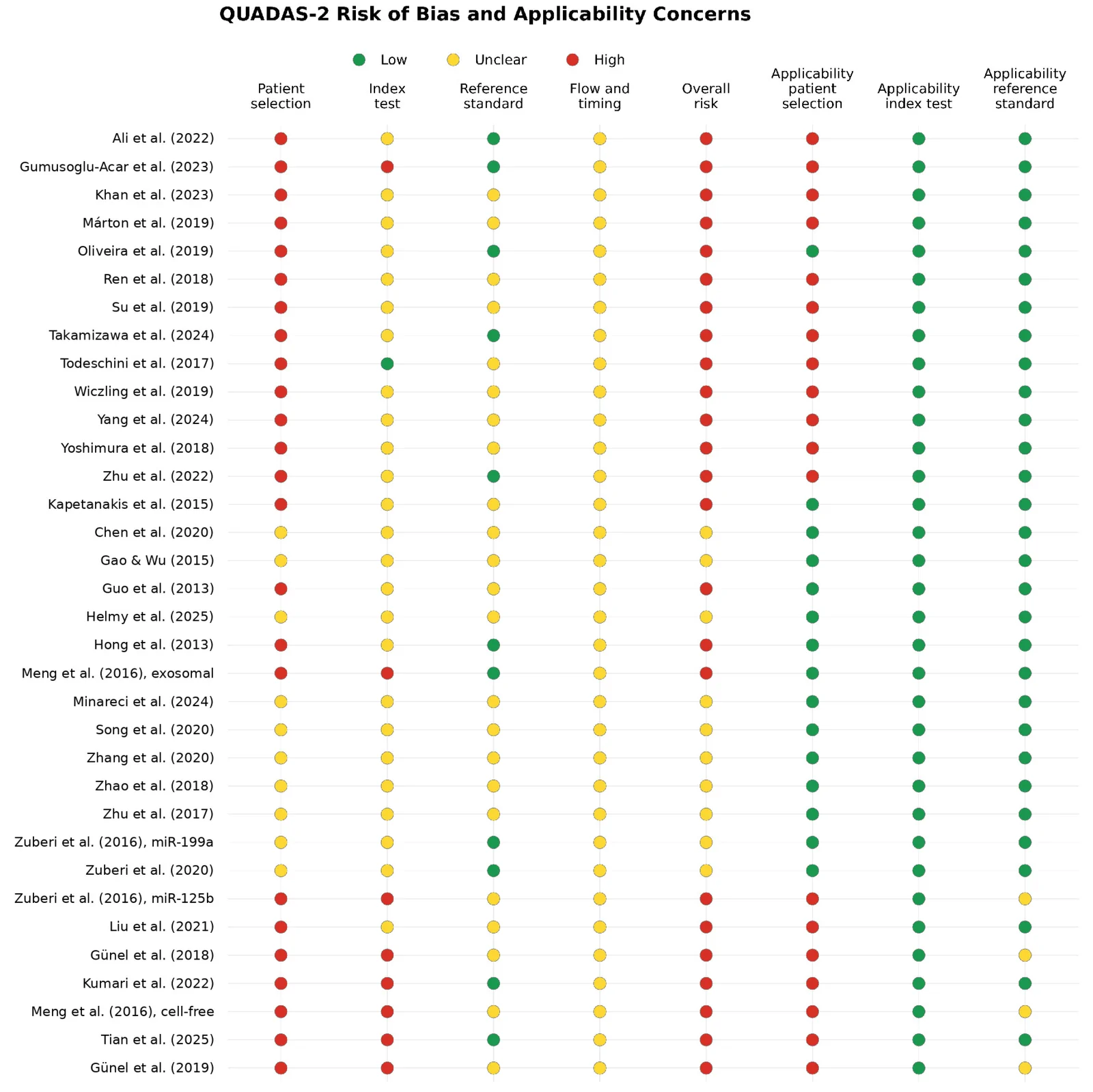
Risk-of-bias assessment using QUADAS-2. Traffic-light plot presenting domain-level and overall risk-of-bias judgments, together with applicability concerns, for 34 diagnostic-accuracy publications [3,6,10,19,21,24,25,29,35,39,41,42,44,46,47,48,56,57,58,60,62,66,67,73,79,80,81,84,85,86,87,89,90,92]. Green indicates low risk or low applicability concern, yellow indicates unclear risk or concern, and red indicates high risk or high applicability concern. The Meng et al. (2016) reports are distinguished by their exosomal [39] and cell-free [25] miRNA measurements; the Zuberi et al. (2016) reports are distinguished by their miR-199a [42] and miR-125b [19] biomarkers.

QUADAS-2 was applied to 34 diagnostic-accuracy publications. Twenty-four were judged at high overall risk of bias and 10 at unclear risk; none was judged low overall. The principal concern was patient selection, particularly two-gate or case–control sampling with separately recruited cancer and control groups. Index-test conduct and flow and timing were frequently unclear because threshold prespecification, blinding, participant exclusions, or the interval between index and reference assessments were incompletely reported (Figure 5). Reports contributing expression comparisons without eligible diagnostic-accuracy estimates were retained in the qualitative synthesis but were not assigned QUADAS-2 assessments.

**Figure 6:**
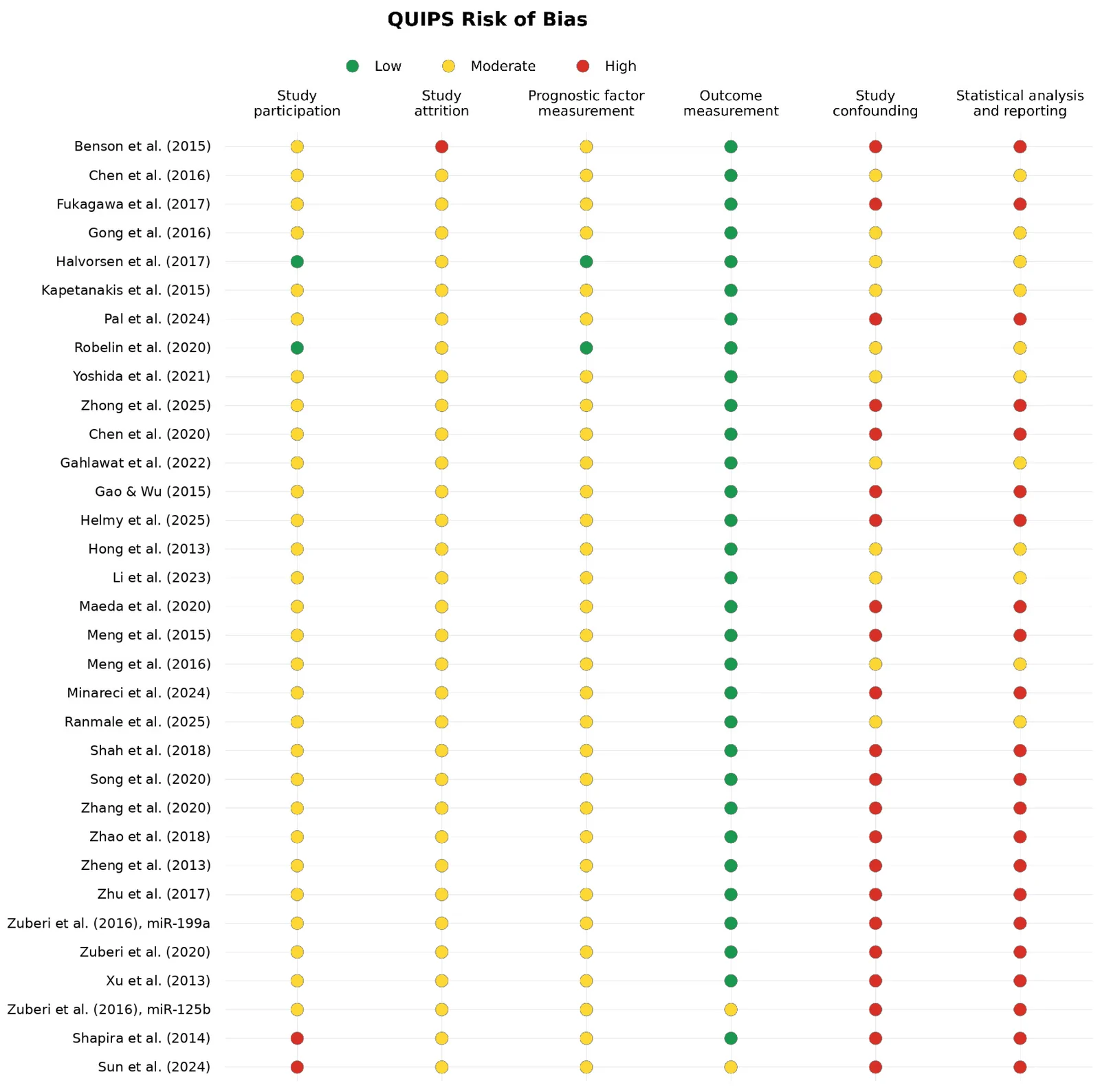
Risk-of-bias assessment using QUIPS. Traffic-light plot presenting domain-level judgments for 33 prognostic-factor assessments [9,12,18,19,23,30,39,40,41,42,43,69,70,71,72,73,75,76,77,79,80,81,82,83,84,85,86,87,88,89,92,93,95]. Green indicates low risk of bias, yellow indicates moderate risk, and red indicates high risk. The Zuberi et al. (2016) reports are distinguished by their miR-199a [42] and miR-125b [19] biomarkers.

QUIPS was applied to 33 prognostic-factor publications. Because QUIPS does not prescribe a single overall study-level rating, findings are reported by domain. High risk was most frequent for study confounding and statistical analysis and reporting (22/33 publications in each domain), whereas outcome measurement was judged low risk in 31 publications and moderate risk in two. Study participation, attrition, and prognostic-factor measurement were most commonly judged moderate risk because representativeness, missing data, normalization, blinding, or cut-point prespecification were incompletely reported (Figure 6).

**Figure 7:**
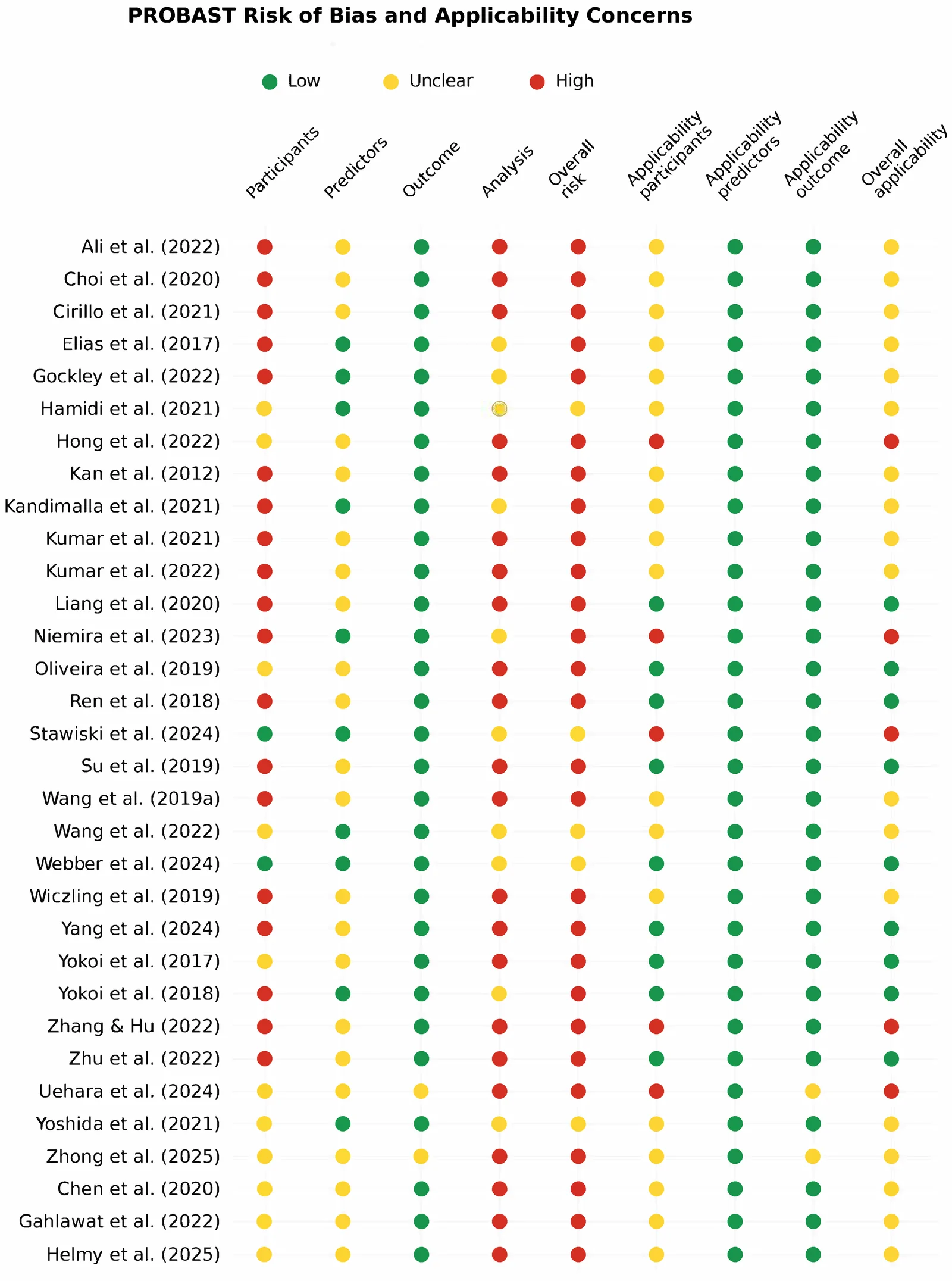

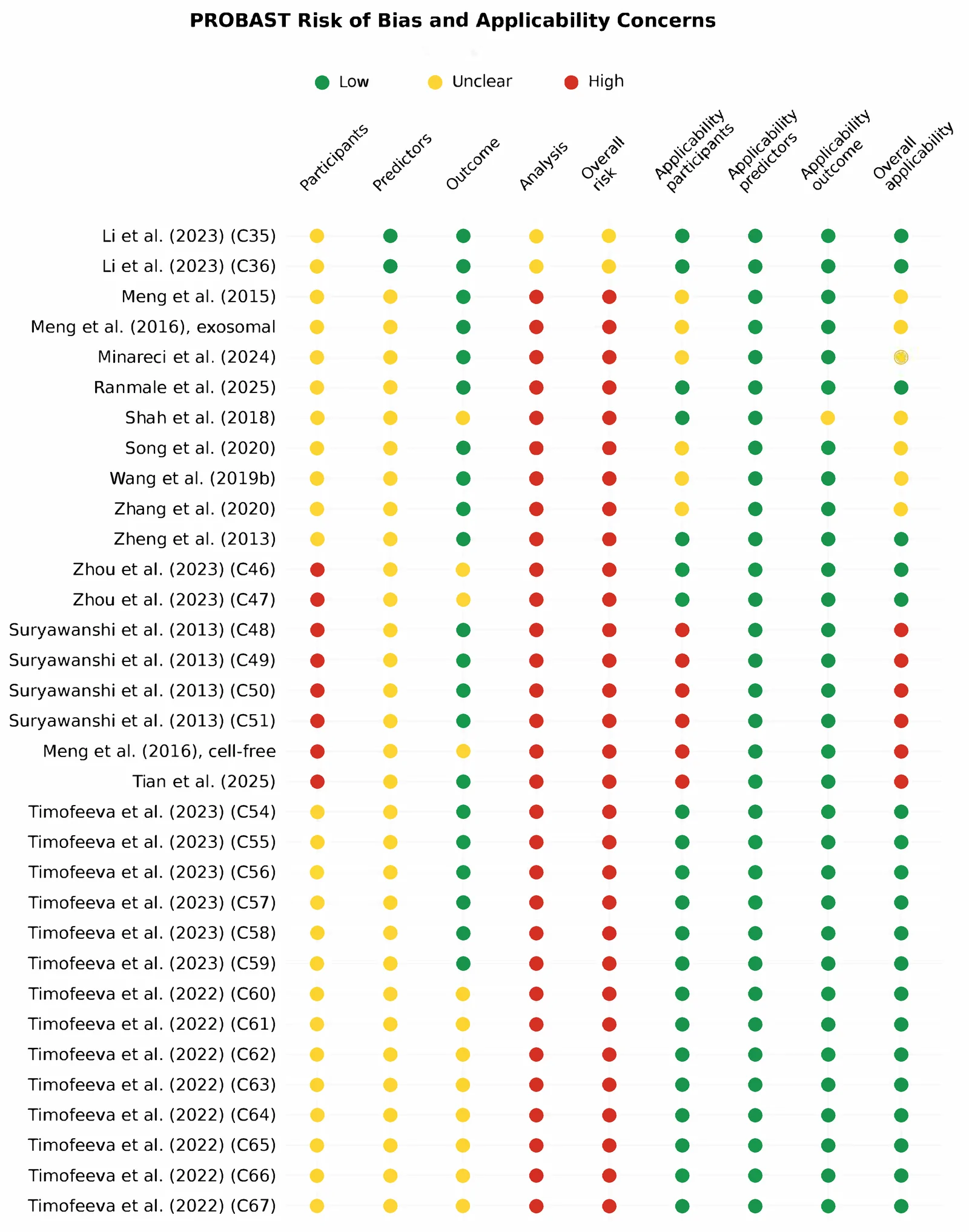
PROBAST assessments of 65 multivariable models [2,3,4,5,6,8,9,10,11,12,13,14,20,22,25,29,31,32,36,37,38,39,40,47,49,50,51,52,53,54,55,56,57,58,59,65,68,71,78,80,81,82,83,85,87,92,93,95]. Green: low risk/concern; yellow: unclear; red: high. Risk of bias and applicability are assessed separately. Separate models from one publication appear as separate entries; rationales are provided in Supplementary Table S4. The Meng et al. (2016) reports are distinguished by their exosomal [39] and cell-free [25] miRNA measurements.

PROBAST was applied to 65 multivariable diagnostic or prognostic model evaluations. Fifty-eight evaluations were judged at high overall risk of bias and seven at unclear risk; none was judged low overall. The Analysis domain was the main driver of high-risk judgments because of small development samples, data-driven predictor selection, limited internal or external validation, incomplete handling of missing data, absent calibration, and/or incomplete model specification. Statistical predictor selection was assigned to the Analysis domain and was not double-counted in the Predictors domain. Applicability was judged separately (Figure 7).

## 3. Discussion

This systematic review provides an updated and comprehensive synthesis of the current evidence on c-miRNAs and multivariate biomarker signatures in OC, integrating findings from 91 publications across diagnostic, prognostic, and multimodal applications. Overall, our findings suggest that c-miRNAs represent a promising class of non-invasive biomarkers, with multivariate and multimodal approaches generally showing higher reported AUC values than single miRNAs, particularly in early-stage disease detection and prognostic stratification.

### 3.1. Diagnostic Performance and Comparison with Existing Evidence

Our findings demonstrate that individual circulating miRNAs (c-miRNAs) generally exhibit moderate diagnostic accuracy, whereas multi-miRNA panels and multimodal biomarker signatures generally reported higher apparent performance, frequently exceeding AUC values of 0.90 [2,8,55]. These observations are consistent with previous systematic reviews and meta-analyses reporting superior diagnostic performance for miRNA panels compared with individual biomarkers [16]. However, the improved performance of multi-marker signatures is biologically plausible rather than merely additive. OC is a highly heterogeneous disease involving multiple dysregulated molecular pathways, including cell proliferation, apoptosis, angiogenesis, immune modulation, epithelial–mesenchymal transition, and metastasis. Individual miRNAs typically regulate only a subset of these processes, whereas combining multiple miRNAs allows a biomarker panel to capture a broader spectrum of tumour biology, thereby reducing the impact of biological variability between patients and improving diagnostic robustness.

Our review further indicates that integrating circulating miRNAs with established protein biomarkers, particularly CA-125 and HE4, produced some of the highest reported diagnostic-performance estimates. In an independent validation cohort, a model combining miR-320b and miR-141-3p with CA-125 and HE4 achieved an AUC of 0.989 overall and 1.00 for stage I–II disease [2]. A model combining miR-193a-5p with CA-125 and HE4 achieved an AUC of 0.996, although it was evaluated without independent validation [3]. Other studies also reported improved diagnostic performance after combining circulating miRNAs with CA-125 and/or HE4 [5,6,47]. These findings suggest that miRNAs and protein biomarkers may provide complementary discriminatory information. However, the included studies did not directly establish the biological mechanisms responsible for the observed improvements. Multimodal models may partly compensate for the limited sensitivity of CA-125 in early-stage disease and its imperfect specificity when distinguishing OC from benign gynecological conditions [1,96]. Nevertheless, the very high performance estimates should be interpreted cautiously because several models were developed in relatively small case–control cohorts and lacked independent external validation.

Compared with earlier systematic reviews and meta-analyses [1,16], our review includes more recent studies employing high-throughput miRNA profiling and multivariable computational methods to develop diagnostic models [8,36,54,55,65]. These methods included logistic regression, neural networks, relative-expression-ordering classifiers, random forests, artificial neural networks, XGBoost, and other machine-learning approaches. Although several models reported very high apparent discrimination, with some AUCs approaching 1.00 [8,36,55], these findings should be interpreted cautiously. Some studies used relatively small or highly selected case–control cohorts and compared patients with OC against healthy controls, potentially creating favourable conditions for inflated estimates of diagnostic performance. Machine-learning and other multivariable models developed using limited samples and numerous candidate predictors are particularly vulnerable to overfitting, whereby a model captures cohort-specific patterns rather than reproducible disease-associated signals [97]. This concern is consistent with our PROBAST assessment, in which 58 of 65 model evaluations were judged to have a high overall risk of bias. Furthermore, independent external validation was uncommon, limiting confidence that the reported performance would generalize to broader clinical populations, including patients with benign adnexal disease. Exceptionally high AUC values may therefore partly reflect model-development procedures, selected comparator groups, or other methodological characteristics rather than performance achievable in routine clinical practice.

A previous pooled analysis reported a sensitivity of 76%, specificity of 81%, and summary ROC AUC of 0.85 for circulating miRNAs in OC [16]. A more recent meta-analysis focused on exosomal miRNAs reported a pooled sensitivity of 82%, specificity of 81%, and summary ROC AUC of 0.88 [17]. Several multivariable models included in the present review reported study-specific AUCs above 0.90. However, these AUCs should not be directly compared numerically with pooled sensitivity or specificity estimates, and comparisons with summary ROC estimates remain descriptive because of differences in study populations, specimens, comparator groups, thresholds, and validation designs. The high performance reported by some recent models may reflect advances in biomarker discovery, sequencing technologies, and multivariable analytical methods, but it may also partly arise from study design and patient selection. In particular, case–control studies comparing patients with established OC against healthy controls may produce more favourable estimates than studies involving women with benign adnexal masses or indeterminate pelvic lesions. Such comparator-related spectrum and selection effects limit applicability to routine diagnostic populations [97,98]. Consequently, clinical utility cannot be established until these models are evaluated in prospective, multicentre studies involving representative screening or symptomatic referral populations.

A particular strength of this review is its detailed evaluation of early-stage OC detection, for which improvements in diagnostic performance could be clinically important. Although only two included studies primarily recruited patients with early-stage disease, several others reported stage-specific subgroup analyses. Yokoi et al. reported that their ten-miRNA model classified 95.1% of stage I cases as positive, although a separate stage I-specific AUC was not provided [8]. Webber et al. reported 80% sensitivity for early-stage cancers in an external validation cohort [4]. Yang et al. reported that plasma exosomal miR-223 combined with CA-125 distinguished stage I–II epithelial ovarian cancer from healthy controls with an AUC of 0.944 (95% CI: 0.899–0.990), 97.8% sensitivity, and 88.5% specificity [56]. These findings suggest that multianalyte approaches warrant further evaluation for early-stage detection. Nevertheless, early-stage sample sizes were frequently limited, mixed-stage cohorts predominated, and some analyses used healthy individuals rather than clinically relevant benign or symptomatic controls. These limitations reduce confidence in the generalizability of the reported performance and highlight the need for prospective validation in screening or symptomatic referral populations before clinical implementation.

Finally, the evidence map (Figure 4) highlights the concentration of research on diagnosis and the limited coverage of serial recurrence monitoring. Prospective validation and assessment of clinical utility remain priorities.

This imbalance suggests that future research should move beyond biomarker discovery toward prospective validation, assessment of clinical utility, and investigation of how circulating miRNAs can inform disease monitoring, treatment response, and patient prognosis.

**Figure 8:**
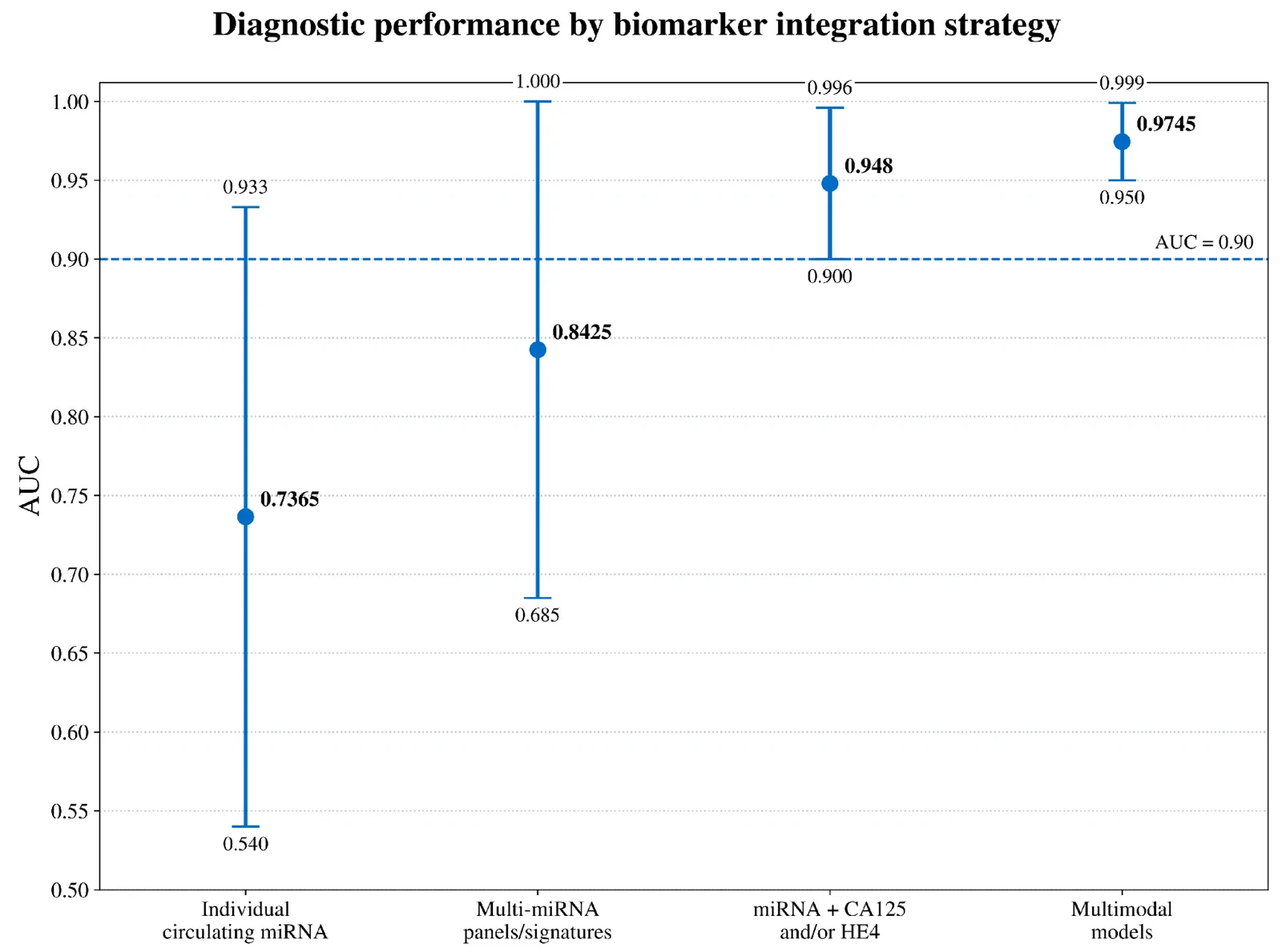
Reported diagnostic performance according to biomarker integration strategy. Diagnostic AUC values were grouped into four predefined categories: individual circulating miRNAs, multi-miRNA panels/signatures, miRNA models combined with CA-125 and/or HE4, and multimodal models incorporating miRNAs with additional protein or clinical variables. Each publication contributed a maximum of one study-level representative AUC per category. Validation or test-set estimates were prioritized over training or discovery estimates; when multiple eligible AUCs were reported within the same study and category, the median was used. The vertical bars represent the observed minimum-to-maximum range of the study-level representative AUCs within each category, and the plotted point represents the arithmetic midpoint of that range, calculated as (minimum + maximum)/2. No weighting by sample size, study quality, or precision was applied. The underlying raw AUC values and study-level calculations are provided in Supplementary Table S5.

The distribution of reported diagnostic AUCs according to biomarker-integration strategy is summarized descriptively in Figure 8. Study-level representative AUCs ranged from 0.540 to 0.933 for individual circulating miRNAs and from 0.685 to 1.00 for multi-miRNA panels or signatures. The substantial overlap between these categories indicates that multi-miRNA panels did not uniformly outperform individual miRNAs. In contrast, the representative AUCs included in the miRNA plus CA-125 and/or HE4 category ranged from 0.900 to 0.996, while those of the multimodal models ranged from 0.950 to 0.999. These distributions suggest a pattern of higher reported diagnostic performance with greater biomarker integration, particularly for models incorporating protein biomarkers or additional clinical variables. However, Figure 8 represents an unweighted descriptive summary rather than a meta-analysis: the plotted points are arithmetic midpoints of the observed minimum-to-maximum ranges and do not represent pooled or average effect estimates. Moreover, the categories differed in the contributing studies, populations, comparator groups, sample sizes, and validation strategies. Exceptionally high AUCs should therefore be interpreted cautiously because several were derived from selected case–control cohorts, used healthy controls, or lacked independent external validation, potentially leading to overestimation of performance in routine clinical populations.

### 3.2. Prognostic Value and Clinical Implications

Across the included studies, associations between circulating miRNAs and clinical outcomes were dependent on the specific miRNA, biospecimen, analytical method, and clinical context. Higher circulating miR-21 levels were associated with poorer chemotherapy response or survival in some cohorts [71,87], whereas lower miR-145 expression was associated with adverse outcomes [41,71]. Lower miR-125b expression was associated with poorer prognosis and chemotherapy resistance in one study [85], while another reported that higher miR-125b levels were associated with longer PFS and a favourable OS trend [86]. Findings for the miR-200 family were less consistent: higher exosomal miR-200b and miR-200c levels were associated with poorer survival in one study [39], whereas lower circulating miR-200c and miR-141 levels were associated with poorer outcomes in another [89]. These differences indicate that the prognostic direction of a miRNA should not be generalized across specimen types, patient populations, or analytical methods.

Longitudinal profiling and multivariable miRNA models may nevertheless provide clinically relevant prognostic information. Serial changes in plasma miR-200b were associated with PFS, whereas baseline miR-200b was not significantly prognostic [73]. Serum miR-130a decreased following surgery and increased in four patients who subsequently developed recurrence, sometimes before an increase in CA-125 [74]. Other studies examined miRNA kinetics during first-line treatment or after experimental therapy [75,76], while multivariable prognostic indices and treatment-specific miRNA models were evaluated in separate cohorts [12,77,78]. However, these approaches differed substantially in design, outcome definition, modelling strategy, and validation, and their incremental value over established clinical predictors remains uncertain.

Prognostic biomarker research was more heterogeneous than diagnostic research in the included literature. The investigated endpoints included OS, PFS, disease-free or recurrence-related outcomes, chemotherapy response, surgical outcome, and platinum resistance, which represent distinct clinical events and should not be considered interchangeable. Follow-up duration and the number of outcome events also varied considerably. Patient populations differed in disease stage, histological subtype, treatment regimen, and residual disease following surgery, while blood samples were collected at different time points, including before treatment, after surgery, during chemotherapy, and throughout follow-up. These differences limit direct comparisons among studies and are likely to contribute to the inconsistent prognostic findings.

Several studies evaluated serial changes in circulating miRNAs during treatment or follow-up [73,74,75,76]. These studies suggest that circulating miRNA levels may change during surgery, chemotherapy, disease progression, or recurrence and that serial measurements may provide information not captured by a single baseline sample. Nevertheless, the evidence was mixed. In the study by Robelin et al., the kinetics of 11 circulating miRNAs were heterogeneous and generally less informative for treatment monitoring than CA-125 [75]. Therefore, circulating miRNAs should currently be considered potential complementary biomarkers rather than replacements for CA-125.

The available evidence remains insufficient to support the routine clinical implementation of circulating miRNAs as prognostic biomarkers. Many studies used modest, single-centre cohorts, investigated different miRNA candidates, applied heterogeneous analytical methods, and lacked independent external validation. Several prognostic associations were based only on Kaplan–Meier or univariable analyses, while some multivariable studies did not clearly report their covariates or adjusted effect estimates [43,71,72,74,75]. Relatively few studies demonstrated that circulating miRNAs provided prognostic information independent of established variables such as disease stage, histological subtype, residual disease, and treatment regimen [12,39,69,70,73,77,79,84]. Evidence that miRNAs add clinically meaningful prognostic value to existing clinicopathological models therefore remains limited.

Overall, circulating miRNAs show potential for prognostic stratification, treatment-response assessment, and longitudinal disease monitoring. Future research should prioritize large prospective multicentre cohorts, standardized outcome definitions, harmonized sampling and analytical protocols, prespecified prognostic models, adequate numbers of outcome events, serial specimen collection, and independent external validation. These studies should also evaluate whether adding circulating miRNAs to established clinical models improves patient classification and leads to better treatment or follow-up decisions.

### 3.3. Dual Biomarker Function and Biological Relevance

Several circulating miRNAs were investigated across both diagnostic and prognostic or treatment-response studies, particularly members of the miR-200 family [39,44,59,73,80,89], miR-21 [63,71,87,91], miR-125b [85,86,91], and miR-145 [41,71]. Their repeated identification across different clinical settings supports their further evaluation as candidate biomarkers. However, recurrence across observational studies does not by itself establish that these circulating miRNAs are causal regulators of ovarian cancer biology. Furthermore, the direction of some associations was not consistent across studies. For example, higher exosomal miR-200b and miR-200c levels were associated with poorer outcomes in one study [39], whereas lower circulating miR-200c and miR-141 levels were associated with poorer survival in another [89].

The overlap between diagnostic and prognostic associations is nevertheless biologically plausible. Members of the miR-200 family have been investigated in relation to epithelial–mesenchymal-transition-associated processes [44], while miR-21, miR-125b, and miR-145 have been associated with ovarian cancer development, clinicopathological characteristics, treatment response, and survival [41,71,85,86,87,91]. Because individual miRNAs can influence multiple cellular pathways, changes in their circulating concentrations may be associated with both the presence of disease and its subsequent clinical course. However, most studies included in this review were observational biomarker studies and did not directly establish the molecular mechanisms responsible for the reported clinical associations.

Circulating miRNA measurements may also provide information about changes occurring during treatment and follow-up. Serial plasma miR-200b measurements were associated with progression-free survival, whereas the baseline measurement alone was not significantly associated with this outcome [73]. Serum miR-130a decreased after surgery and increased in four patients who subsequently developed recurrence, sometimes before an increase in CA-125, although this finding was based on a very small number of recurrent cases [74]. Other longitudinal studies reported heterogeneous miRNA kinetics, and an 11-miRNA panel generally provided less effective treatment monitoring than CA-125 [75]; treatment-associated changes were also reported in a small pilot study of platinum-resistant disease [76]. These results support further investigation of serial miRNA measurements, but they do not yet establish their ability to detect minimal residual disease, emerging chemoresistance, or recurrence before radiological evidence.

Relatively few of these candidate biomarkers have undergone rigorous independent external validation. Differences in biospecimen type, collection and processing procedures, analytical platforms, normalization strategies, comparator groups, and patient populations remain important sources of variability [1,15]. In addition, because individual miRNAs can be dysregulated in multiple diseases and biological processes, their disease specificity may be inadequate when they are used in isolation. Their clinical value may therefore be greater when they are incorporated into carefully validated multimiRNA or multimodal models rather than used as standalone biomarkers [1,16].

Overall, the repeated investigation of the miR-200 family, miR-21, miR-125b, and miR-145 across diagnostic and prognostic settings identifies them as candidates for further validation. Nevertheless, circulating concentrations should not be assumed to represent active participation in ovarian cancer pathogenesis, because they may reflect tumour-cell release, extracellular-vesicle secretion, treatment effects, systemic responses, or changes in blood-cell composition. Future prospective studies should determine whether these candidates provide reproducible and incremental clinical value for diagnosis, prognostic stratification, treatment monitoring, and recurrence surveillance.

### 3.4. Emerging Trends: Exosomal miRNAs and Machine Learning Models

An important emerging trend identified in this review is the increasing investigation of extracellular-vesicle-derived miRNAs, including miRNAs described as exosomal, as ovarian cancer biomarkers. Encapsulation within extracellular vesicles may protect miRNAs from extracellular degradation and can enrich molecular signals associated with their cells of origin. However, the available evidence does not yet establish that EV-associated miRNAs consistently provide greater diagnostic specificity than total circulating miRNAs, particularly because few studies have performed direct comparisons using identical patient populations and analytical methods.

A recent systematic review identified 27 studies examining exosomal miRNAs for ovarian cancer detection, of which 16 cohorts contributed diagnostic data to the meta-analysis. The pooled sensitivity was 0.82 (95% CI: 0.76–0.86), the pooled specificity was 0.81 (95% CI: 0.75–0.86), and the area under the summary ROC curve was 0.88 [17]. Although these estimates indicate promising diagnostic performance, the authors emphasized the need for methodological standardization and prospective validation.

Individual EV-miRNA studies also reported encouraging findings. A serum small-EV model comprising seven miRNAs, including several members of the miR-200 family, achieved an AUC of 0.903, with 80% sensitivity and 100% specificity, for distinguishing stage I epithelial ovarian cancer from benign ovarian disease. In the same analysis, CA-125 achieved an AUC of 0.694, with 60% sensitivity and 78.8% specificity [83]. Separately, a combination of exosomal miR-200a, miR-200b, and miR-200c distinguished epithelial ovarian cancer from benign ovarian disease with an AUC of 0.925, 88% sensitivity, and 90% specificity [39]. However, that study did not directly compare the miRNA panel with CA-125 because CA-125 measurements were unavailable for the benign and healthy comparator groups [39].

Integrated profiling of ascites- and plasma-derived EVs has also been used to identify multimiRNA signatures. The eight-miRNA ovarian cancer extracellular-vesicle-miRNA (OCEM) signature developed by Wang et al. achieved an AUC of 0.9762 in the plasma training set and 0.9375 in the internal plasma validation set for distinguishing ovarian cancer from benign disease [53]. Although the signature was derived by identifying overlapping miRNAs in ascites and plasma, its reported plasma performance was based on internal rather than independent external validation. Therefore, its reproducibility in prospectively recruited clinical populations remains to be established.

High-throughput sequencing and comprehensive profiling platforms can facilitate the discovery of miRNA signatures that might not be identified through candidate-miRNA approaches. Nevertheless, their application is affected by sequencing cost, preanalytical and batch effects, analytical complexity, differences between platforms, and the risk of overfitting when large numbers of molecular features are evaluated in small cohorts. Consequently, signatures discovered through high-throughput profiling require confirmation using standardized targeted assays and independent clinical cohorts before implementation.

In parallel, machine-learning methods are becoming increasingly prominent in ovarian cancer miRNA-biomarker research. Yokoi et al. analysed 4046 serum samples that passed quality control and developed a ten-miRNA model that achieved an AUC of 1.00, with 99% sensitivity and 100% specificity, in an independent validation cohort [8]. The model classified 95.1% of stage I cases as positive, although the study did not report a separate stage I-specific AUC, sensitivity, or specificity [8].

Stawiski et al. subsequently evaluated a previously identified 14-miRNA signature using XGBoost in prediagnostic serum samples from 80 women who later developed ovarian cancer [65]. The model distinguished samples collected less than two years before diagnosis from samples collected 2–7 years before diagnosis, achieving a median test-set AUC of 0.771 across repeated holdout analyses. This result suggests that the miRNA profile changed as the time to diagnosis shortened; however, because the study did not include cancer-free controls in this analysis, it did not establish the ability of the signature to detect ovarian cancer prospectively in a screening population [65]. The investigators also reported that differences in sequencing platforms and batch effects prevented direct transfer and external validation of the previously developed model.

Machine-learning methods can evaluate complex and potentially nonlinear relationships among multiple biomarkers. Nevertheless, their performance can be overestimated when feature selection and model development are conducted in small or highly selected datasets. Robust evaluation therefore requires appropriate separation of training and testing data, prevention of information leakage, calibration assessment, transparent reporting of model development, comparison with conventional statistical approaches, and independent external validation in clinically representative populations [65,97].

Preliminary findings presented in a 2025 ASCO conference abstract described an automated high-throughput EV-based multivariate index assay with 92% sensitivity and 93% specificity for all-stage ovarian cancer and 90% sensitivity for stage I disease [99]. The assay was reported to outperform CA-125; however, these findings are currently available only as a conference abstract and should be considered preliminary until complete information concerning cohort selection, comparator composition, model development, statistical comparisons, and independent validation is available in a full peer-reviewed publication.

Overall, EV-associated miRNA profiling and machine-learning-based analysis represent promising directions for ovarian cancer biomarker development. However, they should not yet be characterized as established improvements over conventional circulating-miRNA assays or clinical biomarkers. Their clinical value will depend on standardized EV isolation and miRNA measurement, transparent model development, independent external validation, and prospective evaluation in populations representative of real diagnostic practice.

### 3.5. Methodological Limitations and Risk of Bias

The result-specific assessment identified substantial but uneven methodological limitations. Among the 34 diagnostic-accuracy publications assessed with QUADAS-2, 24 were judged at high overall risk of bias and 10 at unclear risk. The principal concern was patient selection: two-gate or case–control sampling may exaggerate separation between cases and controls and thereby inflate diagnostic-accuracy estimates [98]. Incomplete reporting was judged unclear rather than high unless the available information supported a high-risk judgment. Heterogeneity in sample processing, analytical platforms, and normalization strategies further limits comparability across studies and complicates meta-analytic synthesis [17].

For prognostic-factor associations, QUIPS concerns were concentrated in study confounding and statistical analysis and reporting, with 22 of 33 publications judged high risk in each domain. These judgments reflected limited adjustment for relevant clinicopathological factors, small samples, data-derived cut points, and incomplete reporting. Under PROBAST, 58 of 65 model evaluations were judged at high overall risk of bias and seven at unclear risk, driven mainly by the Analysis domain. Consequently, apparently excellent AUCs and prognostic separation should be interpreted cautiously until the models undergo adequately powered prospective and external validation with complete model specification and calibration reporting [17,97].

These findings are consistent with previous systematic reviews, which highlight substantial heterogeneity, lack of standardization, and reproducibility challenges in miRNA biomarker research, underscoring the need for rigorous study design and transparent reporting [1,17].

### 3.6. Clinical Translation and Future Directions

While c-miRNAs demonstrate considerable potential as non-invasive biomarkers, clinical translation remains limited. A key barrier is the lack of large-scale prospective validation studies, particularly in real-world screening settings. In addition, the absence of standardized workflows for sample collection, processing, and data analysis continues to hinder reproducibility.

Recent efforts in the field are increasingly focused on large-scale, multicentered validation frameworks and the development of standardized operating procedures, aiming to improve reproducibility and facilitate clinical implementation. In parallel, there is a growing shift towards integrative, multi-analyte approaches that combine miRNA signatures with complementary biomarker modalities and clinical data, often supported by advanced computational and machine-learning techniques. Future research should therefore prioritize:

- Prospective, multicentered validation studies
- Standardization of pre-analytical and analytical protocols
- Integration of miRNA signatures with complementary biomarker modalities and clinical data
- Development of clinically applicable, cost-effective diagnostic platforms

This shift is also reflected in recent studies employing emerging bioassays, including exosomal miRNA profiling, multi-miRNA panels, multimodal models that combine miRNAs with CA-125 or HE4, and high-throughput sequencing supported by advanced bioinformatics pipelines.

### 3.7. Strengths and Limitations of the Present Review

This review has several strengths, including adherence to PRISMA guidelines, comprehensive database searches, dual independent screening and data extraction, and result-specific use of validated risk-of-bias tools: QUADAS-2, QUIPS, and PROBAST. Applying tools to the target result allowed a study contributing more than one type of evidence to be assessed appropriately. The inclusion of recent high-throughput and machine learning-based studies provides an updated perspective on this rapidly evolving field. The review also has limitations. Substantial clinical and methodological heterogeneity precluded quantitative meta-analysis; the review was restricted to published studies; and risk-of-bias judgments were sometimes limited by incomplete reporting in the primary reports. Two potentially relevant reports could not be retrieved; their eligibility could not be assessed (Supplementary Table S6).

## 4. Materials and Methods

### 4.1. Protocol and Reporting Standards

The Systematic Review was prospectively registered in PROSPERO (CRD420251230898, https://www.crd.york.ac.uk/prospero/ (accessed on 28 September 2026)). We performed and presented this review in accordance with the Preferred Reporting Items for Systematic Reviews and Meta-Analyses (PRISMA) guidelines [100].

### 4.2. Eligibility Criteria

Studies were considered eligible if they evaluated c-miRNAs or miRNA-based biomarker signatures in women with histologically confirmed ovarian cancer of any histological subtype, or with suspected ovarian malignancy subsequently verified by histopathology, including studies using prediagnostic samples from women subsequently diagnosed with OC. Acceptable biofluids included serum, plasma, whole blood, and exosome-derived fractions. Eligible study designs comprised case–control, cohort, cross-sectional, and biomarker discovery and validation studies.

Studies were excluded if they were restricted to animal or in vitro models, tissue-only miRNA analyses, or genetic variant investigations without measurement of c-miRNAs. We also excluded case reports, narrative or systematic reviews, and conference abstracts lacking sufficient extractable data. Studies evaluating circulating miRNAs as predictors of treatment response, surgical outcomes, or disease-course outcomes, including chemotherapy response, platinum resistance, progression, recurrence, or survival, were eligible. Studies reporting only treatment-induced changes in miRNA expression without an association with a clinical outcome were excluded. Studies predicting residual disease after cytoreductive surgery or informing surgical versus neoadjuvant treatment selection were eligible; these outcomes were analysed separately from cancer detection and survival. Additional exclusions applied to studies assessing miRNAs solely in non-blood biofluids (e.g., urine), and purely computational target/pathway predictions without analysis of clinical blood-miRNA measurements. Secondary analyses of patient-level serum, plasma or whole-blood miRNA datasets were eligible when they evaluated the clinical biomarker question; newly collected experimental samples were not required. Whole-blood measurements were eligible, whereas measurements restricted to purified blood-cell populations were outside the circulating-biomarker scope. This distinction between clinical-data reanalysis and mechanistic in silico prediction was clarified during revision. Studies primarily concerned with assay or technology development for miRNA detection or identification, rather than evaluation of circulating miRNAs as clinical biomarkers, were outside the review scope.

### 4.3. Search Strategy

A comprehensive search of MEDLINE/PubMed, Scopus, and CENTRAL was conducted from inception to 30 May 2026. The search combined MeSH terms and keywords related to OC, microRNAs, liquid biopsy, diagnosis, and prognosis. Reference lists of included studies and relevant reviews were screened to identify additional studies. Only studies in the English language were included in this systematic review.

### 4.4. Study Selection

Two reviewers (KA, MP) independently screened titles and abstracts using Rayyan [101], followed by full-text assessment of potentially eligible studies. Any disagreements were resolved through discussion and, when necessary, consultation with a third reviewer (CP). Duplicate records were identified and removed both manually and through Rayyan’s automated features. The study selection process is summarized in a PRISMA flow diagram (Figure 1). During manuscript revision, potential cohort overlap was assessed by comparing authors, institutions, recruitment periods, participant numbers, biospecimen types and cohort descriptions. Publications based on overlapping or potentially overlapping cohorts were linked and were not interpreted as independent validation.

### 4.5. Data Extraction

Data were extracted independently by the two reviewers (KA, MP) using a piloted and standardized data extraction form. The information collected included key study characteristics (author, year, country, and design), as well as study details such as sample size, control groups, FIGO stage, and histological subtype.

We also recorded methodological aspects of miRNA analysis, including the type of biofluid used (e.g., serum, plasma, whole blood, or exosomal fractions), the miRNA fraction analysed (cell-free, exosomal, or total circulating), and the analytical platforms employed (such as qRT-PCR, microarray, or next-generation sequencing). Where reported, normalization strategies were also documented. For Figure 2G, analytical-platform categories were mutually exclusive and based on the primary discovery or profiling platform. Studies using NGS or microarray for discovery followed by qRT-PCR validation were classified as NGS or microarray, respectively. PCR-based screening arrays were classified as qRT-PCR. NanoString, digital PCR and FirePlex were coded separately. Initial tissue-based candidate discovery was retained where it defined the discovery workflow; in Niemira et al., NanoString defined primary serum profiling and tissue RNA-seq was ancillary. The study-level assignments are included in Supplementary Table S3. Blood-matrix categories were based on the clinical blood fraction; ancillary non-blood specimens were recorded separately.

Details on the specific miRNAs or miRNA panels investigated were extracted, including whether studies focused on individual miRNAs or multivariate signatures, and whether these were evaluated alone or in combination with established protein biomarkers (e.g., CA-125, HE4). For diagnostic studies, we collected performance metrics such as area under the curve (AUC), sensitivity, specificity, and, where available, performance in early-stage disease. For prognostic studies, outcomes of interest included overall survival (OS), progression-free survival (PFS), treatment response, and recurrence, along with the corresponding statistical analyses (e.g., hazard ratios, Kaplan–Meier curves, and multivariable models).

Information on validation approaches (internal or external cohorts), the use of machine learning methods, and longitudinal or paired-sample designs was also extracted when available. Any disagreements between reviewers were resolved through discussion until consensus was reached.

### 4.6. Risk of Bias Assessment

Risk of bias was assessed according to the target result rather than by assigning one tool to each report. Diagnostic-accuracy results for individual or combined index tests were assessed with QUADAS-2 [98]; prognostic-factor association results were assessed with QUIPS [102]; and multivariable diagnostic or prognostic prediction-model results were assessed with PROBAST [97]. A study contributing more than one eligible result type could therefore be assessed with more than one tool, and distinct eligible model evaluations from the same report were assessed separately. ROBINS-I was not used because the review did not synthesize causal effects of non-randomized interventions. The original assessments were performed independently by two reviewers, with disagreements resolved by discussion and consensus. The authors verified the additional extractions and assessments for Xu, Zuberi, Zhou, Günel and Suryawanshi during revision. QUADAS-2 evaluates patient selection, the index test, the reference standard, and flow and timing, with applicability assessed for the first three domains. QUIPS evaluates study participation, attrition, prognostic-factor measurement, outcome measurement, confounding, and statistical analysis and reporting. PROBAST evaluates participants, predictors, outcome, and analysis, with applicability assessed separately. Unreported information was not assumed to indicate high risk; it was judged unclear in QUADAS-2 or PROBAST, or moderate in QUIPS, unless the reported methods supported another judgment. This approach represents an amendment to the original PROSPERO registration, which specified ROBINS-I. The amendment was made following peer-review feedback and methodological reconsideration and is documented in the PROSPERO record.

### 4.7. Data Synthesis

Owing to substantial clinical and methodological heterogeneity across the included studies, results were synthesized narratively rather than through meta-analysis. Studies were grouped according to their primary clinical application as diagnostic, prognostic, or combined diagnostic–prognostic investigations. Study characteristics and individual findings were tabulated, while the distribution of study designs, populations, analytical platforms, biospecimen sources, and risk-of-bias assessments was summarized visually.

For Figure 8, reported diagnostic AUC values were classified as individual miRNAs, multi-miRNA panels, miRNA+CA-125/HE4 models, or multimodal models. Each study contributed a maximum of one representative AUC per category; when multiple eligible AUCs were reported within the same category, their median was used. The minimum and maximum study-level AUCs defined the displayed range, while the plotted point represented the arithmetic midpoint of this range, calculated as (minimum + maximum)/2. No weighting was applied, and the figure should be interpreted as a descriptive summary rather than a meta-analytic estimate. The underlying AUC values are provided in Supplementary Table S5.

## 5. Conclusions

C-miRNAs, particularly when integrated into multimodal biomarker signatures, represent a promising approach for the detection and prognostic evaluation of OC. Among the candidates identified, members of the miR-200 family and miR-205 consistently appear across diagnostic, prognostic, and combined models, suggesting that they may form a core component of clinically relevant biomarker panels.

At the same time, the current body of evidence is characterized by important methodological limitations. Many studies rely on case–control designs and relatively small cohorts, which may lead to overestimation of diagnostic performance and limit the generalizability of findings. This is reflected in the frequent reporting of very high accuracy metrics in early-phase studies, which have yet to be confirmed in larger, prospectively designed cohorts.

An emerging trend in recent studies is the shift towards using exosomal or small extracellular vesicle (sEV)-derived miRNAs. These approaches may offer improved specificity and better reflect tumour biology, although further work is needed to determine their added value in clinical settings.

While much of the literature has focused on diagnostic applications, our findings also highlight the potential of specific miRNAs, such as miR-125b and miR-130a, for prognostic applications, including predicting recurrence and treatment response. These applications may ultimately be of greater clinical relevance, particularly in guiding patient management and therapeutic decision-making.

Overall, substantial heterogeneity in sample handling, analytical platforms, and normalization strategies remains a major challenge, limiting comparability across studies. Addressing these issues will require greater standardization, as well as well-designed prospective studies with appropriate clinical integration and external validation. Such steps are essential if miRNA-based biomarkers are to progress from promising research findings to tools that can be reliably implemented in clinical practice.

## Figures and Tables

**Table 4:** Methodological characteristics of the studies included in the systematic review. Percentages use 91 publications.

Methodological Variable	Category	Frequency/Number of Studies
Blood matrix	Serum	63.7% (*n* = 58)
	Plasma	33.0% (*n* = 30)
	Serum and plasma	1.1% (*n* = 1)
	Whole blood	1.1% (*n* = 1)
Blood matrix	Blood, unspecified	1.1% (*n* = 1)
Platform	qRT-PCR	61.5% (*n* = 56)
	NGS	17.6% (*n* = 16)
	Microarray	16.5% (*n* = 15)
Platform	NanoString	2.2% (*n* = 2)
Platform	Digital PCR	1.1% (*n* = 1)
Platform	FirePlex	1.1% (*n* = 1)
Normalization	Reported	85.7% (*n* = 78)
	Not reported	14.3% (*n* = 13)

## Data Availability

No new primary data were generated. All study-level data extracted and analysed during this systematic review are provided in the article and its Supplementary Materials.

## Supplementary Materials

The following supporting information can be downloaded at: https://www.mdpi.com/article/10.3390/ijms27198823/s1.

## Abbreviations

The following abbreviations are used in this manuscript:

AUC	area under the receiver operating characteristic curve
CA-125	cancer antigen 125
cf-miRNA	cell-free microRNA
cfRNA	cell-free RNA
CI	confidence interval
c-miRNA	circulating microRNA
DFS	disease-free survival
EOC	epithelial OC
EV	extracellular vesicle
FIGO	International Federation of Gynecology and Obstetrics
HE4	human epididymis protein 4
HGSOC	high-grade serous OC/carcinoma
HR	hazard ratio
IOTA	International Ovarian Tumour Analysis
miRNA	microRNA
NPV	negative predictive value
OC	ovarian cancer
OS	overall survival
PFS	progression-free survival
PPV	positive predictive value
ROC	receiver operating characteristic
ROMA	Risk of Ovarian Malignancy Algorithm
SEOC	serous epithelial ovarian cancer
sEV	small extracellular vesicle

## References

[1] Frisk N.L.S., Sørensen A.E., Pedersen O.B.V., Dalgaard L.T. (2023). Circulating microRNAs for Early Diagnosis of Ovarian Cancer: A Systematic Review and Meta-Analysis. Biomolecules.

[2] Cirillo P.D.R., Margiotti K., Fabiani M., Barros-Filho M.C., Sparacino D., Cima A., Longo S.A., Cupellaro M., Mesoraca A., Giorlandino C. (2021). Multi-Analytical Test Based on Serum miRNAs and Proteins Quantification for Ovarian Cancer Early Detection. PLoS ONE.

[3] Ren X., Zhang H., Cong H., Wang X., Ni H., Shen X., Ju S. (2018). Diagnostic Model of Serum miR-193a-5p, HE4 and CA-125 Improves the Diagnostic Efficacy of Epithelium Ovarian Cancer. Pathol. Oncol. Res..

[4] Webber J.W., Wollborn L., Mishra S., Vitonis A.F., Cramer D.W., Phan R.T., Pappas T.C., Stawiski K., Fendler W., Chowdhury D. (2024). Serum miRNA Improves the Accuracy of a Multivariate Index Assay for Triage of an Adnexal Mass. Gynecol. Oncol..

[5] Liang J., Yang X., Liu L., Qiao L., Peng P., Zhou J. (2020). Combined Measurement of miRNA-183, HE4, and CA-125 Increases Diagnostic Efficiency for Ovarian Cancer. Eur. J. Gynaecol. Oncol..

[6] Su Y.Y., Sun L., Guo Z.R., Li J.C., Bai T.T., Cai X.X., Li W.H., Zhu Y.F. (2019). Upregulated Expression of Serum Exosomal miR-375 and miR-1307 Enhance the Diagnostic Power of CA-125 for Ovarian Cancer. J. Ovarian Res..

[7] Frisk N.L.S., Jørgensen M.M., Bæk R., Atic A.I., Brodersen T.R., Ostrowski S.R., Larsen M.H., Posselt D., Høgdall E., Høgdall C. (2025). Characterization of Small Extracellular Vesicles from Ovarian Cancer Patients and Pre-Diagnostic Patient Samples: Evidence from the Danish Blood Donor Study. PLoS ONE.

[8] Yokoi A., Matsuzaki J., Yamamoto Y., Yoneoka Y., Takahashi K., Shimizu H., Uehara T., Ishikawa M., Ikeda S., Sonoda T. (2018). Integrated Extracellular microRNA Profiling for Ovarian Cancer Screening. Nat. Commun..

[9] Gahlawat A.W., Witte T., Haarhuis L., Schott S. (2022). A Novel Circulating miRNA Panel for Non-Invasive Ovarian Cancer Diagnosis and Prognosis. Br. J. Cancer.

[10] Oliveira D.N.P., Carlsen A.L., Heegaard N.H.H., Prahm K.P., Christensen I.J., Høgdall C.K., Høgdall E.V. (2019). Diagnostic Plasma miRNA-Profiles for Ovarian Cancer in Patients with Pelvic Mass. PLoS ONE.

[11] Niemira M., Erol A., Bielska A., Zeller A., Skwarska A., Chwialkowska K., Kuzmicki M., Szamatowicz J., Reszec J., Knapp P. (2023). Identification of Serum miR-1246 and miR-150-5p as Novel Diagnostic Biomarkers for High-Grade Serous Ovarian Cancer. Sci. Rep..

[12] Yoshida K., Yokoi A., Matsuzaki J., Kato T., Ochiya T., Kajiyama H., Yamamoto Y. (2021). Extracellular microRNA Profiling for Prognostic Prediction in Patients with High-grade Serous Ovarian Carcinoma. Cancer Sci..

[13] Kumar V., Gupta S., Chaurasia A., Sachan M. (2021). Evaluation of Diagnostic Potential of Epigenetically Deregulated MiRNAs in Epithelial Ovarian Cancer. Front. Oncol..

[14] Kandimalla R., Wang W., Yu F., Zhou N., Gao F., Spillman M., Moukova L., Slaby O., Salhia B., Zhou S. (2021). OCaMIR—A Noninvasive, Diagnostic Signature for Early-Stage Ovarian Cancer: A Multi-Cohort Retrospective and Prospective Study. Clin. Cancer Res..

[15] Witwer K.W., Buzás E.I., Bemis L.T., Bora A., Lässer C., Lötvall J., Nolte-‘t Hoen E.N., Piper M.G., Sivaraman S., Skog J. (2013). Standardization of Sample Collection, Isolation and Analysis Methods in Extracellular Vesicle Research. J. Extracell. Vesicle.

[16] Zhou Q., Zuo M.-Z., He Z., Li H.-R., Li W. (2018). Identification of Circulating microRNAs as Diagnostic Biomarkers for Ovarian Cancer: A Pooled Analysis of Individual Studies. Int. J. Biol. Markers.

[17] Huang Q., Chen J., Huang J. (2026). Exosomal microRNA Biomarkers in Ovarian Cancer Detection: Systematic Review and Meta-Analysis. Clin. Chim. Acta.

[18] Xu Y.-Z., Xi Q.-H., Ge W.-L., Zhang X.-Q. (2013). Identification of Serum MicroRNA-21 as a Biomarker for Early Detection and Prognosis in Human Epithelial Ovarian Cancer. Asian Pac. J. Cancer Prev..

[19] Zuberi M., Khan I., Mir R., Gandhi G., Ray P.C., Saxena A. (2016). Utility of Serum miR-125b as a Diagnostic and Prognostic Indicator and Its Alliance with a Panel of Tumor Suppressor Genes in Epithelial Ovarian Cancer. PLoS ONE.

[20] Zhou X., Liu M., Sun L., Cao Y., Tan S., Luo G., Liu T., Yao Y., Xiao W., Wan Z. (2023). Circulating Small Extracellular Vesicles MicroRNAs plus CA-125 for Treatment Stratification in Advanced Ovarian Cancer. J. Transl. Med..

[21] Günel T., Gumusoglu E., Dogan B., Ertem F.B., Hosseini M.K., Cevik N., Senol T., Topuz S., Aydinli K. (2018). Potential Biomarker of Circulating hsa-miR-1273g-3p Level for Detection of Recurrent Epithelial Ovarian Cancer. Arch. Gynecol. Obstet..

[22] Suryawanshi S., Vlad A.M., Lin H.-M., Mantia-Smaldone G., Laskey R., Lee M., Lin Y., Donnellan N., Klein-Patel M., Lee T. (2013). Plasma MicroRNAs as Novel Biomarkers for Endometriosis and Endometriosis-Associated Ovarian Cancer. Clin. Cancer Res..

[23] Shapira I., Oswald M., Lovecchio J., Khalili H., Menzin A., Whyte J., Dos Santos L., Liang S., Bhuiya T., Keogh M. (2014). Circulating biomarkers for detection of ovarian cancer and predicting cancer outcomes. Br. J. Cancer.

[24] Kumari P., Sharma I., Saha S.C., Srinivasan R., Bhardwaj P. (2022). Role of serum microRNAs as biomarkers for endometriosis, endometrioid carcinoma of ovary & endometrioid endometrial cancer. Indian J. Med. Res..

[25] Meng X., Müller V., Milde-Langosch K., Trillsch F., Pantel K., Schwarzenbach H. (2016). Circulating Cell-Free miR-373, miR-200a, miR-200b and miR-200c in Patients with Epithelial Ovarian Cancer. Adv. Exp. Med. Biol..

[26] Xiong J., He X., Xu Y., Zhang W., Fu F. (2021). MiR-200b is upregulated in plasma-derived exosomes and functions as an oncogene by promoting macrophage M2 polarization in ovarian cancer. J. Ovarian Res..

[27] Ruan L., Xie Y., Liu F., Chen X. (2018). Serum miR-1181 and miR-4314 associated with ovarian cancer: MiRNA microarray data analysis for a pilot study. Eur. J. Obstet. Gynecol. Reprod. Biol..

[28] Ji T., Zheng Z.G., Wang F.M., Xu L.J., Li L.F., Cheng Q.H., Guo J.F., Ding X.F. (2014). Differential microRNA Expression by Solexa Sequencing in the Sera of Ovarian Cancer Patients. Asian Pac. J. Cancer Prev..

[29] Tian L., Xu F., Lu Y., Deng Z., Gao Y., Yang J. (2025). Exploring a circulating circRNA and miRNA biomarker panel for early detection of ovarian cancer through multiple omics analysis. Sci. Rep..

[30] Sun L., Ke M., Yin M., Zeng Y., Ji Y., Hu Y., Fu S., Zhang C. (2024). Extracellular vesicle-encapsulated microRNA-296-3p from cancer-associated fibroblasts promotes ovarian cancer development through regulation of the PTEN/AKT and SOCS6/STAT3 pathways. Cancer Sci..

[31] Timofeeva A.V., Fedorov I.S., Asaturova A.V., Sannikova M.V., Tregubova A.V., Mayboroda O.A., Khabas G.N., Frankevich V.E., Sukhikh G.T. (2023). Blood Plasma Small Non-Coding RNAs as Diagnostic Molecules for the Progesterone-Receptor-Negative Phenotype of Serous Ovarian Tumors. Int. J. Mol. Sci..

[32] Timofeeva A.V., Asaturova A.V., Sannikova M.V., Khabas G.N., Chagovets V.V., Fedorov I.S., Frankevich V.E., Sukhikh G.T. (2022). Search for New Participants in the Pathogenesis of High-Grade Serous Ovarian Cancer with the Potential to Be Used as Diagnostic Molecules. Life.

[33] Gumusoglu E., Gunel T., Hosseini M.K., Seymen N., Senol T., Sezerman U., Topuz S., Aydinli K. (2021). The importance of dysregulated miRNAs on ovarian cysts and epithelial ovarian cancer. Eur. J. Gynaecol. Oncol..

[34] An Y., Zhang J., Cheng X., Li B., Tian Y., Zhang X., Zhao F. (2020). miR-454 suppresses the proliferation and invasion of ovarian cancer by targeting E2F6. Cancer Cell Int..

[35] Gunel T., Dogan B., Gumusoglu E., Hosseini M.K., Topuz S., Aydinli K. (2019). Regulation of HMGA2 and KRAS genes in epithelial ovarian cancer by miRNA hsa-let-7d-3p. J. Cancer Res. Ther..

[36] Kumar V., Gupta S., Varma K., Chaurasia A., Sachan M. (2022). Diagnostic Performance of microRNA-34a, Let-7f and microRNA-31 in Epithelial Ovarian Cancer Prediction. J. Gynecol. Oncol..

[37] Wang W., Wu L., Li C., Zhou X., Liu P., Jia X., Chen Y., Zhu W. (2019). Five Serum microRNAs for Detection and Predicting of Ovarian Cancer. Eur. J. Obstet. Gynecol. Reprod. Biol. X.

[38] Wang W., Yin Y., Shan X., Zhou X., Liu P., Cao Q., Zhu D., Zhang J., Zhu W. (2019). The Value of Plasma-Based MicroRNAs as Diagnostic Biomarkers for Ovarian Cancer. Am. J. Med. Sci..

[39] Meng X., Müller V., Milde-Langosch K., Trillsch F., Pantel K., Schwarzenbach H. (2016). Diagnostic and Prognostic Relevance of Circulating Exosomal miR-373, miR-200a, miR-200b and miR-200c in Patients with Epithelial Ovarian Cancer. Oncotarget.

[40] Meng X., Joosse S.A., Müller V., Trillsch F., Milde-Langosch K., Mahner S., Geffken M., Pantel K., Schwarzenbach H. (2015). Diagnostic and Prognostic Potential of Serum miR-7, miR-16, miR-25, miR-93, miR-182, miR-376a and miR-429 in Ovarian Cancer Patients. Br. J. Cancer.

[41] Zuberi M., Mir R., Khan I., Javid J., Guru S.A., Bhat M., Sumi M.P., Ahmad I., Masroor M., Yadav P. (2019). The Promising Signatures of Circulating microRNA-145 in Epithelial Ovarian Cancer Patients. MIRNA.

[42] Zuberi M., Khan I., Gandhi G., Ray P.C., Saxena A. (2016). The Conglomeration of Diagnostic, Prognostic and Therapeutic Potential of Serum miR-199a and Its Association with Clinicopathological Features in Epithelial Ovarian Cancer. Tumor Biol..

[43] Fukagawa S., Miyata K., Yotsumoto F., Kiyoshima C., Nam S.O., Anan H., Katsuda T., Miyahara D., Murata M., Yagi H. (2017). MicroRNA-135a-3p as a Promising Biomarker and Nucleic Acid Therapeutic Agent for Ovarian Cancer. Cancer Sci..

[44] Márton É., Lukács J., Penyige A., Janka E., Hegedüs L., Soltész B., Méhes G., Póka R., Nagy B., Szilágyi M. (2019). Circulating Epithelial-Mesenchymal Transition-Associated miRNAs Are Promising Biomarkers in Ovarian Cancer. J. Biotechnol..

[45] Penyige A., Márton É., Soltész B., Szilágyi-Bónizs M., Póka R., Lukács J., Széles L., Nagy B. (2019). Circulating miRNA Profiling in Plasma Samples of Ovarian Cancer Patients. Int. J. Mol. Sci..

[46] Gumusoglu-Acar E., Gunel T., Hosseini M., Dogan B., Tekarslan E., Gurdamar B., Cevik N., Sezerman U., Topuz S., Aydinli K. (2023). Metabolic Pathways of Potential miRNA Biomarkers Derived from Liquid Biopsy in Epithelial Ovarian Cancer. Oncol. Lett..

[47] Zhu Z., Chen Z., Wang M., Zhang M., Chen Y., Yang X., Zhou C., Liu Y., Hong L., Zhang L. (2022). Detection of Plasma Exosomal miRNA-205 as a Biomarker for Early Diagnosis and an Adjuvant Indicator of Ovarian Cancer Staging. J. Ovarian Res..

[48] Todeschini P., Salviato E., Paracchini L., Ferracin M., Petrillo M., Zanotti L., Tognon G., Gambino A., Calura E., Caratti G. (2017). Circulating miRNA Landscape Identifies miR-1246 as Promising Diagnostic Biomarker in High-Grade Serous Ovarian Carcinoma: A Validation across Two Independent Cohorts. Cancer Lett..

[49] Choi P.-W., Bahrampour A., Ng S.-K., Liu S.K., Qiu W., Xie F., Kuo W.P., Kwong J., Hales K.H., Hales D.B. (2020). Characterization of miR-200 Family Members as Blood Biomarkers for Human and Laying Hen Ovarian Cancer. Sci. Rep..

[50] Yokoi A., Yoshioka Y., Hirakawa A., Yamamoto Y., Ishikawa M., Ikeda S., Kato T., Niimi K., Kajiyama H., Kikkawa F. (2017). A Combination of Circulating miRNAs for the Early Detection of Ovarian Cancer. Oncotarget.

[51] Gockley A., Pagacz K., Fiascone S., Stawiski K., Holub N., Hasselblatt K., Cramer D.W., Fendler W., Chowdhury D., Elias K.M. (2022). A Translational Model to Improve Early Detection of Epithelial Ovarian Cancers. Front. Oncol..

[52] Elias K.M., Fendler W., Stawiski K., Fiascone S.J., Vitonis A.F., Berkowitz R.S., Frendl G., Konstantinopoulos P., Crum C.P., Kedzierska M. (2017). Diagnostic Potential for a Serum miRNA Neural Network for Detection of Ovarian Cancer. eLife.

[53] Wang W., Jo H., Park S., Kim H., Kim S.I., Han Y., Lee J., Seol A., Kim J., Lee M. (2022). Integrated Analysis of Ascites and Plasma Extracellular Vesicles Identifies a miRNA-Based Diagnostic Signature in Ovarian Cancer. Cancer Lett..

[54] Hong G., Luo F., Chen Z., Ma L., Lin G., Wu T., Li N., Cai H., Hu T., Zhong H. (2022). Predict Ovarian Cancer by Pairing Serum miRNAs: Construct of Single Sample Classifiers. Front. Med..

[55] Hamidi F., Gilani N., Belaghi R.A., Sarbakhsh P., Edgünlü T., Santaguida P. (2021). Exploration of Potential miRNA Biomarkers and Prediction for Ovarian Cancer Using Artificial Intelligence. Front. Genet..

[56] Yang L., Yang Z., Liu Z., Qi N., Tao L. (2024). Diagnostic Value of Plasma-Derived Exosomal miR-223 for Epithelial Ovarian Cancer. BMC Women’s Health.

[57] Wiczling P., Daghir-Wojtkowiak E., Kaliszan R., Markuszewski M.J., Limon J., Koczkowska M., Stukan M., Kuźniacka A., Ratajska M. (2019). Bayesian Multilevel Model of Micro RNA Levels in Ovarian-Cancer and Healthy Subjects. PLoS ONE.

[58] Ali F.T., Soliman R.M., Hassan N.S., Ibrahim A.M., El-Gizawy M.M., Mandoh A.A.Y., Ibrahim E.A. (2022). Sensitivity and Specificity of microRNA-204, CA-125, and CA19.9 as Biomarkers for Diagnosis of Ovarian Cancer. PLoS ONE.

[59] Kan C.W., Hahn M.A., Gard G.B., Maidens J., Huh J.Y., Marsh D.J., Howell V.M. (2012). Elevated Levels of Circulating microRNA-200 Family Members Correlate with Serous Epithelial Ovarian Cancer. BMC Cancer.

[60] Khan K., Zafar S., Badshah Y., Ashraf N.M., Rafiq M., Danish L., Shabbir M., Trembley J.H., Afsar T., Almajwal A. (2023). Cross Talk of Tumor Protein D52 (TPD52) with KLF9, PKCε, and MicroRNA 223 in Ovarian Cancer. J. Ovarian Res..

[61] Langhe R., Norris L., Saadeh F.A., Blackshields G., Varley R., Harrison A., Gleeson N., Spillane C., Martin C., O’Donnell D.M. (2015). A Novel Serum microRNA Panel to Discriminate Benign from Malignant Ovarian Disease. Cancer Lett..

[62] Liu J., Yoo J., Ho J.Y., Jung Y., Lee S., Hur S.Y., Choi Y.J. (2021). Plasma-Derived Exosomal miR-4732-5p Is a Promising Noninvasive Diagnostic Biomarker for Epithelial Ovarian Cancer. J. Ovarian Res..

[63] Paliwal N., Vashist M., Chauhan M. (2020). Evaluation of miR-22 and miR-21 as Diagnostic Biomarkers in Patients with Epithelial Ovarian Cancer. 3 Biotech.

[64] Ritter A., Hirschfeld M., Berner K., Jaeger M., Grundner-Culemann F., Schlosser P., Asberger J., Weiss D., Noethling C., Mayer S. (2020). Discovery of Potential Serum and Urine-Based microRNA as Minimally-Invasive Biomarkers for Breast and Gynecological Cancer. Cancer Biomark..

[65] Stawiski K., Fortner R.T., Pestarino L., Umu S.U., Kaaks R., Rounge T.B., Elias K.M., Fendler W., Langseth H. (2024). Validation of miRNA Signatures for Ovarian Cancer Earlier Detection in the Pre-Diagnosis Setting Using Machine Learning Approaches. Front. Oncol..

[66] Takamizawa S., Kojima J., Umezu T., Kuroda M., Hayashi S., Maruta T., Okamoto A., Nishi H. (2023). miR-146a-5p and miR-191-5p as Novel Diagnostic Marker Candidates for Ovarian Clear Cell Carcinoma. Mol. Clin. Oncol..

[67] Yoshimura A., Sawada K., Nakamura K., Kinose Y., Nakatsuka E., Kobayashi M., Miyamoto M., Ishida K., Matsumoto Y., Kodama M. (2018). Exosomal miR-99a-5p Is Elevated in Sera of Ovarian Cancer Patients and Promotes Cancer Cell Invasion by Increasing Fibronectin and Vitronectin Expression in Neighboring Peritoneal Mesothelial Cells. BMC Cancer.

[68] Zhang A., Hu H. (2022). A Novel Blood-Based microRNA Diagnostic Model with High Accuracy for Multi-Cancer Early Detection. Cancers.

[69] Chen H., Zhang L., Zhang L., Du J., Wang H., Wang B. (2016). MicroRNA-183 Correlates Cancer Prognosis, Regulates Cancer Proliferation and Bufalin Sensitivity in Epithelial Ovarian Cancer. Am. J. Transl. Res..

[70] Gong L., Wang C., Gao Y., Wang J. (2016). Decreased Expression of microRNA-148a Predicts Poor Prognosis in Ovarian Cancer and Associates with Tumor Growth and Metastasis. Biomed. Pharmacother..

[71] Zhong S., Wu W., Liang Y., Wu Z. (2025). Prognostic Significance and Therapeutic Outcome Prediction through Serum miR-21 and miR-145 in Ovarian Cancer. An. Acad. Bras. Ciênc..

[72] Pal R., Choudhury T., Ghosh M., Vernakar M., Nath P., Nasare V.D. (2024). A Signature of Circulating miRNAs Predicts the Prognosis and Therapeutic Outcome of Taxane/Platinum Regimen in Advanced Ovarian Carcinoma Patients. Clin. Transl. Oncol..

[73] Kapetanakis N.-I., Uzan C., Jimenez-Pailhes A.-S., Gouy S., Bentivegna E., Morice P., Caron O., Gourzones-Dmitriev C., Le Teuff G., Busson P. (2015). Plasma miR-200b in Ovarian Carcinoma Patients: Distinct Pattern of Pre/Post-Treatment Variation Compared to CA-125 and Potential for Prediction of Progression-Free Survival. Oncotarget.

[74] Chao A., Lai C.-H., Chen H.-C., Lin C.-Y., Tsai C.-L., Tang Y.-H., Huang H.-J., Lin C.-T., Chen M.-Y., Huang K.-G. (2014). Serum microRNAs in Clear Cell Carcinoma of the Ovary. Taiwan. J. Obstet. Gynecol..

[75] Robelin P., Tod M., Colomban O., Lachuer J., Ray-Coquard I., Rauglaudre G.D., Joly F., Chevalier-Place A., Combe P., Lortholary A. (2020). Comparative Analysis of Predictive Values of the Kinetics of 11 Circulating miRNAs and of CA-125 in Ovarian Cancer during First Line Treatment (a GINECO Study). Gynecol. Oncol..

[76] Benson E.A., Skaar T.C., Liu Y., Nephew K.P., Matei D. (2015). Carboplatin with Decitabine Therapy, in Recurrent Platinum Resistant Ovarian Cancer, Alters Circulating miRNAs Concentrations: A Pilot Study. PLoS ONE.

[77] Halvorsen A.R., Kristensen G., Embleton A., Adusei C., Barretina-Ginesta M.P., Beale P., Helland Å. (2017). Evaluation of Prognostic and Predictive Significance of Circulating MicroRNAs in Ovarian Cancer Patients. Dis. Markers.

[78] Uehara T., Matsuzaki J., Yoshida H., Ogawa Y., Miura J., Fujimiya H., Yamamoto Y., Kawauchi J., Takizawa S., Yonemori K. (2024). Potential Utility of Pretreatment Serum miRNAs for Optimal Treatment Selection in Advanced High-Grade Serous Ovarian Cancer. Jpn. J. Clin. Oncol..

[79] Zhao J., Dong X., Liu Q.-C., Lu Q. (2018). Expression of Plasma miR-106a in Epithelial Ovarian Cancer and Its Diagnostic and Prognostic Significance. Eur. J. Gynaecol. Oncol..

[80] Helmy D., Mossallam G., Radwan N., Kamal A., Attia I. (2025). Value of miR200b and Its Combination with Other Biochemical Markers in the Diagnosis of Epithelial Ovarian Cancer. Mol. Biol. Rep..

[81] Zhang W., Su X., Li S., Liu Z., Wang Q., Zeng H. (2020). Low Serum Exosomal miR-484 Expression Predicts Unfavorable Prognosis in Ovarian Cancer. Cancer Biomark..

[82] Shah J.S., Gard G.B., Yang J., Maidens J., Valmadre S., Soon P.S., Marsh D.J. (2018). Combining Serum microRNA and CA-125 as Prognostic Indicators of Preoperative Surgical Outcome in Women with High-Grade Serous Ovarian Cancer. Gynecol. Oncol..

[83] Li L., Zhang F., Zhang J., Shi X., Wu H., Chao X., Ma S., Lang J., Wu M., Zhang D. (2023). Identifying Serum Small Extracellular Vesicle MicroRNA as a Noninvasive Diagnostic and Prognostic Biomarker for Ovarian Cancer. ACS Nano.

[84] Hong F., Li Y., Xu Y., Zhu L. (2013). Prognostic Significance of Serum microRNA-221 Expression in Human Epithelial Ovarian Cancer. J. Int. Med. Res..

[85] Chen Z., Guo X., Sun S., Lu C., Wang L. (2020). Serum miR-125b Levels Associated with Epithelial Ovarian Cancer (EOC) Development and Treatment Responses. Bioengineered.

[86] Zhu T., Gao W., Chen X., Zhang Y., Wu M., Zhang P., Wang S. (2017). A Pilot Study of Circulating MicroRNA-125b as a Diagnostic and Prognostic Biomarker for Epithelial Ovarian Cancer. Int. J. Gynecol. Cancer.

[87] Song K.-W., Zhang Q.-G., Tan W.-B., Fang Y.-N. (2020). Diagnostic Significance of Serum miR-26b and miR-21 Expressions in Ovarian Cancer and Their Associations with Clinicopathological Characteristics and Prognosis of Patients. Eur. Rev. Med. Pharmacol. Sci..

[88] Maeda K., Sasaki H., Ueda S., Miyamoto S., Terada S., Konishi H., Kogata Y., Ashihara K., Fujiwara S., Tanaka Y. (2020). Serum Exosomal microRNA-34a as a Potential Biomarker in Epithelial Ovarian Cancer. J. Ovarian Res..

[89] Gao Y., Wu J. (2015). MicroRNA-200c and microRNA-141 as Potential Diagnostic and Prognostic Biomarkers for Ovarian Cancer. Tumor Biol..

[90] Guo F., Tian J., Lin Y., Jin Y., Wang L., Cui M. (2013). Serum microRNA-92 Expression in Patients with Ovarian Epithelial Carcinoma. J. Int. Med. Res..

[91] Habel A., Nassar F., Itani M., Bouaziz H., Hadj-Ahmed M., Msheik Z., Stayoussef M., Nasr R., Yacoubi-Loueslati B. (2023). Mir-21 and Mir-125b as Theranostic Biomarkers for Epithelial Ovarian Cancer in Tunisian Women. Afr. Health Sci..

[92] Minareci Y., Ak N., Sozen H., Tosun O.A., Kucukgergin C., Aydin F., Bingul İ., Salihoglu M.Y., Topuz S. (2024). The Evaluation of miR-1181 and miR-4314 as Serum microRNA Biomarkers for Epithelial Ovarian Cancer Diagnosis and Prognosis. Mol. Biol. Rep..

[93] Ranmale S., Kumar P., Tongaonkar H., Mehta S., Maniar V., Mania-Pramanik J. (2025). Chemotherapy-Induced Alterations in miRNA Expression and Their Prognostic Implications in Ovarian Cancer. Front. Oncol..

[94] Yunusova N., Dzhugashvili E., Yalovaya A., Kolomiets L., Shefer A., Grigor’eva A., Tupikin A., Kondakova I., Tamkovich S. (2022). Comparative Analysis of Tumor-Associated microRNAs and Tetraspanines from Exosomes of Plasma and Ascitic Fluids of Ovarian Cancer Patients. Int. J. Mol. Sci..

[95] Zheng H., Zhang L., Zhao Y., Yang D., Song F., Wen Y., Hao Q., Hu Z., Zhang W., Chen K. (2013). Plasma miRNAs as Diagnostic and Prognostic Biomarkers for Ovarian Cancer. PLoS ONE.

[96] Suri A., Perumal V., Ammalli P., Suryan V., Bansal S.K. (2021). Diagnostic Measures Comparison for Ovarian Malignancy Risk in Epithelial Ovarian Cancer Patients: A Meta-Analysis. Sci. Rep..

[97] Wolff R.F., Moons K.G.M., Riley R.D., Whiting P.F., Westwood M., Collins G.S., Reitsma J.B., Kleijnen J., Mallett S., PROBAST Group (2019). PROBAST: A Tool to Assess the Risk of Bias and Applicability of Prediction Model Studies. Ann. Intern. Med..

[98] Whiting P.F., Rutjes A.W.S., Westwood M.E., Mallett S., Deeks J.J., Reitsma J.B., Leeflang M.M.G., Sterne J.A.C., Bossuyt P.M.M., QUADAS-2 Group (2011). QUADAS-2: A Revised Tool for the Quality Assessment of Diagnostic Accuracy Studies. Ann. Intern. Med..

[99] Salomon C., Lai A., Guanzon D., Sharma S., Scholz-Romero K., Razo M., Barnard A., Choolani M., Palma C., Khanabdali R. (2025). Early detection of ovarian cancer: An accurate high-throughput extracellular vesicle test. J. Clin. Oncol..

[100] Page M.J., McKenzie J.E., Bossuyt P.M., Boutron I., Hoffmann T.C., Mulrow C.D., Shamseer L., Tetzlaff J.M., Akl E.A., Brennan S.E. (2021). The PRISMA 2020 Statement: An Updated Guideline for Reporting Systematic Reviews. BMJ.

[101] Ouzzani M., Hammady H., Fedorowicz Z., Elmagarmid A. (2016). Rayyan—A Web and Mobile App for Systematic Reviews. Syst. Rev..

[102] Hayden J.A., van der Windt D.A., Cartwright J.L., Cote P., Bombardier C. (2013). Assessing Bias in Studies of Prognostic Factors. Ann. Intern. Med..

