# Supplementary material for: Circulating miRNAs and Multivariate Biomarker Signatures in Ovarian Cancer: A Systematic Review
Source: Int J Mol Sci. 2026 Oct 2;27(19):8823. doi: 10.3390/ijms27198823 (PMC13664815; doi:10.3390/ijms27198823)
Supplement: Supplementary file 1 [file ijms-27-08823-s001.zip › Supplementary_Table_S2_Complete_Electronic_Search_Strategies.pdf]

Supplementary Table S2. Complete electronic search strategies used to identify studies evaluating circulating microRNAs as diagnostic or prognostic biomarkers in ovarian cancer

Database	Complete electronic search strategy	Limits applied	Date searched	Records retrieved
**MEDLINE via PubMed**	("Ovarian Neoplasms"[Mesh] OR "ovarian neoplasm*"[Title/Abstract] OR "ovarian cancer*"[Title/Abstract] OR "ovarian carcinoma*"[Title/Abstract] OR "ovarian tumor*"[Title/Abstract] OR "ovarian tumour*"[Title/Abstract] OR "epithelial ovarian cancer"[Title/Abstract] OR EOC[Title/Abstract] OR "high-grade serous ovarian cancer"[Title/Abstract] OR HGSOC[Title/Abstract]) AND ("MicroRNAs"[Mesh] OR microRNA*[Title/Abstract] OR miRNA*[Title/Abstract] OR miR[Title/Abstract] OR "miR-"[Title/Abstract] OR "micro-RNA*"[Title/Abstract]) AND ("Biomarkers, Tumor"[Mesh] OR "Liquid Biopsy"[Mesh] OR "blood biomarker*"[Title/Abstract] OR "circulating biomarker*"[Title/Abstract] OR "liquid biopsy"[Title/Abstract] OR circulating[Title/Abstract] OR blood[Title/Abstract] OR serum[Title/Abstract] OR plasma[Title/Abstract] OR "whole blood"[Title/Abstract] OR cell-free[Title/Abstract] OR exosom*[Title/Abstract] OR "extracellular vesicle*"[Title/Abstract]) AND ("Diagnosis"[Mesh] OR "Prognosis"[Mesh] OR "Early Detection of Cancer"[Mesh] OR diagnos*[Title/Abstract] OR detect*[Title/Abstract] OR screen*[Title/Abstract] OR prognos*[Title/Abstract] OR survival[Title/Abstract] OR recurren*[Title/Abstract] OR progression[Title/Abstract] OR monitor*[Title/Abstract] OR predict*[Title/Abstract] OR sensitivity[Title/Abstract] OR specificity[Title/Abstract] OR "receiver operating characteristic"[Title/Abstract] OR	English language; no publication-date restriction; database searched from inception	30 May 2026	**311**

Database	Complete electronic search strategy	Limits applied	Date searched	Records retrieved
	ROC[Title/Abstract] OR AUC[Title/Abstract])			
**Scopus**	TITLE-ABS-KEY(("ovarian neoplasm*" OR "ovarian cancer*" OR "ovarian carcinoma*" OR "ovarian tumor*" OR "ovarian tumour*" OR "epithelial ovarian cancer" OR EOC OR "high-grade serous ovarian cancer" OR HGSOC) AND (microRNA* OR miRNA* OR miR OR "miR-" OR "micro-RNA*") AND ("tumor biomarker*" OR "tumour biomarker*" OR "blood biomarker*" OR "circulating biomarker*" OR "liquid biopsy" OR circulating OR blood OR serum OR plasma OR "whole blood" OR "cell-free" OR exosom* OR "extracellular vesicle*") AND (diagnos* OR detect* OR screen* OR prognos* OR survival OR recurren* OR progression OR monitor* OR predict* OR sensitivity OR specificity OR "receiver operating characteristic" OR ROC OR AUC))	English language; no publication-date restriction; database searched from inception	30 May 2026	**376**
**Cochrane Central Register of Controlled Trials — CENTRAL**	#1 MeSH descriptor: [Ovarian Neoplasms] explode all trees<br>#2 ("ovarian neoplasm*" OR "ovarian cancer*" OR "ovarian carcinoma*" OR "ovarian tumor*" OR "ovarian tumour*" OR "epithelial ovarian cancer" OR EOC OR "high-grade serous ovarian cancer" OR HGSOC):ti,ab,kw<br>#3 #1 OR #2<br>#4 MeSH descriptor: [MicroRNAs] explode all trees<br>#5 (microRNA* OR miRNA* OR miR OR "miR-" OR "micro-RNA*"):ti,ab,kw<br>#6 #4 OR #5<br>#7 MeSH descriptor: [Biomarkers, Tumor] explode all trees<br>#8 MeSH descriptor: [Liquid Biopsy] explode all trees<br>#9 ("tumor biomarker*" OR "tumour biomarker*" OR "blood biomarker*" OR "circulating biomarker*" OR "liquid biopsy" OR circulating OR blood OR serum OR plasma OR "whole blood" OR "cell-free" OR exosom* OR "extracellular vesicle*"):ti,ab,kw<br>#10 #7 OR #8 OR #9<br>#11 MeSH descriptor: [Diagnosis] explode all trees<br>#12 MeSH descriptor: [Prognosis] explode all trees	No publication-date restriction; database searched from inception. English-language eligibility was applied during screening if CENTRAL did not permit an equivalent search filter.	30 May 2026	**10**

Database	Complete electronic search strategy	Limits applied	Date searched	Records retrieved
	#13 MeSH descriptor: [Early Detection of Cancer] explode all trees<br>#14 (diagnos* OR detect* OR screen* OR prognos* OR survival OR recurren* OR progression OR monitor* OR predict* OR sensitivity OR specificity OR "receiver operating characteristic" OR ROC OR AUC):ti,ab,kw<br>#15 #11 OR #12 OR #13 OR #14<br>#16 #3 AND #6 AND #10 AND #15			

Abbreviations: AUC, area under the receiver operating characteristic curve; CENTRAL, Cochrane Central Register of Controlled Trials; EOC, epithelial ovarian cancer; HGSOC, high-grade serous ovarian cancer; MeSH, Medical Subject Headings; miRNA, microRNA; ROC, receiver operating characteristic.

Additional search methods: The reference lists of all included studies and relevant review articles were manually screened to identify additional eligible publications.

Note: Searches covered each database from inception to 30 May 2026. Results were exported and deduplicated before title and abstract screening in Rayyan. The electronic strategies are reported in full to satisfy PRISMA 2020 Item 7 and support reproducibility.
