# Supplementary material for: Circulating miRNAs and Multivariate Biomarker Signatures in Ovarian Cancer: A Systematic Review
Source: Int J Mol Sci. 2026 Oct 2;27(19):8823. doi: 10.3390/ijms27198823 (PMC13664815; doi:10.3390/ijms27198823)
Supplement: Supplementary file 1 [file ijms-27-08823-s001.zip › Supplementary_Table_S3_Evidence_Map_Classifications.pdf]

Supplementary Table S3 Evidence mapping for Figure 4

Circulating miRNAs and Multivariate Biomarker Signatures in Ovarian Cancer: A Systematic Review

This descriptive mapping covers all 91 included publications. Criteria were operationalized retrospectively during manuscript revision. Each publication is counted once per applicable cell; a publication can contribute to multiple cells. Counts describe research coverage, not risk of bias, certainty, effect magnitude or numbers of independent cohorts. Reference numbers correspond to the manuscript; reference [96] is not an included study.

Classification and counting rules

Assignments require reported human circulating-miRNA analyses. Negative findings are retained. Tissue-only, cell-line and animal findings do not establish clinical coverage. Measuring several miRNAs does not establish a combined model. Assay confirmation, internal validation, external evaluation and model refitting are distinguished in the study notes. Validation is described separately rather than treated as a clinical application.

Code and category	Operational definition
I — Individual or total circulating miRNA	Individual-marker results or total circulating miRNA measurement. Multiple separately analysed markers remain individual-marker evidence.
M — MultimiRNA combinations	An explicit combined miRNA score, classifier or joint model without protein markers.
C — miRNA plus protein combinations	Joint miRNA/protein analysis, including integrated diagnostic models or joint survival strata. Comparison with a protein alone does not qualify.
V — Isolated EV or exosomal miRNAs	Measurements in isolated human extracellular vesicles or exosomes. Total serum/plasma tests supported only by cell-derived EV experiments do not qualify.
A — Algorithmic or penalized models	Neural networks, discriminant classifiers, voting classifiers, penalized models or supervised feature-selection modelling workflows. Ordinary logistic/Cox regression or Bayesian association modelling alone does not qualify.
B — Prediagnostic sampling	Human samples obtained before clinical diagnosis. Includes the explicitly identified case-only time-to-diagnosis analysis [65]; this is not cancer-versus-control screening accuracy.

Clinical application definitions

Code and application	Operational definition
D — Diagnostic investigation	Human diagnostic comparisons, including expression-only case-control analyses, or prediagnostic investigation. D does not imply validated diagnostic accuracy. The case-only study [65] is distinguished in its note.
E — Early-stage diagnostic performance	Explicit performance for FIGO I or I–II cancer versus noncancer comparators. Merely including early-stage cases, stage correlations or early-versus-advanced discrimination is insufficient.
P — Prognostic outcomes	Observed survival, progression, event-free survival, future recurrence or follow-up vital status. Stage/metastasis associations alone are insufficient.
R — Serial recurrence monitoring	Serial circulating-marker measurements used to detect recurrence. Recurrence-free survival or recurrence risk alone belongs to P.
T — Treatment-related clinical analyses	Clinical response, treatment selection, surgical-outcome prediction or pre/post-treatment marker changes. Cell/mouse drug experiments and residual tumour as a prognostic covariate alone are insufficient.

A zero means that no eligible result was assigned under these definitions, not that an application is ineffective. Categories overlap conceptually: an EV-based penalized multimiRNA classifier can appear in V, A and M. Cell counts must not be added to estimate the total number of studies. Early-stage case-control discrimination is not evidence of population screening benefit.

Cell counts and supporting references

Category	Application	n	Manuscript references
Individual or total circulating miRNA	Diagnostic investigation	72	[2], [3], [5], [6], [7], [9], [10], [11], [13], [18], [19], [20], [21], [22], [23], [24], [25], [26], [27], [28], [29], [30], [32], [33], [34], [35], [36], [37], [38], [39], [40], [41], [42], [43], [44], [45], [46], [47], [48], [49], [50], [53], [56], [57], [58], [59], [60], [61], [62], [63], [64], [66], [67], [70], [72], [73], [79], [80], [81], [82], [83], [84], [85], [86], [87], [89], [90], [91], [92], [93], [94], [95]
Individual or total circulating miRNA	Early-stage diagnostic performance	7	[13], [29], [36], [56], [58], [67], [92]
Individual or total circulating miRNA	Prognostic outcomes	32	[9], [12], [18], [19], [23], [39], [40], [41], [42], [43], [53], [69], [70], [71], [72], [73], [75], [76], [77], [78], [79], [80], [81], [84], [85], [86], [87], [88], [89], [92], [93], [95]
Individual or total circulating miRNA	Serial recurrence monitoring	1	[74]

Category	Application	n	Manuscript references
Individual or total circulating miRNA	Treatment-related clinical analyses	19	[20], [23], [30], [31], [32], [67], [71], [72], [73], [74], [75], [76], [77], [78], [82], [83], [85], [91], [93]
MultimiRNA combinations	Diagnostic investigation	33	[2], [4], [6], [8], [9], [10], [11], [13], [14], [22], [25], [36], [37], [38], [39], [40], [48], [49], [50], [51], [52], [53], [54], [55], [57], [59], [65], [68], [83], [87], [92], [93], [95]
MultimiRNA combinations	Early-stage diagnostic performance	15	[2], [8], [9], [11], [13], [14], [36], [50], [51], [52], [53], [54], [83], [92], [95]
MultimiRNA combinations	Prognostic outcomes	4	[12], [71], [78], [83]
MultimiRNA combinations	Serial recurrence monitoring	0	None assigned
MultimiRNA combinations	Treatment-related clinical analyses	4	[20], [32], [78], [83]
miRNA plus protein combinations	Diagnostic investigation	17	[2], [3], [4], [5], [6], [9], [10], [20], [47], [50], [56], [58], [67], [80], [81], [82], [85]
miRNA plus protein combinations	Early-stage diagnostic performance	6	[2], [4], [9], [56], [58], [67]
miRNA plus protein combinations	Prognostic outcomes	1	[81]
miRNA plus protein combinations	Serial recurrence monitoring	0	None assigned
miRNA plus protein combinations	Treatment-related clinical analyses	2	[20], [82]
Isolated EV or exosomal miRNAs	Diagnostic investigation	14	[6], [7], [20], [26], [37], [38], [39], [47], [53], [56], [62], [81], [83], [94]
Isolated EV or exosomal miRNAs	Early-stage diagnostic performance	2	[56], [83]
Isolated EV or exosomal miRNAs	Prognostic outcomes	5	[39], [53], [81], [83], [88]
Isolated EV or exosomal miRNAs	Serial recurrence monitoring	0	None assigned
Isolated EV or exosomal miRNAs	Treatment-related clinical analyses	2	[20], [83]
Algorithmic or penalized models	Diagnostic investigation	13	[4], [8], [11], [20], [22], [49], [51], [52], [54], [55], [65], [68], [83]
Algorithmic or penalized models	Early-stage diagnostic performance	7	[4], [8], [11], [51], [52], [54], [83]
Algorithmic or penalized models	Prognostic outcomes	2	[78], [83]
Algorithmic or penalized models	Serial recurrence monitoring	0	None assigned
Algorithmic or penalized models	Treatment-related clinical analyses	3	[20], [78], [83]

Category	Application	n	Manuscript references
Prediagnostic sampling	Diagnostic investigation	2	[7], [65]
Prediagnostic sampling	Early-stage diagnostic performance	0	None assigned
Prediagnostic sampling	Prognostic outcomes	0	None assigned
Prediagnostic sampling	Serial recurrence monitoring	0	None assigned
Prediagnostic sampling	Treatment-related clinical analyses	0	None assigned

Study-level assignments and interpretive notes

Codes identify the exact figure cells. For example, M: D, E denotes a multimiRNA combination with diagnostic and early-stage diagnostic results. Source findings and validation qualifications are shown separately; a validation label is not a risk-of-bias judgement.

Study and reference	Assigned cells	Source findings supporting assignment	Validation and interpretation
Oliveira et al. (2019)[10]	I: D; M: D; C: D	Plasma individual miRNAs, two-miRNA classifier and addition of CA125; diagnostic ROC analyses. Early-stage performance explicitly not evaluated separately.	Separate discovery/validation groups from the same study population; validation restricted to late-stage disease. Primary platform: qRT-PCR. Clinical blood matrix: Plasma.
Cirillo et al. (2021) [2]	I: D; M: D, E; C: D, E	Results on individual miRNAs, miRNA classifier and miRNA plus CA125/HE4 classifier, including early-stage ROC results.	Held-out clinical sample set; cross-study evaluation of the miRNA-only classifier. Do not transfer that evaluation to the protein-inclusive model. Primary platform: Digital PCR. Clinical blood matrix: Serum.
Frisk et al. (2025) [7]	I: D; V: D; B: D	EV miRNA measurements in diagnostic and prediagnostic samples; the miRNAs did not distinguish the groups reliably. Negative findings count as coverage.	Discovery and technical replication/validation; not an externally validated miRNA screening test. Primary platform: qRT-PCR. Clinical blood matrix: Plasma.
Yokoi et al. (2018) [8]	M: D, E; A: D, E	Serum multimiRNA discriminant models; stage-specific diagnostic sensitivity assessed.	Discovery/validation split within the assembled study data; an independent split is not automatically external validation. Primary platform: Microarray. Clinical blood matrix: Serum.
Gahlawat et al. (2022)[9]	I: D, P; M: D, E; C: D, E	Total circulating miRNA level associated with survival; seven-miRNA diagnostic panel, with and without CA125, including early-stage ROC analyses.	An external published dataset was also used in marker discovery and re-evaluation. The CA125-inclusive model was not externally validated; survival findings concern total miRNA level. Primary platform: NGS. Clinical blood matrix: Plasma.
Niemira et al. (2023)[11]	I: D; M: D, E; A: D, E	Individual serum markers and feature-selected logistic models; stage-specific models examined in public serum data.	Random internal split and cross-validation, assay verification and public-data re-modelling. Stage-specific models were fitted in public data, not a prospective screening validation. Primary platform: NanoString. Clinical blood matrix: Serum. NanoString was the primary serum-profiling platform; tissue RNA-seq was ancillary.

Study and reference	Assigned cells	Source findings supporting assignment	Validation and interpretation
Yoshida et al. (2021) [12]	I: P; M: P	Individual serum miRNAs and combined prognostic indices evaluated for OS/PFS.	Retrospective prognostic modelling within the selected serum dataset; no recurrence-monitoring experiment. Primary platform: Microarray. Clinical blood matrix: Serum.
Kandimalla et al. (2021)[14]	M: D, E	OCaMIR serum panel evaluated for OC detection, including stage I performance.	Multiple retrospective and prospective serum cohorts. Tissue discovery/validation is distinguished from serum validation; ordinary logistic regression is not counted in the algorithmic row. Primary platform: NGS. Clinical blood matrix: Serum. NGS classification reflects the initial candidate-discovery workflow, including tissue discovery; circulating validation used other assays.
Zheng et al. (2013)[95]	I: D, P; M: D, E	Individual plasma miRNAs and the miR-205/let-7f diagnostic combination; the combination was evaluated in stage I versus controls (Figure 3). Individual let-7f survival analysis.	Validation at two centres. Low-CA125 subgroup performance is not a fitted miRNA-plus-CA125 model; no protein-combination cell assigned. Individual-marker stage-specific accuracy was not established. Primary platform: qRT-PCR. Clinical blood matrix: Plasma.
Zhang et al. (2020)[81]	I: D, P; V: D, P; C: D, P	Exosomal miR-484 diagnostic ROC and OS/PFS analyses; addition of CA125 for diagnosis and joint miR-484/CA125 survival strata.	Single cohort. Joint survival strata are not an externally validated prognostic prediction model. Primary platform: qRT-PCR. Clinical blood matrix: Serum.
Ren et al. (2018) [3]	I: D; C: D	Serum miR-193a-5p and its combination with HE4/CA125 assessed by diagnostic ROC analysis.	Same-cohort model evaluation; inclusion of early-stage cases does not establish stage-specific diagnostic performance. Primary platform: qRT-PCR. Clinical blood matrix: Serum.
Zhu et al. (2022) [47]	I: D; V: D; C: D	Plasma exosomal miR-205 diagnostic analysis, including combination with conventional CA125/HE4 markers.	Same-cohort evaluation. Stage associations alone are not counted as early-stage diagnostic performance. Primary platform: qRT-PCR. Clinical blood matrix: Plasma.
Márton et al. (2019)[44]	I: D	Plasma EMT-related miRNAs assessed separately for malignant/nonmalignant discrimination; no combined classifier established.	Single cohort; agreement with CA125/HE4 is not a combined protein-miRNA model. Primary platform: qRT-PCR. Clinical blood matrix: Plasma.

Study and reference	Assigned cells	Source findings supporting assignment	Validation and interpretation
Todeschini et al. (2017)[48]	I: D; M: D	Serum miR-1246 and other individual markers plus integrated three-miRNA ROC model.	Training and validation collections from two Italian sources; assay confirmation also performed. Predominantly advanced HGSOC. Primary platform: Microarray. Clinical blood matrix: Serum.
Gockley et al. (2022)[51]	M: D, E; A: D, E	Neural-network miRNA panel applied to human early-stage cases and benign/healthy comparators.	Mouse-informed model development followed by human evaluation; not population screening effectiveness. Primary platform: NGS. Clinical blood matrix: Serum.
Elias et al. (2017) [52]	M: D, E; A: D, E	Neural-network serum classifier; main Results report early/advanced-stage secondary analyses and refer to Supplementary file 2.	Internal model testing, qPCR adaptation and independent clinical sample evaluation. Stage-specific numerical details remain in the source supplement. Primary platform: NGS. Clinical blood matrix: Serum.
Choi et al. (2020) [49]	I: D; M: D; A: D	Individual miR-200 family members and population-specific neural-network diagnostic models.	Internal training/test division; separate American/Chinese models do not establish transport of one fixed model between populations. Primary platform: qRT-PCR. Clinical blood matrix: Serum and plasma.
Wang et al. (2022) [53]	I: D, P; M: D, E; V: D, P	EV diagnostic signature and individual miRNAs; plasma EV miR-1290 OS/PFS analysis. Early-stage classifier results come from public serum datasets, not a separate EV assay validation.	The authors explicitly re-trained models in each external dataset. Tissue and urine analyses do not constitute validation of the plasma EV assay. Primary platform: NGS. Clinical blood matrix: Plasma.
Webber et al. (2024)[4]	M: D; C: D, E; A: D, E	Neural-network miRNA-only and integrated miRNA/protein/metadata models; integrated model evaluated by stage.	Internal and external validation sets; external early-stage results belong to the integrated model. Primary platform: FirePlex. Clinical blood matrix: Serum.
Kumar et al. (2022)[36]	I: D, E; M: D, E	Serum miR-34a, let-7f and miR-31, individually and jointly, with early/late-stage ROC results.	Tissue-based discovery and serum evaluation; related Kumar publication may share participants. Primary platform: NGS. Clinical blood matrix: Serum. NGS classification reflects the initial candidate-discovery workflow, including tissue discovery; circulating validation used other assays.

Study and reference	Assigned cells	Source findings supporting assignment	Validation and interpretation
Kumar et al. (2021)[13]	I: D, E; M: D, E	Serum miR-205, miR-200c and miR-141, individually and jointly, with early/late-stage ROC results.	Tissue-based discovery and serum evaluation; related Kumar publication may share participants. Primary platform: NGS. Clinical blood matrix: Serum. NGS classification reflects the initial candidate-discovery workflow, including tissue discovery; circulating validation used other assays.
Yokoi et al. (2017) [50]	I: D; M: D, E; C: D	Serum multimiRNA models, including early-stage versus benign discrimination; separate miRNA plus CA125 diagnostic model.	Clinical model development/evaluation. EV experiments in cells/mice do not turn the human serum test into an isolated-EV assay. Primary platform: NGS. Clinical blood matrix: Serum.
Hong et al. (2022) [54]	M: D, E; A: D, E	Serum miRNA-pair classifier with voting rule; stage I classification reported.	Internal and external public serum datasets; reused source cohorts are not new independent patient recruitment. Primary platform: Microarray. Clinical blood matrix: Serum.
Hamidi et al. (2021)[55]	M: D; A: D	LASSO/elastic-net selection and machine-learning serum classifiers.	Internal resampling and external public serum dataset evaluation; no specific early-stage diagnostic result counted. Primary platform: Microarray. Clinical blood matrix: Serum.
Yang et al. (2024) [56]	I: D, E; V: D, E; C: D, E	Plasma exosomal miR-223 alone and with CA125, including early-stage versus healthy-control ROC results (Table 5).	Single-cohort evaluation; not a screening study. Primary platform: qRT-PCR. Clinical blood matrix: Plasma.
Penyige et al. (2019)[45]	I: D	Plasma miRNA expression profiling and pathway/network analyses; no integrated diagnostic classifier established.	Exploratory profiling and assay checks. FIGO-specific expression does not establish early-stage diagnostic accuracy. Primary platform: NanoString. Clinical blood matrix: Plasma.
Wiczling et al. (2019)[57]	I: D; M: D	Individual plasma miRNAs and combinations assessed using a Bayesian multilevel model and cross-validated AUC.	Internal cross-validation. Bayesian association modelling is not assigned to the algorithmic row merely because it is multivariable. Primary platform: qRT-PCR. Clinical blood matrix: Plasma.
Ali et al. (2022) [58]	I: D, E; C: D, E	miR-204 alone and with CA125/CA19.9, including early-stage diagnostic ROC analyses.	Single-cohort evaluation; protein combination includes CA19.9. Primary platform: qRT-PCR. Clinical blood matrix: Serum.

Study and reference	Assigned cells	Source findings supporting assignment	Validation and interpretation
Gumusoglu-Acar et al. (2023)[46]	I: D	Individual circulating candidates confirmed after microarray screening.	Small discovery/RT-qPCR samples; several candidates measured together do not establish a combined prediction model. Primary platform: Microarray. Clinical blood matrix: Plasma. Matrix coded as plasma because collection used EDTA; the source inconsistently also calls it serum.
Kan et al. (2012) [59]	I: D; M: D	Individual serum miR-200 family members and a miR-200b/miR-200c classifier.	Leave-one-out cross-validation; no separate stage-specific diagnostic accuracy counted. Primary platform: qRT-PCR. Clinical blood matrix: Serum.
Khan et al. (2023) [60]	I: D	Blood miR-223 expression compared between OC and controls, alongside other molecular targets.	Expression associations only for the mapped diagnostic cell; proposed prognostic/treatment uses are not observed clinical outcome results. Primary platform: qRT-PCR. Clinical blood matrix: Whole blood.
Langhe et al. (2015)[61]	I: D	Four individual serum miRNAs differentially expressed in malignant versus benign disease.	Discovery and validation assay sets; a list called a panel is not automatically a combined classifier. Primary platform: qRT-PCR. Clinical blood matrix: Serum.
Liang et al. (2020) [5]	I: D; C: D	Individual serum markers and miR-183/HE4/CA125 logistic diagnostic model.	Training and validation groups; recruitment from two hospitals does not by itself prove external validation. No stage-specific diagnostic ROC counted. Primary platform: qRT-PCR. Clinical blood matrix: Serum.
Liu et al. (2021) [62]	I: D; V: D	Plasma exosomal miR-4732-5p diagnostic result.	Discovery and qPCR verification. This is an individual miRNA result, not a validated multimiRNA panel. Primary platform: NGS. Clinical blood matrix: Plasma.
Paliwal et al. (2020)[63]	I: D	Serum miR-21 and miR-22 expression and clinicopathological associations.	Single case-control series; no combined classifier or clinical survival endpoint established. Primary platform: qRT-PCR. Clinical blood matrix: Serum.
Ritter et al. (2020) [64]	I: D	Serum/urine candidate miRNAs and RT-qPCR expression comparisons in female cancers, including OC.	Discovery and assay-validation samples. Cross-cancer expression findings are not external validation of an OC model. Primary platform: Microarray. Clinical blood matrix: Serum.

Study and reference	Assigned cells	Source findings supporting assignment	Validation and interpretation
Stawiski et al. (2024)[65]	M: D; A: D; B: D	Prediagnostic miRNA panel/learning approaches distinguish samples collected less than two years versus two to seven years before diagnosis.	Case-only time-to-diagnosis comparison; not OC versus healthy-control accuracy, FIGO-defined early-stage accuracy, or established screening validation. Primary platform: NGS. Clinical blood matrix: Serum.
Su et al. (2019) [6]	I: D; M: D; V: D; C: D	Exosomal miR-1307 and miR-375 alone, together and with CA125/HE4; diagnostic ROC analyses (Table 3).	Clinical diagnostic cohort. Stage correlation is not stage-specific accuracy. Primary platform: qRT-PCR. Clinical blood matrix: Serum.
Takamizawa et al. (2024)[66]	I: D	Serum miR-146a-5p and miR-191-5p diagnostic ROC analyses in clear-cell carcinoma.	Single cohort. Predominantly early-stage composition is not a separately analysed early-stage diagnostic result. Primary platform: qRT-PCR. Clinical blood matrix: Serum.
Wang et al. (2019a)[37]	I: D; M: D; V: D	Five-miRNA serum classifier; separate exosomal expression comparison.	Training/validation stages from the study collection. Exosomal expression replication is not external validation of the serum model. Primary platform: qRT-PCR. Clinical blood matrix: Serum.
Yoshimura et al. (2018)[67]	I: D, E, T; C: D, E	Serum miR-99a-5p diagnostic results, including early-stage ROC alone/with CA125; postoperative change.	Human total-serum measurement; cell-derived exosome experiments are not a human isolated-EV diagnostic test. Primary platform: Microarray. Clinical blood matrix: Serum.
Zhang & Hu (2022)[68]	M: D; A: D	Four-miRNA linear-discriminant multicancer model evaluated in ovarian cancer datasets.	Developed using lung cancer data; OC assessment is a separate application. No CA125/HE4 combination and no OC-specific early-stage result counted. Primary platform: Microarray. Clinical blood matrix: Serum.
Chen et al. (2016) [69]	I: P	Serum miR-183 OS association and Cox analysis.	Cell-line bufalin sensitivity is not clinical treatment-response biomarker evidence. Primary platform: qRT-PCR. Clinical blood matrix: Serum.
Gong et al. (2016) [70]	I: D, P	Plasma miR-148a expression comparison and OS analysis.	Single clinical series with mechanistic cell experiments; no clinical response prediction counted. Primary platform: qRT-PCR. Clinical blood matrix: Plasma.

Study and reference	Assigned cells	Source findings supporting assignment	Validation and interpretation
Zhong et al. (2025) [71]	I: P, T; M: P	Individual miR-21/miR-145 chemotherapy-response comparisons and three-year vital-status analyses; combined prognostic ROC.	Same-cohort analysis; vital status is not an OS/PFS duration, and prognostic ROC is not cancer detection. Primary platform: qRT-PCR. Clinical blood matrix: Serum.
Fukagawa et al. (2017) [43]	I: D, P	Serum miR-135a-3p case/comparator expression and PFS analysis.	Chemosensitivity experiments in cells do not establish clinical treatment-response prediction. Primary platform: Microarray. Clinical blood matrix: Serum.
Pal et al. (2024) [72]	I: D, P, T	Individual circulating miRNAs compared with controls, across treatment response/time points and for OS.	Five markers were measured, but mapped clinical results are individual-marker results; no combined five-miRNA score assumed. Primary platform: qRT-PCR. Clinical blood matrix: Blood, unspecified.
Kapetanakis et al. (2015) [73]	I: D, P, T	Plasma miR-200b diagnostic comparison, pre/post-treatment change and PFS association.	Longitudinal treatment sampling is not serial recurrence detection; CA125 comparison is not a combined classifier. Primary platform: qRT-PCR. Clinical blood matrix: Plasma.
Chao et al. (2014) [74]	I: R, T	Pre/postoperative individual miRNAs; serial miR-130a measurements during follow-up, including recurrence cases.	Small discovery, validation and longitudinal application sets; recurrence monitoring remains exploratory. Primary platform: qRT-PCR. Clinical blood matrix: Serum.
Robelin et al. (2020) [75]	I: P, T	Individual miRNA kinetics assessed against treatment response, surgical outcome and OS/PFS; several analyses negative.	CHIVA trial biomarker substudy; an 11-analyte assay is not automatically an 11-miRNA prediction model. Primary platform: qRT-PCR. Clinical blood matrix: Plasma.
Benson et al. (2015) [76]	I: P, T	Individual plasma miRNA changes after chemotherapy and associations with response/PFS.	Small recurrent platinum-resistant OC trial substudy; recurrent disease at enrolment is not recurrence monitoring. Primary platform: qRT-PCR. Clinical blood matrix: Plasma.
Halvorsen et al. (2017) [77]	I: P, T	Individual plasma miRNAs evaluated for PFS and bevacizumab-associated benefit.	Discovery/validation subsets of ICON7; treatment-predictive results are distinguished from general prognosis. Primary platform: qRT-PCR. Clinical blood matrix: Plasma.

Study and reference	Assigned cells	Source findings supporting assignment	Validation and interpretation
Uehara et al. (2024) [78]	I: P, T; M: P, T; A: P, T	Individual and LASSO-derived multimiRNA PFS models, treatment selection and resection-related analyses.	Propensity matching/internal modelling; estimated benefit from alternative treatment is not prospectively observed treatment benefit. Primary platform: Microarray. Clinical blood matrix: Serum.
Zuberi et al. (2020)[41]	I: D, P	Serum miR-145 diagnostic and survival analyses, including nonsignificant survival findings.	Single cohort. ROC discrimination between disease stages is not early-stage cancer versus noncancer detection. Primary platform: qRT-PCR. Clinical blood matrix: Serum.
Zhao et al. (2018)[79]	I: D, P	Plasma miR-106a diagnostic ROC and OS/Cox analyses.	Single cohort; stage association is not early-stage diagnostic accuracy. Primary platform: qRT-PCR. Clinical blood matrix: Plasma.
Helmy et al. (2025)[80]	I: D, P; C: D	miR-200b diagnostic comparisons and EFS analysis; protein combinations evaluated diagnostically.	EFS is a composite recurrence/death endpoint, not longitudinal recurrence monitoring. Do not transfer the individual-marker EFS result to the diagnostic combination. Primary platform: qRT-PCR. Clinical blood matrix: Serum.
Shah et al. (2018)[82]	I: D, T; C: D, T	Serum miRNAs and CA125 combinations for OC diagnosis and completeness of cytoreduction.	Surgical-outcome prediction, not an OS/PFS prognostic analysis or early-stage detection analysis. Primary platform: qRT-PCR. Clinical blood matrix: Serum.
Li et al. (2023)[83]	I: D, T; M: D, E, P, T; V: D, E, P, T; A: D, E, P, T	sEV diagnostic LASSO score, stage I discrimination, pre/postoperative changes and score-based PFS analyses (Figures 2–5).	Clinical discovery/test/validation cohorts. Individual-miRNA OS findings using TCGA tissue are not circulating-miRNA prognostic evidence; circulating score-based PFS is retained. Primary platform: NGS. Clinical blood matrix: Serum.
Hong et al. (2013)[84]	I: D, P	Serum miR-221 case-control expression and OS/Cox analyses.	Single cohort; no serial monitoring or protein-combination model counted. Primary platform: qRT-PCR. Clinical blood matrix: Serum.
Meng et al. (2016)[39]	I: D, P; M: D; V: D, P	Individual exosomal miRNAs and combined diagnostic ROC; individual-marker OS/recurrence-free survival analyses.	Recurrence-free survival is prognostic evidence, not serial recurrence monitoring. Related Meng publications may share participants. Primary platform: qRT-PCR. Clinical blood matrix: Serum.

Study and reference	Assigned cells	Source findings supporting assignment	Validation and interpretation
Chen et al. (2020)[85]	I: D, P, T; C: D	Serum miR-125b diagnostic, survival, pre/postoperative and chemotherapy-sensitivity analyses; diagnostic combination with CA125.	Response and survival belong to miR-125b analyses; no combined-protein response model assumed. Primary platform: qRT-PCR. Clinical blood matrix: Serum.
Zhu et al. (2017)[86]	I: D, P	Serum miR-125b diagnostic comparison and PFS/OS associations.	Stage/residual-tumour associations are not separately validated early detection or treatment-selection models. Primary platform: qRT-PCR. Clinical blood matrix: Serum.
Song et al. (2020)[87]	I: D, P; M: D	Serum miR-26b and miR-21 alone/combined for diagnosis; individual survival analyses.	The combined diagnostic ROC is not a combined survival model. Primary platform: qRT-PCR. Clinical blood matrix: Serum.
Maeda et al. (2020)[88]	I: P; V: P	Serum exosomal miR-34a compared by subsequent three-year recurrence status.	Patients with OC only: recurrence-risk association, not serial recurrence detection or cancer-versus-control accuracy. Primary platform: qRT-PCR. Clinical blood matrix: Serum.
Gao & Wu (2015)[89]	I: D, P	Serum miR-200c and miR-141 diagnostic ROC and survival analyses.	Separate marker results; neither simultaneous measurement nor stage association establishes a combined early-stage classifier. Primary platform: qRT-PCR. Clinical blood matrix: Serum.
Guo et al. (2013)[90]	I: D	Serum miR-92 case-control expression and clinicopathological associations.	No observed survival endpoint established; discussion of prognosis is not a prognostic result. Primary platform: qRT-PCR. Clinical blood matrix: Serum.
Habel et al. (2023)[91]	I: D, T	Individual plasma miRNAs compared with controls and platinum-sensitive/resistant disease.	Clinical chemotherapy-sensitivity analysis retained; no survival endpoint or combined model assumed. Primary platform: qRT-PCR. Clinical blood matrix: Plasma.
Meng et al. (2015)[40]	I: D, P; M: D	Individual serum markers and four-miRNA diagnostic classifier; individual miR-429 OS analysis.	Ordinary logistic regression, not a neural network. miR-429 plus CA125 differentiates early from advanced disease, not early cancer from noncancer; not mapped as early detection. Primary platform: qRT-PCR. Clinical blood matrix: Serum.
Minareci et al. (2024)[92]	I: D, E, P; M: D, E	Individual miR-1181/miR-4314 diagnostic and survival analyses; combined stage I versus healthy-control diagnostic analysis.	Combined stage I ROC is explicitly reported; this is case-control discrimination, not population screening. Primary platform: qRT-PCR. Clinical blood matrix: Serum.

Study and reference	Assigned cells	Source findings supporting assignment	Validation and interpretation
Ranmale et al. (2025)[93]	I: D, P, T; M: D	Individual serum miRNAs for diagnosis, PFS and chemotherapy-related comparisons; combined three-miRNA diagnostic ROC.	Serum miR-130a prognosis distinguished from tissue miR-20a prognosis; no prognostic role assigned to the diagnostic panel. Primary platform: qRT-PCR. Clinical blood matrix: Serum.
Wang et al. (2019b)[38]	I: D; M: D; V: D	Plasma five-miRNA diagnostic panel and individual expression comparisons, including an exosomal subset.	Training/validation stages; tumour-grade associations are not observed survival prognosis or early-stage diagnostic accuracy. Primary platform: qRT-PCR. Clinical blood matrix: Plasma. Clinical purpose: diagnostic only (Table 1 and Figure 2A).
Yunusova et al. (2023)[94]	I: D; V: D	Plasma/ascites exosomal individual miRNAs and tetraspanin associations; plasma case-control expression.	No clinical survival result or joint CA125/HE4 classifier established; mechanistic interpretation does not establish prognosis. Primary platform: qRT-PCR. Clinical blood matrix: Plasma.
Zuberi et al. (2016)[42]	I: D, P	Serum miR-199a diagnostic and survival analyses.	Stage-discrimination ROC is not early cancer versus noncancer accuracy; single cohort. Primary platform: qRT-PCR. Clinical blood matrix: Serum.
Xu et al. (2013) [18]	I: D, P	Serum miR-21 case-control expression and overall-survival association.	No diagnostic ROC or independent validation; ΔCt HR direction and miR-221 labelling inconsistencies limit interpretation. Primary platform: qRT-PCR. Clinical blood matrix: Serum.
Zuberi et al. (2016) [19]	I: D, P	Serum miR-125b diagnostic AUC 0.728; null overall-survival association.	No independent validation; probable cohort overlap with [41,42]. Stage/metastasis associations do not establish early-stage diagnostic accuracy. Primary platform: qRT-PCR. Clinical blood matrix: Serum.
Zhou et al. (2023) [20]	I: D, T; M: T; C: D, T; V: D, T; A: D, T	Plasma sEV four-miRNA/CA-125 surgical residual-disease model; ancillary circulating-marker and combined-risk-index diagnostic group comparisons.	Separate validation n=154; external-by-centre validation not established. Surgical AUC 0.903 is not cancer-detection accuracy; early-stage comparator samples do not establish early-stage diagnostic performance. Primary platform: NGS. Clinical blood matrix: Plasma.

Study and reference	Assigned cells	Source findings supporting assignment	Validation and interpretation
Günel et al. (2018) [21]	I: D	Existing recurrence versus healthy controls.	No prospective prognostic or serial-monitoring result. Primary platform: Microarray. Clinical blood matrix: Serum. Purpose: diagnostic; stage unclear; Turkey (Asia); serous; case-control; <200; normalization reported (cel-miR-39).
Suryawanshi et al. (2013) [22]	I: D; M: D; A: D	Individual-marker expression and LDA panel discrimination.	Discovery participants reused in expanded cohort. Primary platform: qRT-PCR. Clinical blood matrix: Plasma. Purpose: diagnostic; mixed stages; USA; mixed epithelial; case-control; <200; normalization reported (miR-132). Ancillary tissue/mouse experiments excluded; no stage-specific accuracy.
Shapira et al. (2014) [23]	I: D, P, T	Differential expression versus healthy/benign comparators; no diagnostic AUC. Survival-group AUCs 0.13, 0.15, 0.73, 0.74 and 0.87 are not diagnostic AUCs.	Exploratory same-cohort analysis; no independent validation. Ultracentrifuged plasma supernatant, not the isolated EV fraction. Purpose: Both; stage: Mixed; region: North America; histology: Serous; design: Case-control; <200; primary platform: qRT-PCR; clinical blood matrix: Plasma; normalization: Source-selected reference-miRNA normalization.
Kumari et al. (2022) [24]	I: D	miR-145 AUC 0.790 (95% CI 0.653–0.927); source sensitivity 90%, specificity 73% (Table IV).	No independent validation. Endometriosis and endometrial-cancer results excluded from the OC extraction. Purpose: Diagnostic; stage: NR; region: Asia; histology: Other; design: Case-control; <200; primary platform: qRT-PCR; clinical blood matrix: Serum; normalization: cel-miR-39 spike-in and U6 reference. Source ROC comparator composition is ambiguous; the OC AUC is retained descriptively but excluded from Figure 8.
Meng et al. (2016) [25]	I: D; M: D	miR-200a/b/c AUCs 0.853/0.953/0.903; combined panel 0.959 (Figure 1.1).	Book-chapter report; possible reuse of the Hamburg cohort in [39,40]. No independent validation; comparator labels differ between figure and narrative. Purpose: Diagnostic; stage: Mixed; region: Europe; histology: Serous; design: Case-control; <200; primary platform: qRT-PCR; clinical blood matrix: Serum; normalization: miR-484 and cel-miR-39.

Study and reference	Assigned cells	Source findings supporting assignment	Validation and interpretation
Xiong et al. (2021) [26]	I: D; V: D	Increased expression in OC-derived plasma exosomes; no eligible AUC or sensitivity/specificity estimate.	Human plasma component only; cell-line, macrophage and animal experiments not counted as clinical prediction evidence. Purpose: Diagnostic; stage: NR; region: Asia; histology: NR; design: Experimental; <200; primary platform: qRT-PCR; clinical blood matrix: Plasma; normalization: U6 reference.
Ruan et al. (2018) [27]	I: D	Differential expression and same-dataset clustering; no diagnostic ROC AUC. Reported clustering accuracy is not independent validation.	Secondary analysis of the Fukagawa cohort [43]; do not count as independent recruitment. Group counts retained despite the source total discrepancy. Purpose: Diagnostic; stage: Mixed; region: Asia; histology: NR; design: Retrospective; <200; primary platform: Microarray; clinical blood matrix: Serum; normalization: Microarray preprocessing/normalization with limma.
Ji et al. (2014) [28]	I: D	Differential expression; no diagnostic AUC.	Pooled sequencing discovery followed by qPCR; independence of confirmation samples not established. Treatment-history wording conflicts with the source clinical table. Purpose: Diagnostic; stage: Mixed; region: Asia; histology: NR; design: Case-control; <200; primary platform: NGS; clinical blood matrix: Serum; normalization: miR-16 reference.
Tian et al. (2025) [29]	I: D, E	miR-338-3p AUC 0.905 overall and 0.600 early-stage; CMD AUC 0.9789 overall and 1.000 early-stage (Figures 8B,C).	Same-cohort logistic models; no independent validation. CMD is a miRNA–circRNA model, not a multi-miRNA or miRNA–protein model. Purpose: Diagnostic; stage: Mixed; region: Asia; histology: NR; design: Case-control; <200; primary platform: NGS; clinical blood matrix: Plasma; normalization: U6 for miRNA; GAPDH for circRNA.
Sun et al. (2024) [30]	I: D, T	Higher plasma expression in OC and in paclitaxel-resistant versus sensitive cases; no eligible AUC or adjusted clinical effect estimate.	Only patient plasma comparisons retained; CAF/EV functional experiments do not establish clinical EV diagnostic accuracy. Purpose: Both; stage: NR; region: Asia; histology: NR; design: Experimental; <200; primary platform: qRT-PCR; clinical blood matrix: Plasma; normalization: Not specified in accessible main article; supplementary methods unavailable.

Study and reference	Assigned cells	Source findings supporting assignment	Validation and interpretation
Timofeeva et al. (2023) [31]	I: T	Six miRNA-containing response models: source Table 8 models 2,3,5,6,7,8; sensitivity 64.29–92.86%, specificity 75–100%.	RECIST response prediction; same-cohort fitting without independent validation. piRNA-only models and tissue/PGR-phenotype analyses are not clinical circulating-miRNA outcome models. Purpose: Prognostic; stage: Mixed; region: Europe; histology: Serous; design: Observational; <200; primary platform: NGS; clinical blood matrix: Plasma; normalization: Plasma miR-30d-5p; piR-004308 for piRNA.
Timofeeva et al. (2022) [32]	I: D, T; M: T	Plasma expression comparisons; no cancer-versus-noncancer AUC. Surgical-outcome model AUCs 0.77/0.77/0.74/0.73; chemotherapy-response model AUCs 0.91/0.90/0.90/0.90 (Figure 7).	Four models per clinical endpoint, fitted and evaluated in the same small cohort; no independent validation. Tissue-derived diagnostic AUCs excluded. Purpose: Both; stage: Mixed; region: Europe; histology: Serous; design: Observational; <200; primary platform: NGS; clinical blood matrix: Plasma; normalization: Plasma miR-30d-5p reference.
Gumusoglu et al. (2021) [33]	I: D	Expression profiling; no diagnostic AUC or miRNA-specific clinical prognostic estimate.	No independent validation. Source Table 1 lists 9 serous and 1 endometrioid case, despite narrower histology wording in Methods. Possible cohort overlap with Istanbul reports. Purpose: Diagnostic; stage: Mixed; region: Asia; histology: Serous; design: Case-control; <200; primary platform: Microarray; clinical blood matrix: Serum; normalization: Quantile normalization.
An et al. (2020) [34]	I: D	Lower serum expression in OC; stage association does not establish prediction of a future outcome. No AUC.	Only serum results retained; ancillary tissue/cell experiments excluded from clinical biomarker counts. Purpose: Diagnostic; stage: Mixed; region: Asia; histology: NR; design: Experimental; <200; primary platform: qRT-PCR; clinical blood matrix: Serum; normalization: U6 reference.

Study and reference	Assigned cells	Source findings supporting assignment	Validation and interpretation
Günel et al. (2019) [35]	I: D	AUC 0.700 (95% CI 0.4595–0.9405); no independently validated threshold performance.	qPCR confirmation does not establish an independent cohort. Source calls the specimen serum but describes EDTA collection; coded plasma consistently with [46]. Possible Istanbul-cohort overlap. Purpose: Diagnostic; stage: NR; region: Asia; histology: HGSOC; design: Case-control; <200; primary platform: Microarray; clinical blood matrix: Plasma; normalization: Quantile normalization and cel-miR-39 reference.

Figure 2G and Table 4 platform assignments use one primary candidate-discovery/profiling category per publication. PCR-based TaqMan/Exiqon screening arrays are qRT-PCR, not hybridization microarrays. NanoString, digital PCR and FirePlex are separate categories. Figure 2H and Table 4 classify the clinical blood matrix; ancillary tissue, urine or ascites experiments do not create separate blood-matrix categories. All 91 study-level assignments are recorded above.

The miRNA–circRNA diagnostic model in [29] and miRNA–piRNA response models in [31] are described in the study notes but are not relabelled as multi-miRNA or miRNA–protein combinations. Individual-miRNA components contribute to the applicable cells. Publications sharing or potentially sharing participants are not independent cohorts. The 91 publication-level classifications correspond to Tables 1–4 and Figure 2.
