# Supplementary material for: Circulating miRNAs and Multivariate Biomarker Signatures in Ovarian Cancer: A Systematic Review
Source: Int J Mol Sci. 2026 Oct 2;27(19):8823. doi: 10.3390/ijms27198823 (PMC13664815; doi:10.3390/ijms27198823)
Supplement: Supplementary file 1 [file ijms-27-08823-s001.zip › Supplementary_Table_S4_Risk_of_Bias_Assessments.pdf]

Supplementary Table S4

Risk-of-bias assessments for Circulating miRNAs and Multivariate Biomarker Signatures in Ovarian Cancer: A Systematic Review.

Parts A–C present domain judgments for 34 QUADAS-2 study assessments, 33 QUIPS study assessments and 65 PROBAST assessment entries. Each part contains a judgment matrix followed by the corresponding target results and rationales. Reference numbers refer to the manuscript. Entries from the same study may appear under more than one tool.

L, low; H, high; U, unclear; M, moderate. Risk of bias and applicability concerns are separate judgments. QUIPS is presented by domain without an overall rating. The rationales below are domain summaries rather than individual signalling-question responses.

Part A QUADAS-2

PS, patient selection; IT, index test; RS, reference standard; FT, flow and timing; App, applicability concerns.

Entry	Study [reference]	PS	IT	RS	FT	Overall	App PS	App IT	App RS
A01	Ali et al. (2022) [58]	H	U	L	U	H	H	L	L
A02	Gumusoglu-Acar et al. (2023) [46]	H	H	L	U	H	H	L	L
A03	Khan et al. (2023) [60]	H	U	U	U	H	H	L	L
A04	Márton et al. (2019) [44]	H	U	U	U	H	H	L	L
A05	Oliveira et al. (2019) [10]	H	U	L	U	H	L	L	L
A06	Ren et al. (2018) [3]	H	U	U	U	H	H	L	L
A07	Su et al. (2019) [6]	H	U	U	U	H	H	L	L
A08	Takamizawa et al. (2024) [66]	H	U	L	U	H	H	L	L
A09	Todeschini et al. (2017) [48]	H	L	U	U	H	H	L	L
A10	Wiczling et al. (2019) [57]	H	U	U	U	H	H	L	L
A11	Yang et al. (2024) [56]	H	U	U	U	H	H	L	L
A12	Yoshimura et al. (2018) [67]	H	U	U	U	H	H	L	L
A13	Zhu et al. (2022) [47]	H	U	L	U	H	H	L	L
A14	Kapetanakis et al. (2015) [73]	H	U	U	U	H	L	L	L
A15	Chen et al. (2020) [85]	U	U	U	U	U	L	L	L
A16	Gao & Wu (2015) [89]	U	U	U	U	U	L	L	L
A17	Guo et al. (2013) [90]	H	U	U	U	H	L	L	L

Entry	Study [reference]	PS	IT	RS	FT	Overall	App PS	App IT	App RS
A18	Helmy et al. (2025) [80]	U	U	U	U	U	L	L	L
A19	Hong et al. (2013) [84]	H	U	L	U	H	L	L	L
A20	Meng et al. (2016) [39]	H	H	L	U	H	L	L	L
A21	Minareci et al. (2024) [92]	U	U	U	U	U	L	L	L
A22	Song et al. (2020) [87]	U	U	U	U	U	L	L	L
A23	Zhang et al. (2020) [81]	U	U	U	U	U	L	L	L
A24	Zhao et al. (2018) [79]	U	U	U	U	U	L	L	L
A25	Zhu et al. (2017) [86]	U	U	U	U	U	L	L	L
A26	Zuberi et al. (2016) [42]	U	U	L	U	U	L	L	L
A27	Zuberi et al. (2020) [41]	U	U	L	U	U	L	L	L
A28	Zuberi et al. (2016) [19]	H	H	U	U	H	H	L	U
A29	Liu et al. (2021) [62]	H	U	U	U	H	H	L	L
A30	Günel et al. (2018) [21]	H	H	U	U	H	H	L	U
A31	Kumari et al. (2022) [24]	H	H	L	U	H	H	L	L
A32	Meng et al. (2016) [25]	H	H	U	U	H	H	L	U
A33	Tian et al. (2025) [29]	H	H	L	U	H	H	L	L
A34	Günel et al. (2019) [35]	H	H	U	U	H	H	L	U

Part A Supporting assessment details

Entry	Study [reference]	Target result or model	Recorded rationale	Applicability rationale
A01	Ali et al. (2022) [58]	Comparator: OC vs benign ovarian tumours vs healthy controls; compared with CA125/CA19-9; performance: Sensitivity ≈80%; specificity ≈86.7%; combination improved accuracy	**SQ1 selection:** Separately selected cancer and healthy/control groups create a two-gate or case-control spectrum. **SQ2 index test:** Objective assay/performance analysis was reported, but prespecification and blinded interpretation were not fully verified. **SQ3 reference standard:** Histopathological diagnosis was used as the reference standard. **SQ4 flow/timing:** Complete participant flow, interval, and inclusion in analysis were not fully reported.	**App PS:** High concern because separately selected cancer and control groups may not represent the intended clinical diagnostic population. **App IT:** Low concern; the circulating miRNA assay was relevant to the review question. **App RS:** Low concern; histopathological diagnosis was an appropriate reference standard.
A02	Gumusoglu-Acar et al. (2023) [46]	Comparator: Healthy individuals; simple ovarian cyst tissue controls; performance: AUC: miR-1909-5p = 1.00; miR-885-5p = 0.78; let-7d-3p = 0.70; miR-200c-3p = 0.83	**SQ1 selection:** Separately selected cancer and healthy/control groups create a two-gate or case-control spectrum. **SQ2 index test:** The cut-off/threshold was optimized in the analysed data. **SQ3 reference standard:** Histopathological diagnosis was used as the reference standard. **SQ4 flow/timing:** Complete participant flow, interval, and inclusion in analysis were not fully reported.	**App PS:** High concern because separately selected cancer and control groups may not reflect a clinically representative diagnostic spectrum. **App IT:** Low concern; the evaluated circulating miRNA test was relevant. **App RS:** Low concern; the diagnostic reference standard was appropriate.
A03	Khan et al. (2023) [60]	Comparator: OC vs healthy controls; performance: AUC: miR-223 = 0.7915; TPD52 = 0.7769; PKCε = 0.7630; KLF9 = 0.5608	**SQ1 selection:** Separately selected cancer and healthy/control groups create a two-gate or case-control spectrum. **SQ2 index test:** Objective assay/performance analysis was reported, but prespecification and blinded interpretation were not fully verified. **SQ3 reference standard:** The reference standard appears clinical/pathological, but its conduct and blinded interpretation were incompletely reported. **SQ4 flow/timing:** Complete participant flow, interval, and inclusion in analysis were not fully reported.	**App PS:** High concern because ovarian cancer cases were compared with separately selected healthy controls. **App IT:** Low concern; the circulating miRNA assessment matched the review question. **App RS:** Low concern; the clinical/pathological reference diagnosis was relevant to the target condition.
A04	Márton et al. (2019) [44]	Comparator: Healthy controls; non-malignant ovarian masses; CA125/HE4 comparison; performance: Best: miR-200c AUC = 0.861; others: miR-200a 0.845, miR-200b 0.822, miR-141 0.825, miR-203a 0.831, miR-34b 0.832; sensitivity up to 85.7%, specificity up to 90%	**SQ1 selection:** Separately selected cancer and healthy/control groups create a two-gate or case-control spectrum. **SQ2 index test:** Objective assay/performance analysis was reported, but prespecification and blinded interpretation were not fully verified. **SQ3 reference standard:** The reference standard appears clinical/pathological, but its conduct and blinded interpretation were incompletely reported. **SQ4 flow/timing:** Complete participant flow, interval, and inclusion in analysis were not fully reported.	**App PS:** High concern because the case-control spectrum included separately selected healthy/non-malignant controls rather than a fully representative clinical population. **App IT:** Low concern; circulating miRNA testing was relevant. **App RS:** Low concern; the clinical/pathological diagnosis addressed the target condition.

Entry	Study [reference]	Target result or model	Recorded rationale	Applicability rationale
A05	Oliveira et al. (2019) [10]	Comparator: OC vs benign pelvic masses; performance: miR-200c-3p AUC = 0.78; miR-221-3p AUC ≈0.65; 2-miRNA panel AUC ≈0.82; with CA125 AUC ≈0.96, sensitivity ≈80%, specificity ≈95%	**SQ1 selection:** Separately selected cancer and healthy/control groups create a two-gate or case-control spectrum. **SQ2 index test:** Objective assay/performance analysis was reported, but prespecification and blinded interpretation were not fully verified. **SQ3 reference standard:** Histopathological diagnosis was used as the reference standard. **SQ4 flow/timing:** Complete participant flow, interval, and inclusion in analysis were not fully reported.	**App PS:** Low concern; women with malignant or benign pelvic masses match the intended diagnostic setting. Case-control sampling remains a risk-of-bias concern, distinct from applicability. **App IT:** Low concern; the plasma miRNA assay is relevant. **App RS:** Low concern; histopathological diagnosis addresses the target condition.
A06	Ren et al. (2018) [3]	Comparator: EOC vs benign ovarian tumours and healthy controls; performance: miR-193a-5p AUC = 0.708, sensitivity 66.7%, specificity 72.5%; HE4 AUC = 0.877; CA125 AUC = 0.900; combined AUC = 0.996, sensitivity 93.3%, specificity 97.5%	**SQ1 selection:** Separately selected cancer and healthy/control groups create a two-gate or case-control spectrum. **SQ2 index test:** Objective assay/performance analysis was reported, but prespecification and blinded interpretation were not fully verified. **SQ3 reference standard:** The reference standard appears clinical/pathological, but its conduct and blinded interpretation were incompletely reported. **SQ4 flow/timing:** Complete participant flow, interval, and inclusion in analysis were not fully reported.	**App PS:** High concern because cancer cases were compared with separately selected benign and healthy controls. **App IT:** Low concern; the circulating miRNA test was relevant. **App RS:** Low concern; the clinical/pathological diagnosis corresponded to the target condition.
A07	Su et al. (2019) [6]	Comparator: Benign ovarian tumours and healthy controls; compared with CA125/HE4; performance: miR-1307 AUC = 0.671–0.694; miR-375 AUC = 0.788; CA125 AUC = 0.905–0.906; HE4 AUC = 0.820; miR-1307+miR-375 AUC = 0.837; +CA125 AUC ≈0.970–0.977	**SQ1 selection:** Separately selected cancer and healthy/control groups create a two-gate or case-control spectrum. **SQ2 index test:** Objective assay/performance analysis was reported, but prespecification and blinded interpretation were not fully verified. **SQ3 reference standard:** The reference standard appears clinical/pathological, but its conduct and blinded interpretation were incompletely reported. **SQ4 flow/timing:** Complete participant flow, interval, and inclusion in analysis were not fully reported.	**App PS:** High concern because the selected benign and healthy control groups may not represent the intended clinical diagnostic spectrum. **App IT:** Low concern; circulating miRNA testing was directly relevant. **App RS:** Low concern; the clinical/pathological reference diagnosis was relevant.
A08	Takamizawa et al. (2024) [66]	Comparator: Healthy controls and endometriosis patients; performance: miR-146a-5p AUC = 0.762, sensitivity 79.3%, specificity 50%; miR-191-5p AUC = 0.830, sensitivity 89.7%, specificity 50%	**SQ1 selection:** Separately selected cancer and healthy/control groups create a two-gate or case-control spectrum. **SQ2 index test:** Objective assay/performance analysis was reported, but prespecification and blinded interpretation were not fully verified. **SQ3 reference standard:** Histopathological diagnosis was used as the reference standard. **SQ4 flow/timing:** Complete participant flow, interval, and inclusion in analysis were not fully reported.	**App PS:** High concern because the selected control spectrum included healthy and endometriosis groups rather than a fully representative diagnostic population. **App IT:** Low concern; the circulating miRNA assay was relevant. **App RS:** Low concern; histopathological diagnosis was appropriate.

Entry	Study [reference]	Target result or model	Recorded rationale	Applicability rationale
A09	Todeschini et al. (2017) [48]	Comparator: Healthy controls; performance: miR-1246 AUC = 0.89, sensitivity 87%, specificity 77%, accuracy 84%	**SQ1 selection:** Separately selected cancer and healthy/control groups create a two-gate or case-control spectrum. **SQ2 index test:** The marker interpretation was evaluated in an independent validation cohort. **SQ3 reference standard:** The reference standard appears clinical/pathological, but its conduct and blinded interpretation were incompletely reported. **SQ4 flow/timing:** Complete participant flow, interval, and inclusion in analysis were not fully reported.	**App PS:** High concern because ovarian cancer cases were compared with separately selected healthy controls. **App IT:** Low concern; the miRNA biomarker test matched the review question. **App RS:** Low concern; the clinical/pathological diagnosis addressed the target condition.
A10	Wiczling et al. (2019) [57]	Comparator: OC vs healthy controls; performance: Individual AUC ≈0.65; combined subset AUC ≈0.72; overall limited performance	**SQ1 selection:** Separately selected cancer and healthy/control groups create a two-gate or case-control spectrum. **SQ2 index test:** Objective assay/performance analysis was reported, but prespecification and blinded interpretation were not fully verified. **SQ3 reference standard:** The reference standard appears clinical/pathological, but its conduct and blinded interpretation were incompletely reported. **SQ4 flow/timing:** Complete participant flow, interval, and inclusion in analysis were not fully reported.	**App PS:** High concern because the cancer-versus-healthy-control design may not represent routine clinical diagnostic populations. **App IT:** Low concern; circulating miRNA testing was relevant. **App RS:** Low concern; the clinical/pathological diagnosis corresponded to the target condition.
A11	Yang et al. (2024) [56]	Comparator: EOC vs benign tumours vs healthy controls; performance: miR-223 AUC = 0.812; CA125 AUC = 0.933; miR-223+CA125 AUC = 0.937, sensitivity ≈94.9%, specificity ≈81.5%; early-stage AUC = 0.94, sensitivity ≈97.8%, specificity ≈88.5%	**SQ1 selection:** Separately selected cancer and healthy/control groups create a two-gate or case-control spectrum. **SQ2 index test:** Objective assay/performance analysis was reported, but prespecification and blinded interpretation were not fully verified. **SQ3 reference standard:** The reference standard appears clinical/pathological, but its conduct and blinded interpretation were incompletely reported. **SQ4 flow/timing:** Complete participant flow, interval, and inclusion in analysis were not fully reported.	**App PS:** High concern because separately selected cancer, benign and healthy groups may not reflect the intended clinical population. **App IT:** Low concern; circulating miRNA assessment was relevant. **App RS:** Low concern; the clinical/pathological reference diagnosis was appropriate.
A12	Yoshimura et al. (2018) [67]	Comparator: OC vs benign vs healthy; performance: AUC = 0.88; sensitivity 0.85; specificity 0.75	**SQ1 selection:** Separately selected cancer and healthy/control groups create a two-gate or case-control spectrum. **SQ2 index test:** Objective assay/performance analysis was reported, but prespecification and blinded interpretation were not fully verified. **SQ3 reference standard:** The reference standard appears clinical/pathological, but its conduct and blinded interpretation were incompletely reported. **SQ4 flow/timing:** Complete participant flow, interval, and inclusion in analysis were not fully reported.	**App PS:** High concern because separately selected ovarian cancer, benign and healthy groups may not represent routine diagnostic practice. **App IT:** Low concern; the circulating miRNA test was relevant. **App RS:** Low concern; the clinical/pathological reference diagnosis addressed the target condition.

Entry	Study [reference]	Target result or model	Recorded rationale	Applicability rationale
A13	Zhu et al. (2022) [47]	Comparator: OC vs benign vs healthy controls; performance: miR-205 AUC = 0.715, sensitivity 66.7%, specificity 78.1%; combined miR-205+CA125+HE4 AUC up to 0.951	**SQ1 selection:** Separately selected cancer and healthy/control groups create a two-gate or case-control spectrum. **SQ2 index test:** Objective assay/performance analysis was reported, but prespecification and blinded interpretation were not fully verified. **SQ3 reference standard:** Histopathological diagnosis was used as the reference standard. **SQ4 flow/timing:** Complete participant flow, interval, and inclusion in analysis were not fully reported.	**App PS:** High concern because separately selected cancer, benign and healthy groups may not represent the intended diagnostic population. **App IT:** Low concern; the circulating miRNA assay was relevant. **App RS:** Low concern; histopathological diagnosis was appropriate.
A14	Kapetanakis et al. (2015) [73]	Outcomes: Diagnostic discrimination, longitudinal changes, PFS; analysis: Kruskal–Wallis, linear regression, Kaplan–Meier, log-rank, Cox models, landmark analysis	**SQ1 selection:** Separately selected cancer and healthy/control groups create a two-gate or case-control spectrum. **SQ2 index test:** Objective assay/performance analysis was reported, but prespecification and blinded interpretation were not fully verified. **SQ3 reference standard:** The reference standard appears clinical/pathological, but its conduct and blinded interpretation were incompletely reported. **SQ4 flow/timing:** Complete participant flow, interval, and inclusion in analysis were not fully reported.	**App PS:** Low concern; the study population and diagnostic/prognostic setting were relevant to the review question. **App IT:** Low concern; the circulating miRNA measurements were relevant. **App RS:** Low concern; the clinical/pathological reference diagnosis addressed ovarian cancer.
A15	Chen et al. (2020) [85]	Diagnostic: miR-125b: AUC≈0.73, Se1 76%, Sp2 41.6%; combined with CA125: AUC≈0.90, Se 83.6%, Sp 95.6%; prognostic: Low miR-125b linked to advanced stage, LN metastasis, chemoresistance, poorer prognosis; higher post-surgery	**SQ1 selection:** Consecutive/random enrolment and exclusions could not be verified. **SQ2 index test:** Objective assay/performance analysis was reported, but prespecification and blinded interpretation were not fully verified. **SQ3 reference standard:** The reference standard appears clinical/pathological, but its conduct and blinded interpretation were incompletely reported. **SQ4 flow/timing:** Complete participant flow, interval, and inclusion in analysis were not fully reported.	**App PS:** Low concern; the patient population was relevant to the review question. **App IT:** Low concern; the circulating miRNA biomarker assessment was relevant. **App RS:** Low concern; the clinical/pathological diagnosis corresponded to the target condition.
A16	Gao & Wu (2015) [89]	Diagnostic: miR-200c: AUC=0.79, Se 72%, Sp 70%; miR-141: AUC=0.75, Se 69%, Sp 72%; prognostic: High expression associated with shorter survival; miR-200c stronger prognostic marker	**SQ1 selection:** Consecutive/random enrolment and exclusions could not be verified. **SQ2 index test:** Objective assay/performance analysis was reported, but prespecification and blinded interpretation were not fully verified. **SQ3 reference standard:** The reference standard appears clinical/pathological, but its conduct and blinded interpretation were incompletely reported. **SQ4 flow/timing:** Complete participant flow, interval, and inclusion in analysis were not fully reported.	**App PS:** Low concern; the evaluated patient population was relevant to the review question. **App IT:** Low concern; the circulating miRNA biomarkers matched the review scope. **App RS:** Low concern; the clinical/pathological diagnosis addressed the target condition.

Entry	Study [reference]	Target result or model	Recorded rationale	Applicability rationale
A17	Guo et al. (2013) [90]	Diagnostic: AUC=0.803; Se 80.7%; Sp 75%; prognostic: Higher levels linked to advanced stage and LN metastasis	**SQ1 selection:** Separately selected cancer and healthy/control groups create a two-gate or case-control spectrum. **SQ2 index test:** Objective assay/performance analysis was reported, but prespecification and blinded interpretation were not fully verified. **SQ3 reference standard:** The reference standard appears clinical/pathological, but its conduct and blinded interpretation were incompletely reported. **SQ4 flow/timing:** Complete participant flow, interval, and inclusion in analysis were not fully reported.	**App PS:** Low concern; the population was considered applicable to the review question. **App IT:** Low concern; circulating miRNA testing was relevant. **App RS:** Low concern; the clinical/pathological diagnosis corresponded to ovarian cancer.
A18	Helmy et al. (2025) [80]	Diagnostic: miR-200b: AUC=0.743, Se 63.3%, Sp 71%; miR-200b+CA125: Se 100%, Sp 93.1%; prognostic: High miR-200b linked to shorter EFS	**SQ1 selection:** Consecutive/random enrolment and exclusions could not be verified. **SQ2 index test:** Objective assay/performance analysis was reported, but prespecification and blinded interpretation were not fully verified. **SQ3 reference standard:** The reference standard appears clinical/pathological, but its conduct and blinded interpretation were incompletely reported. **SQ4 flow/timing:** Complete participant flow, interval, and inclusion in analysis were not fully reported.	**App PS:** Low concern; the studied ovarian cancer population was relevant to the review question. **App IT:** Low concern; circulating miRNA measurement was relevant. **App RS:** Low concern; the clinical/pathological diagnosis addressed the target condition.
A19	Hong et al. (2013) [84]	Diagnostic: Correct classification: 88%; stage classification: 77%; grade classification: 76%; prognostic: High miR-221 independently predicts worse OS	**SQ1 selection:** Separately selected cancer and healthy/control groups create a two-gate or case-control spectrum. **SQ2 index test:** Objective assay/performance analysis was reported, but prespecification and blinded interpretation were not fully verified. **SQ3 reference standard:** Histopathological diagnosis was used as the reference standard. **SQ4 flow/timing:** Complete participant flow, interval, and inclusion in analysis were not fully reported.	**App PS:** Low concern; the population was relevant to the intended review population. **App IT:** Low concern; the circulating miRNA assessment matched the review question. **App RS:** Low concern; histopathological diagnosis was appropriate.
A20	Meng et al. (2016) [39]	Diagnostic: miR-200a AUC=0.914; miR-200b AUC=0.815; combined panel AUC=0.925, Se 88%, Sp 90%; prognostic: miR-200b/c associated with poor OS and DFS; independent prognostic factors	**SQ1 selection:** Separately selected cancer and healthy/control groups create a two-gate or case-control spectrum. **SQ2 index test:** The cut-off/threshold was optimized in the analysed data. **SQ3 reference standard:** Histopathological diagnosis was used as the reference standard. **SQ4 flow/timing:** Complete participant flow, interval, and inclusion in analysis were not fully reported.	**App PS:** Low concern; the studied population was relevant to the review question. **App IT:** Low concern; the circulating miRNA panel was relevant. **App RS:** Low concern; histopathological diagnosis was appropriate.

Entry	Study [reference]	Target result or model	Recorded rationale	Applicability rationale
A21	Minareci et al. (2024) [92]	Diagnostic: miR-4314 AUC=0.78; miR-1181 AUC=0.76; combined AUC=0.81; prognostic: High expression linked to worse OS and DFS	**SQ1 selection:** Consecutive/random enrolment and exclusions could not be verified. **SQ2 index test:** Objective assay/performance analysis was reported, but prespecification and blinded interpretation were not fully verified. **SQ3 reference standard:** The reference standard appears clinical/pathological, but its conduct and blinded interpretation were incompletely reported. **SQ4 flow/timing:** Complete participant flow, interval, and inclusion in analysis were not fully reported.	**App PS:** Low concern; the patient population was considered relevant to the review question. **App IT:** Low concern; circulating miRNA testing matched the review scope. **App RS:** Low concern; the clinical/pathological diagnosis addressed the target condition.
A22	Song et al. (2020) [87]	Diagnostic: miR-26b AUC=0.753; miR-21 AUC=0.826; combined AUC=0.905 in Table III (0.916 in source narrative; unresolved discrepancy); prognostic: miR-26b linked to better survival; miR-21 linked to worse survival	**SQ1 selection:** Consecutive/random enrolment and exclusions could not be verified. **SQ2 index test:** Objective assay/performance analysis was reported, but prespecification and blinded interpretation were not fully verified. **SQ3 reference standard:** The reference standard appears clinical/pathological, but its conduct and blinded interpretation were incompletely reported. **SQ4 flow/timing:** Complete participant flow, interval, and inclusion in analysis were not fully reported.	**App PS:** Low concern; the evaluated population was relevant to the review question. **App IT:** Low concern; the circulating miRNA biomarkers were relevant. **App RS:** Low concern; the clinical/pathological reference diagnosis corresponded to ovarian cancer.
A23	Zhang et al. (2020) [81]	Diagnostic: miR-484: AUC=0.821, Se 77.9%, Sp 80%; combined with CA125: AUC=0.912; prognostic: Low miR-484 linked to poor OS/PFS and aggressive disease	**SQ1 selection:** Consecutive/random enrolment and exclusions could not be verified. **SQ2 index test:** Objective assay/performance analysis was reported, but prespecification and blinded interpretation were not fully verified. **SQ3 reference standard:** The reference standard appears clinical/pathological, but its conduct and blinded interpretation were incompletely reported. **SQ4 flow/timing:** Complete participant flow, interval, and inclusion in analysis were not fully reported.	**App PS:** Low concern; the patient population was applicable to the review question. **App IT:** Low concern; circulating miRNA testing was relevant. **App RS:** Low concern; the clinical/pathological reference diagnosis addressed the target condition.
A24	Zhao et al. (2018) [79]	Diagnostic: AUC=0.879; Se 72.9%; Sp 98.0%; prognostic: High miR-106a linked to advanced stage and poor OS	**SQ1 selection:** Consecutive/random enrolment and exclusions could not be verified. **SQ2 index test:** Objective assay/performance analysis was reported, but prespecification and blinded interpretation were not fully verified. **SQ3 reference standard:** The reference standard appears clinical/pathological, but its conduct and blinded interpretation were incompletely reported. **SQ4 flow/timing:** Complete participant flow, interval, and inclusion in analysis were not fully reported.	**App PS:** Low concern; the studied population was relevant to the review question. **App IT:** Low concern; the circulating miRNA biomarker was relevant. **App RS:** Low concern; the clinical/pathological diagnosis corresponded to ovarian cancer.

Entry	Study [reference]	Target result or model	Recorded rationale	Applicability rationale
A25	Zhu et al. (2017) [86]	Diagnostic: AUC=0.737; Se 75.6%; Sp 68.5%; prognostic: Higher miR-125b linked to longer PFS and better OS trend	**SQ1 selection:** Consecutive/random enrolment and exclusions could not be verified. **SQ2 index test:** Objective assay/performance analysis was reported, but prespecification and blinded interpretation were not fully verified. **SQ3 reference standard:** The reference standard appears clinical/pathological, but its conduct and blinded interpretation were incompletely reported. **SQ4 flow/timing:** Complete participant flow, interval, and inclusion in analysis were not fully reported.	**App PS:** Low concern; the patient population was considered applicable to the review question. **App IT:** Low concern; circulating miRNA measurement was relevant. **App RS:** Low concern; the clinical/pathological diagnosis addressed the target condition.
A26	Zuberi et al. (2016) [42]	Diagnostic: AUC=0.704; Se 69.1%; Sp 95.7%; prognostic: Low miR-199a linked to poor OS and metastasis	**SQ1 selection:** Consecutive/random enrolment and exclusions could not be verified. **SQ2 index test:** Objective assay/performance analysis was reported, but prespecification and blinded interpretation were not fully verified. **SQ3 reference standard:** Histopathological diagnosis was used as the reference standard. **SQ4 flow/timing:** Complete participant flow, interval, and inclusion in analysis were not fully reported.	**App PS:** Low concern; the studied population was relevant to the review question. **App IT:** Low concern; circulating miRNA testing matched the review scope. **App RS:** Low concern; histopathological diagnosis was appropriate.
A27	Zuberi et al. (2020) [41]	Diagnostic: AUC=0.928; Se 69.1%; Sp 95.7%; prognostic: Low miR-145 linked to advanced disease and poorer survival	**SQ1 selection:** Consecutive/random enrolment and exclusions could not be verified. **SQ2 index test:** Objective assay/performance analysis was reported, but prespecification and blinded interpretation were not fully verified. **SQ3 reference standard:** Histopathological diagnosis was used as the reference standard. **SQ4 flow/timing:** Complete participant flow, interval, and inclusion in analysis were not fully reported.	**App PS:** Low concern; the studied population was relevant to the review question. **App IT:** Low concern; circulating miRNA testing was relevant. **App RS:** Low concern; histopathological diagnosis was appropriate.
A28	Zuberi et al. (2016) [19]	Serum miR-125b, EOC versus healthy controls; AUC 0.728.	**Patient selection:** separately selected cases and healthy controls (two-gate design). **Index test:** cutoff 3.76 selected/evaluated in the same dataset, no independent validation. **Reference standard:** verification and blinding insufficiently described. **Flow/timing:** complete diagnostic participant flow and index/reference interval not established.	Healthy controls limit applicability to adnexal-mass triage. Serum assay is relevant; reference-standard applicability uncertain.
A29	Liu et al. (2021) [62]	Plasma exosomal miR-4732-5p; OC versus healthy controls. Validation: 28 OC/17 controls; AUC 0.889 (source Figure 4d).	**Patient selection:** high risk because cancer cases and healthy controls were selected separately. Index test: threshold prespecification and blinding were not reported. **Reference standard:** pathological diagnoses were listed, but reference-standard conduct and blinding were incompletely described**.** **Flow/timing:** collection was preoperative, but the index/reference interval and full participant flow were not adequately reported; unclear.	**Patient-selection applicability:** high concern for routine adnexal-mass evaluation because controls were healthy. Index-test and reference-standard applicability: low concern for the review question.

Entry	Study [reference]	Target result or model	Recorded rationale	Applicability rationale
A30	Günel et al. (2018) [21]	Serum hsa-miR-1273g-3p; established recurrent EOC versus healthy controls.	**Patient selection high:** selected recurrent cases and healthy controls. Index test high: marker/threshold selection and evaluation without established independent validation. Reference standard unclear: clinical recurrence verification and blinding insufficiently described. Flow/timing unclear: phase overlap and participant exclusions not fully traceable.	Healthy controls do not represent the clinical differential diagnosis of suspected recurrence; high patient-selection concern. Relevant serum assay; reference-standard applicability unclear.
A31	Kumari et al. (2022) [24]	Serum miR-145; six candidate miRNAs assayed; miR-145 AUC 0.790 (95% CI 0.653–0.927); source sensitivity 90%, specificity 73% (Table IV).	**Patient selection high:** selected cancer and noncancer groups rather than an unselected clinical diagnostic series. Index test high: candidate/threshold selection and evaluation in the same small cohort, without independent validation. Reference standard low: histopathological confirmation reported. Flow/timing unclear: complete participant flow and index-to-reference timing insufficiently reported.	High patient-selection applicability concern because selected controls do not represent the full clinical differential diagnosis. Low index-test concern; low reference-standard concern.
A32	Meng et al. (2016) [25]	Cell-free miR-373 and miR-200a/b/c; three-miR-200 panel; miR-200a/b/c AUCs 0.853/0.953/0.903; combined panel 0.959 (Figure 1.1).	**Patient selection high:** selected cancer and noncancer groups rather than an unselected clinical diagnostic series. Index test high: candidate/threshold selection and evaluation in the same small cohort, without independent validation. Reference standard unclear: verification/blinding insufficiently described for all analysed groups. Flow/timing unclear: complete participant flow and index-to-reference timing insufficiently reported.	High patient-selection applicability concern because selected controls do not represent the full clinical differential diagnosis. Low index-test concern; unclear reference-standard concern.
A33	Tian et al. (2025) [29]	miR-338-3p; CMD model combining it with three circRNAs; miR-338-3p AUC 0.905 overall and 0.600 early-stage; CMD AUC 0.9789 overall and 1.000 early-stage (Figures 8B,C).	**Patient selection high:** selected cancer and noncancer groups rather than an unselected clinical diagnostic series. Index test high: candidate/threshold selection and evaluation in the same small cohort, without independent validation. Reference standard low: histopathological confirmation reported. Flow/timing unclear: complete participant flow and index-to-reference timing insufficiently reported.	High patient-selection applicability concern because selected controls do not represent the full clinical differential diagnosis. Low index-test concern; low reference-standard concern.
A34	Günel et al. (2019) [35]	Circulating hsa-let-7d-3p; AUC 0.700 (95% CI 0.4595–0.9405); no independently validated threshold performance.	**Patient selection high:** selected cancer and noncancer groups rather than an unselected clinical diagnostic series. Index test high: candidate/threshold selection and evaluation in the same small cohort, without independent validation. Reference standard unclear: verification/blinding insufficiently described for all analysed groups. Flow/timing unclear: complete participant flow and index-to-reference timing insufficiently reported.	High patient-selection applicability concern because selected controls do not represent the full clinical differential diagnosis. Low index-test concern; unclear reference-standard concern.

Part B QUIPS

Six QUIPS risk-of-bias domains; no overall judgment is assigned.

L, low; M, moderate; H, high.

Entry	Study [reference]	Participation	Attrition	Factor measurement	Outcome measurement	Confounding	Analysis and reporting
B01	Benson et al. (2015) [76]	M	H	M	L	H	H
B02	Chen et al. (2016) [69]	M	M	M	L	M	M
B03	Fukagawa et al. (2017) [43]	M	M	M	L	H	H
B04	Gong et al. (2016) [70]	M	M	M	L	M	M
B05	Halvorsen et al. (2017) [77]	L	M	L	L	M	M
B06	Kapetanakis et al. (2015) [73]	M	M	M	L	M	M
B07	Pal et al. (2024) [72]	M	M	M	L	H	H
B08	Robelin et al. (2020) [75]	L	M	L	L	M	M
B09	Yoshida et al. (2021) [12]	M	M	M	L	M	M
B10	Zhong et al. (2025) [71]	M	M	M	L	H	H
B11	Chen et al. (2020) [85]	M	M	M	L	H	H
B12	Gahlawat et al. (2022) [9]	M	M	M	L	M	M
B13	Gao & Wu (2015) [89]	M	M	M	L	H	H
B14	Helmy et al. (2025) [80]	M	M	M	L	H	H
B15	Hong et al. (2013) [84]	M	M	M	L	M	M
B16	Li et al. (2023) [83]	M	M	M	L	M	M
B17	Maeda et al. (2020) [88]	M	M	M	L	H	H
B18	Meng et al. (2015) [40]	M	M	M	L	H	H
B19	Meng et al. (2016) [39]	M	M	M	L	M	M
B20	Minareci et al. (2024) [92]	M	M	M	L	H	H
B21	Ranmale et al. (2025) [93]	M	M	M	L	M	M

Entry	Study [reference]	Participation	Attrition	Factor measurement	Outcome measurement	Confounding	Analysis and reporting
B22	Shah et al. (2018) [82]	M	M	M	L	H	H
B23	Song et al. (2020) [87]	M	M	M	L	H	H
B24	Zhang et al. (2020) [81]	M	M	M	L	H	H
B25	Zhao et al. (2018) [79]	M	M	M	L	H	H
B26	Zheng et al. (2013) [95]	M	M	M	L	H	H
B27	Zhu et al. (2017) [86]	M	M	M	L	H	H
B28	Zuberi et al. (2016) [42]	M	M	M	L	H	H
B29	Zuberi et al. (2020) [41]	M	M	M	L	H	H
B30	Xu et al. (2013) [18]	M	M	M	L	H	H
B31	Zuberi et al. (2016) [19]	M	M	M	M	H	H
B32	Shapira et al. (2014) [23]	H	M	M	L	H	H
B33	Sun et al. (2024) [30]	H	M	M	M	H	H

Part B Supporting assessment details

Entry	Study [reference]	Target result or model	Recorded rationale
B01	Benson et al. (2015) [76]	**Outcomes:** Pre-/post-chemotherapy changes, treatment response, PFS; analysis: Paired & two-tailed t-tests, Spearman correlation, Kaplan–Meier, log-rank	**Participation:** representativeness partly uncertain. **Attrition:** important exclusions/very small evaluable cohort. **Factor measurement:** assay reported; blinding, reproducibility, normalization or cut-point prespecification partly uncertain. **Outcome:** objective time-to-event outcome. **Confounding:** adequate adjustment for key clinicopathological factors not demonstrated. **Analysis:** mainly unadjusted/data-derived analysis.
B02	Chen et al. (2016) [69]	**Outcomes:** OS, clinicopathological associations, proliferation, drug sensitivity; analysis: t-test, ANOVA, Chi-square, Kaplan–Meier, log-rank, Cox regression	**Participation:** representativeness partly uncertain. **Attrition:** losses and missing prognostic data incompletely reported. **Factor measurement:** assay reported; blinding, reproducibility, normalization or cut-point prespecification partly uncertain. **Outcome:** objective time-to-event outcome. **Confounding:** multivariable adjustment reported, residual confounding possible. **Analysis:** structured survival/multivariable analysis with remaining small-sample concerns.

Entry	Study [reference]	Target result or model	Recorded rationale
B03	Fukagawa et al. (2017) [43]	**Outcomes:** PFS, chemosensitivity, tumour growth; analysis: Mann–Whitney U, Kaplan–Meier, log-rank, Pearson correlation	**Participation:** representativeness partly uncertain. **Attrition:** losses and missing prognostic data incompletely reported. **Factor measurement:** assay reported; blinding, reproducibility, normalization or cut-point prespecification partly uncertain. **Outcome:** objective time-to-event outcome. **Confounding:** adequate adjustment for key clinicopathological factors not demonstrated. **Analysis:** mainly unadjusted/data-derived analysis.
B04	Gong et al. (2016) [70]	**Outcomes:** OS, clinicopathological features, proliferation, migration, invasion; analysis: t-test, Chi-square, Kaplan–Meier, log-rank, Cox regression	**Participation:** representativeness partly uncertain. **Attrition:** losses and missing prognostic data incompletely reported. **Factor measurement:** assay reported; blinding, reproducibility, normalization or cut-point prespecification partly uncertain. **Outcome:** objective time-to-event outcome. **Confounding:** multivariable adjustment reported, residual confounding possible. **Analysis:** structured survival/multivariable analysis with remaining small-sample concerns.
B05	Halvorsen et al. (2017) [77]	**Outcomes:** PFS, OS, treatment response (bevacizumab); analysis: SAM analysis, t-test, Kaplan–Meier, log-rank, multivariate Cox regression	**Participation:** prospective/trial cohort. **Attrition:** losses and missing prognostic data incompletely reported. **Factor measurement:** defined longitudinal schedule. **Outcome:** objective time-to-event outcome. **Confounding:** multivariable adjustment reported, residual confounding possible. **Analysis:** structured survival/multivariable analysis with remaining small-sample concerns.
B06	Kapetanakis et al. (2015) [73]	**Outcomes:** Diagnostic discrimination, longitudinal changes, PFS; analysis: Kruskal–Wallis, linear regression, Kaplan–Meier, log-rank, Cox models, landmark analysis	**Participation:** representativeness partly uncertain. **Attrition:** losses and missing prognostic data incompletely reported. **Factor measurement:** assay reported; blinding, reproducibility, normalization or cut-point prespecification partly uncertain. **Outcome:** objective time-to-event outcome. **Confounding:** multivariable adjustment reported, residual confounding possible. **Analysis:** structured survival/multivariable analysis with remaining small-sample concerns.
B07	Pal et al. (2024) [72]	Individual circulating miRNA associations with OS and chemotherapy-response groups; no combined five-miRNA score established.	**Participation and attrition:** single clinical cohort with incomplete information on follow-up losses. Factor measurement: assays reported, but cut-point prespecification and blinding unclear. Outcome: overall survival. Confounding: the source labels Cox results multivariable but does not specify the covariates; high risk. Analysis: small response subgroups, multiple marker comparisons, nonsignificant adjusted estimates and no independent validation; high risk.
B08	Robelin et al. (2020) [75]	**Outcomes:** RECIST response, IDS completeness, PFS, OS, longitudinal kinetics; analysis: ROC analysis, Wilcoxon signed-rank, Kaplan–Meier, log-rank, c-index, multivariate modelling	**Participation:** prospective/trial cohort. **Attrition:** losses and missing prognostic data incompletely reported. **Factor measurement:** defined longitudinal schedule. **Outcome:** objective time-to-event outcome. **Confounding:** multivariable adjustment reported, residual confounding possible. **Analysis:** structured survival/multivariable analysis with remaining small-sample concerns.
B09	Yoshida et al. (2021) [12]	**Outcomes:** OS, PFS; analysis: Kaplan–Meier, log-rank, univariate/multivariate Cox regression, prognostic index modelling	**Participation:** representativeness partly uncertain. **Attrition:** losses and missing prognostic data incompletely reported. **Factor measurement:** assay reported; blinding, reproducibility, normalization or cut-point prespecification partly uncertain. **Outcome:** objective time-to-event outcome. **Confounding:** multivariable adjustment reported, residual confounding possible. **Analysis:** structured survival/multivariable analysis with remaining small-sample concerns.

Entry	Study [reference]	Target result or model	Recorded rationale
B10	Zhong et al. (2025) [71]	**Outcomes:** Chemotherapy response, survival, prognostic risk factors; analysis: Chi-square, t-test, logistic regression, ROC analysis	**Participation:** representativeness partly uncertain. Attrition: losses and missing prognostic data incompletely reported. Factor measurement: assay reported; blinding, reproducibility, normalization or cut-point prespecification partly uncertain. Outcome: binary alive/deceased status at three years, not a time-to-event analysis; the source does not clearly define regression outcome/reference coding. Confounding: adequate adjustment for key clinicopathological factors not demonstrated. Analysis: mainly unadjusted/data-derived analysis.
B11	Chen et al. (2020) [85]	Diagnostic: miR-125b: AUC≈0.73, Se1 76%, Sp2 41.6%; combined with CA125: AUC≈0.90, Se 83.6%, Sp 95.6%; prognostic: Low miR-125b linked to advanced stage, LN metastasis, chemoresistance, poorer prognosis; higher post-surgery	**Participation:** representativeness partly uncertain. **Attrition:** losses and missing prognostic data incompletely reported. **Factor measurement:** assay reported; blinding, reproducibility, normalization or cut-point prespecification partly uncertain. **Outcome:** objective time-to-event outcome. **Confounding:** adequate adjustment for key clinicopathological factors not demonstrated. **Analysis:** mainly unadjusted/data-derived analysis.
B12	Gahlawat et al. (2022) [9]	Diagnostic: Panel: AUC=0.87; benign vs control AUC=0.77; early-stage AUC=0.81; combined with CA125: AUC=0.97 overall, 0.93 early-stage; prognostic: High cf-miRNA associated with worse OS; independent prognostic marker (HR≈1.83); no PFS association	**Participation:** representativeness partly uncertain. **Attrition:** losses and missing prognostic data incompletely reported. **Factor measurement:** assay reported; blinding, reproducibility, normalization or cut-point prespecification partly uncertain. **Outcome:** objective time-to-event outcome. **Confounding:** multivariable adjustment reported, residual confounding possible. **Analysis:** structured survival/multivariable analysis with remaining small-sample concerns.
B13	Gao & Wu (2015) [89]	Diagnostic: miR-200c: AUC=0.79, Se 72%, Sp 70%; miR-141: AUC=0.75, Se 69%, Sp 72%; prognostic: High expression associated with shorter survival; miR-200c stronger prognostic marker	**Participation:** representativeness partly uncertain. **Attrition:** losses and missing prognostic data incompletely reported. **Factor measurement:** assay reported; blinding, reproducibility, normalization or cut-point prespecification partly uncertain. **Outcome:** objective time-to-event outcome. **Confounding:** adequate adjustment for key clinicopathological factors not demonstrated. **Analysis:** mainly unadjusted/data-derived analysis.
B14	Helmy et al. (2025) [80]	Diagnostic: miR-200b: AUC=0.743, Se 63.3%, Sp 71%; miR-200b+CA125: Se 100%, Sp 93.1%; prognostic: High miR-200b linked to shorter EFS	**Participation:** representativeness partly uncertain. **Attrition:** losses and missing prognostic data incompletely reported. **Factor measurement:** assay reported; blinding, reproducibility, normalization or cut-point prespecification partly uncertain. **Outcome:** objective time-to-event outcome. **Confounding:** adequate adjustment for key clinicopathological factors not demonstrated. **Analysis:** mainly unadjusted/data-derived analysis.
B15	Hong et al. (2013) [84]	Diagnostic: Correct classification: 88%; stage classification: 77%; grade classification: 76%; prognostic: High miR-221 independently predicts worse OS	**Participation:** representativeness partly uncertain. **Attrition:** losses and missing prognostic data incompletely reported. **Factor measurement:** assay reported; blinding, reproducibility, normalization or cut-point prespecification partly uncertain. **Outcome:** objective time-to-event outcome. **Confounding:** multivariable adjustment reported, residual confounding possible. **Analysis:** structured survival/multivariable analysis with remaining small-sample concerns.

Entry	Study [reference]	Target result or model	Recorded rationale
B16	Li et al. (2023) [83]	Diagnostic: Training AUC=0.913; testing AUC=0.973; validation AUC=0.924; early-stage AUC=0.903; prognostic: High miR-141-3p and miR-200c-3p linked to poor OS; RiskScore predicts PFS	**Participation:** representativeness partly uncertain. **Attrition:** losses and missing prognostic data incompletely reported. **Factor measurement:** assay reported; blinding, reproducibility, normalization or cut-point prespecification partly uncertain. **Outcome:** objective time-to-event outcome. **Confounding:** multivariable adjustment reported, residual confounding possible. **Analysis:** structured survival/multivariable analysis with remaining small-sample concerns.
B17	Maeda et al. (2020) [88]	Diagnostic: Expression differences only; no ROC/AUC; prognostic: Low miR-34a linked to LN metastasis and recurrence	**Participation:** representativeness partly uncertain. **Attrition:** losses and missing prognostic data incompletely reported. **Factor measurement:** assay reported; blinding, reproducibility, normalization or cut-point prespecification partly uncertain. **Outcome:** objective time-to-event outcome. **Confounding:** adequate adjustment for key clinicopathological factors not demonstrated. **Analysis:** mainly unadjusted/data-derived analysis.
B18	Meng et al. (2015) [40]	Diagnostic: Se 93%; Sp 92%; prognostic: miR-429 predicts OS; miR-7 linked to metastasis; miR-376a linked to advanced stage	**Participation:** representativeness partly uncertain. **Attrition:** losses and missing prognostic data incompletely reported. **Factor measurement:** assay reported; blinding, reproducibility, normalization or cut-point prespecification partly uncertain. **Outcome:** objective time-to-event outcome. **Confounding:** adequate adjustment for key clinicopathological factors not demonstrated. **Analysis:** mainly unadjusted/data-derived analysis.
B19	Meng et al. (2016) [39]	Diagnostic: miR-200a AUC=0.914; miR-200b AUC=0.815; combined panel AUC=0.925, Se 88%, Sp 90%; prognostic: miR-200b/c associated with poor OS and DFS; independent prognostic factors	**Participation:** representativeness partly uncertain. **Attrition:** losses and missing prognostic data incompletely reported. **Factor measurement:** assay reported; blinding, reproducibility, normalization or cut-point prespecification partly uncertain. **Outcome:** objective time-to-event outcome. **Confounding:** multivariable adjustment reported, residual confounding possible. **Analysis:** structured survival/multivariable analysis with remaining small-sample concerns.
B20	Minareci et al. (2024) [92]	Diagnostic: miR-4314 AUC=0.78; miR-1181 AUC=0.76; combined AUC=0.81; prognostic: High expression linked to worse OS and DFS	**Participation:** representativeness partly uncertain. **Attrition:** losses and missing prognostic data incompletely reported. **Factor measurement:** assay reported; blinding, reproducibility, normalization or cut-point prespecification partly uncertain. **Outcome:** objective time-to-event outcome. **Confounding:** adequate adjustment for key clinicopathological factors not demonstrated. **Analysis:** mainly unadjusted/data-derived analysis.
B21	Ranmale et al. (2025) [93]	Diagnostic: Combined panel: AUC=0.743, Se 81.6%, Sp 60.5%; prognostic: serum miR-130a associated with PFS; tissue miR-20a excluded from circulating evidence; several miRNAs linked to relapse and chemo response	**Participation:** representativeness partly uncertain. **Attrition:** losses and missing prognostic data incompletely reported. **Factor measurement:** assay reported; blinding, reproducibility, normalization or cut-point prespecification partly uncertain. **Outcome:** objective time-to-event outcome. **Confounding:** multivariable adjustment reported, residual confounding possible. **Analysis:** structured survival/multivariable analysis with remaining small-sample concerns.

Entry	Study [reference]	Target result or model	Recorded rationale
B22	Shah et al. (2018) [82]	Association of circulating miR-34a-5p with surgical cytoreduction outcome; diagnostic CA-125/miRNA combinations are assessed separately under PROBAST.	**Participation:** representativeness partly uncertain. Attrition: losses and missing prognostic data incompletely reported. Factor measurement: assay reported; blinding, reproducibility, normalization or cut-point prespecification partly uncertain. Outcome: residual disease after cytoreductive surgery, not survival; assessment blinding was incompletely reported. Confounding: adequate adjustment for key clinicopathological factors not demonstrated. Analysis: mainly unadjusted/data-derived analysis.
B23	Song et al. (2020) [87]	Diagnostic: miR-26b AUC=0.753; miR-21 AUC=0.826; combined AUC=0.905 in Table III (0.916 in source narrative; unresolved discrepancy); prognostic: miR-26b linked to better survival; miR-21 linked to worse survival	**Participation:** representativeness partly uncertain. **Attrition:** losses and missing prognostic data incompletely reported. **Factor measurement:** assay reported; blinding, reproducibility, normalization or cut-point prespecification partly uncertain. **Outcome:** objective time-to-event outcome. **Confounding:** adequate adjustment for key clinicopathological factors not demonstrated. **Analysis:** mainly unadjusted/data-derived analysis.
B24	Zhang et al. (2020) [81]	Diagnostic: miR-484: AUC=0.821, Se 77.9%, Sp 80%; combined with CA125: AUC=0.912; prognostic: Low miR-484 linked to poor OS/PFS and aggressive disease	**Participation:** representativeness partly uncertain. **Attrition:** losses and missing prognostic data incompletely reported. **Factor measurement:** assay reported; blinding, reproducibility, normalization or cut-point prespecification partly uncertain. **Outcome:** objective time-to-event outcome. **Confounding:** adequate adjustment for key clinicopathological factors not demonstrated. **Analysis:** mainly unadjusted/data-derived analysis.
B25	Zhao et al. (2018) [79]	Diagnostic: AUC=0.879; Se 72.9%; Sp 98.0%; prognostic: High miR-106a linked to advanced stage and poor OS	**Participation:** representativeness partly uncertain. **Attrition:** losses and missing prognostic data incompletely reported. **Factor measurement:** assay reported; blinding, reproducibility, normalization or cut-point prespecification partly uncertain. **Outcome:** objective time-to-event outcome. **Confounding:** adequate adjustment for key clinicopathological factors not demonstrated. **Analysis:** mainly unadjusted/data-derived analysis.
B26	Zheng et al. (2013) [95]	Diagnostic: High diagnostic accuracy; improved with CA125; prognostic: Low let-7f linked to poor prognosis	**Participation:** representativeness partly uncertain. **Attrition:** losses and missing prognostic data incompletely reported. **Factor measurement:** assay reported; blinding, reproducibility, normalization or cut-point prespecification partly uncertain. **Outcome:** objective time-to-event outcome. **Confounding:** adequate adjustment for key clinicopathological factors not demonstrated. **Analysis:** mainly unadjusted/data-derived analysis.
B27	Zhu et al. (2017) [86]	Diagnostic: AUC=0.737; Se 75.6%; Sp 68.5%; prognostic: Higher miR-125b linked to longer PFS and better OS trend	**Participation:** representativeness partly uncertain. **Attrition:** losses and missing prognostic data incompletely reported. **Factor measurement:** assay reported; blinding, reproducibility, normalization or cut-point prespecification partly uncertain. **Outcome:** objective time-to-event outcome. **Confounding:** adequate adjustment for key clinicopathological factors not demonstrated. **Analysis:** mainly unadjusted/data-derived analysis.

Entry	Study [reference]	Target result or model	Recorded rationale
B28	Zuberi et al. (2016) [42]	Diagnostic: AUC=0.704; Se 69.1%; Sp 95.7%; prognostic: Low miR-199a linked to poor OS and metastasis	**Participation:** representativeness partly uncertain. **Attrition:** losses and missing prognostic data incompletely reported. **Factor measurement:** assay reported; blinding, reproducibility, normalization or cut-point prespecification partly uncertain. **Outcome:** objective time-to-event outcome. **Confounding:** adequate adjustment for key clinicopathological factors not demonstrated. **Analysis:** mainly unadjusted/data-derived analysis.
B29	Zuberi et al. (2020) [41]	Diagnostic: AUC=0.928; Se 69.1%; Sp 95.7%; prognostic: Low miR-145 linked to advanced disease and poorer survival	**Participation:** representativeness partly uncertain. **Attrition:** losses and missing prognostic data incompletely reported. **Factor measurement:** assay reported; blinding, reproducibility, normalization or cut-point prespecification partly uncertain. **Outcome:** objective time-to-event outcome. **Confounding:** adequate adjustment for key clinicopathological factors not demonstrated. **Analysis:** mainly unadjusted/data-derived analysis.
B30	Xu et al. (2013) [18]	Serum miR-21 and overall survival.	Retrospective single-centre participation; completeness of prognostic follow-up not fully described. U6-normalized qPCR; miR-21/miR-221 labelling inconsistencies. OS is objective. Residual disease and treatment confounding not adequately addressed. HR direction for ΔCt conflicts with narrative; confidence interval absent.
B31	Zuberi et al. (2016) [19]	Serum miR-125b and overall survival (null association).	Single-centre participation and incompletely described prognostic follow-up. U6-normalized qPCR; cut point not independently validated. Survival follow-up and ascertainment insufficiently described. No adjusted survival model; limited reporting of time-to-event analysis.
B32	Shapira et al. (2014) [23]	Survival-group association.	**Participation high:** selected outcome-defined groups. Attrition moderate: selection/missingness in the clinical outcome subset incompletely explained. Factor measurement moderate: assay reported but blinding and prespecified clinical cutpoints insufficiently established. Outcome low: survival status and follow-up define comparison groups. Confounding high: no adequate adjustment for established clinical prognostic factors. Analysis high: small same-cohort, unadjusted/data-selected comparisons without validation.
B33	Sun et al. (2024) [30]	Plasma miR-296-3p association with paclitaxel response.	**Participation high:** selected outcome-defined groups. Attrition moderate: selection/missingness in the clinical outcome subset incompletely explained. Factor measurement moderate: assay reported but blinding and prespecified clinical cutpoints insufficiently established. Outcome moderate: clinical sensitive/resistant groups reported, but reproducible response adjudication incompletely specified. Confounding high: no adequate adjustment for established clinical prognostic factors. Analysis high: small same-cohort, unadjusted/data-selected comparisons without validation.

Part C PROBAST models

P, participants; Pr, predictors; O, outcome; A, analysis; App, applicability concerns.

Entry	Study [reference]	P	Pr	O	A	Overall	App P	App Pr	App O	App overall
C01	Ali et al. (2022) [58]	H	U	L	H	H	U	L	L	U
C02	Choi et al. (2020) [49]	H	U	L	H	H	U	L	L	U
C03	Cirillo et al. (2021) [2]	H	U	L	H	H	U	L	L	U
C04	Elias et al. (2017) [52]	H	L	L	U	H	U	L	L	U
C05	Gockley et al. (2022) [51]	H	L	L	U	H	U	L	L	U
C06	Hamidi et al. (2021) [55]	U	L	L	U	U	U	L	L	U
C07	Hong et al. (2022) [54]	U	U	L	H	H	H	L	L	H
C08	Kan et al. (2012) [59]	H	U	L	H	H	U	L	L	U
C09	Kandimalla et al. (2021) [14]	H	L	L	U	H	U	L	L	U
C10	Kumar et al. (2021) [13]	H	U	L	H	H	U	L	L	U
C11	Kumar et al. (2022) [36]	H	U	L	H	H	U	L	L	U
C12	Liang et al. (2020) [5]	H	U	L	H	H	L	L	L	L
C13	Niemira et al. (2023) [11]	H	L	L	U	H	H	L	L	H
C14	Oliveira et al. (2019) [10]	U	U	L	H	H	L	L	L	L
C15	Ren et al. (2018) [3]	H	U	L	H	H	L	L	L	L
C16	Stawiski et al. (2024) [65]	L	L	L	U	U	H	L	L	H
C17	Su et al. (2019) [6]	H	U	L	H	H	L	L	L	L
C18	Wang et al. (2019a) [37]	H	U	L	H	H	U	L	L	U
C19	Wang et al. (2022) [53]	U	L	L	U	U	U	L	L	U
C20	Webber et al. (2024) [4]	L	L	L	U	U	L	L	L	L
C21	Wiczling et al. (2019) [57]	H	U	L	H	H	U	L	L	U
C22	Yang et al. (2024) [56]	H	U	L	H	H	L	L	L	L
C23	Yokoi et al. (2017) [50]	U	U	L	H	H	L	L	L	L

Entry	Study [reference]	P	Pr	O	A	Overall	App P	App Pr	App O	App overall
C24	Yokoi et al. (2018) [8]	H	L	L	U	H	L	L	L	L
C25	Zhang & Hu (2022) [68]	H	U	L	H	H	H	L	L	H
C26	Zhu et al. (2022) [47]	H	U	L	H	H	L	L	L	L
C29	Uehara et al. (2024) [78]	U	U	U	H	H	H	L	U	H
C30	Yoshida et al. (2021) [12]	U	L	L	U	U	U	L	L	U
C31	Zhong et al. (2025) [71]	U	U	U	H	H	U	L	U	U
C32	Chen et al. (2020) [85]	U	U	L	H	H	U	L	L	U
C33	Gahlawat et al. (2022) [9]	U	U	L	H	H	U	L	L	U
C34	Helmy et al. (2025) [80]	U	U	L	H	H	U	L	L	U
C35	Li et al. (2023) [83]	U	L	L	U	U	L	L	L	L
C36	Li et al. (2023) [83]	U	L	L	U	U	L	L	L	L
C37	Meng et al. (2015) [40]	U	U	L	H	H	U	L	L	U
C38	Meng et al. (2016) [39]	U	U	L	H	H	U	L	L	U
C39	Minareci et al. (2024) [92]	U	U	L	H	H	U	L	L	U
C40	Ranmale et al. (2025) [93]	U	U	L	H	H	L	L	L	L
C41	Shah et al. (2018) [82]	U	U	U	H	H	L	L	U	U
C42	Song et al. (2020) [87]	U	U	L	H	H	U	L	L	U
C43	Wang et al. (2019b) [38]	U	U	L	H	H	U	L	L	U
C44	Zhang et al. (2020) [81]	U	U	L	H	H	U	L	L	U
C45	Zheng et al. (2013) [95]	U	U	L	H	H	L	L	L	L
C46	Zhou et al. (2023) [20]	H	U	U	H	H	L	L	L	L
C47	Zhou et al. (2023) [20]	H	U	U	H	H	L	L	L	L
C48	Suryawanshi et al. (2013) [22]	H	U	L	H	H	H	L	L	H
C49	Suryawanshi et al. (2013) [22]	H	U	L	H	H	H	L	L	H
C50	Suryawanshi et al. (2013) [22]	H	U	L	H	H	H	L	L	H

Entry	Study [reference]	P	Pr	O	A	Overall	App P	App Pr	App O	App overall
C51	Suryawanshi et al. (2013) [22]	H	U	L	H	H	H	L	L	H
C52	Meng et al. (2016) [25]	H	U	U	H	H	H	L	L	H
C53	Tian et al. (2025) [29]	H	U	L	H	H	H	L	L	H
C54	Timofeeva et al. (2023) [31]	U	U	L	H	H	L	L	L	L
C55	Timofeeva et al. (2023) [31]	U	U	L	H	H	L	L	L	L
C56	Timofeeva et al. (2023) [31]	U	U	L	H	H	L	L	L	L
C57	Timofeeva et al. (2023) [31]	U	U	L	H	H	L	L	L	L
C58	Timofeeva et al. (2023) [31]	U	U	L	H	H	L	L	L	L
C59	Timofeeva et al. (2023) [31]	U	U	L	H	H	L	L	L	L
C60	Timofeeva et al. (2022) [32]	U	U	U	H	H	L	L	L	L
C61	Timofeeva et al. (2022) [32]	U	U	U	H	H	L	L	L	L
C62	Timofeeva et al. (2022) [32]	U	U	U	H	H	L	L	L	L
C63	Timofeeva et al. (2022) [32]	U	U	U	H	H	L	L	L	L
C64	Timofeeva et al. (2022) [32]	U	U	U	H	H	L	L	L	L
C65	Timofeeva et al. (2022) [32]	U	U	U	H	H	L	L	L	L
C66	Timofeeva et al. (2022) [32]	U	U	U	H	H	L	L	L	L
C67	Timofeeva et al. (2022) [32]	U	U	U	H	H	L	L	L	L

Part C Supporting assessment details

Entry	Study [reference]	Target result or model	Recorded rationale
C01	Ali et al. (2022) [58]	Diagnostic. Comparator: OC vs benign ovarian tumours vs healthy controls; compared with CA125/CA19-9; performance: Sensitivity ≈80%; specificity ≈86.7%; combination improved accuracy	**Participants:** Non-nested case-control/two-gate sampling was used for model evaluation.<br>**Predictors:** Assay timing was appropriate, but blinding, transformations and identical measurement across participants were incompletely reported.<br>**Outcome:** Histopathological diagnosis is appropriate.<br>**Analysis:** Model development used limited data and/or data-driven predictor selection without demonstrated adequate validation and calibration.<br>**Applicability:** Population spectrum and intended clinical-use match are incompletely defined.

Entry	Study [reference]	Target result or model	Recorded rationale
C02	Choi et al. (2020) [49]	Diagnostic. Comparator: OC vs healthy controls; performance: AUC = 0.904 American model; 0.901 Chinese model	**Participants:** Non-nested case-control/two-gate sampling was used for model evaluation.<br>**Predictors:** Assay timing was appropriate, but blinding, transformations and identical measurement across participants were incompletely reported.<br>**Outcome:** Histopathological diagnosis is appropriate.<br>**Analysis:** Model development used limited data and/or data-driven predictor selection without demonstrated adequate validation and calibration.<br>**Applicability:** Population spectrum and intended clinical-use match are incompletely defined.
C03	Cirillo et al. (2021) [2]	Diagnostic. Comparator: OC vs healthy controls; early-/late-stage OC vs controls; performance: miRNA model AUC = 0.723; early-stage AUC = 0.789; miRNA + protein AUC = 0.989; early-stage combined AUC = 1.000, sensitivity 88.9%, specificity 100%	**Participants:** Non-nested case-control/two-gate sampling was used for model evaluation.<br>**Predictors:** Assay timing was appropriate, but blinding, transformations and identical measurement across participants were incompletely reported.<br>**Outcome:** Histopathological diagnosis is appropriate.<br>**Analysis:** Model development used limited data and/or data-driven predictor selection without demonstrated adequate validation and calibration.<br>**Applicability:** Population spectrum and intended clinical-use match are incompletely defined.
C04	Elias et al. (2017) [52]	Diagnostic. Comparator: OC vs benign disease, borderline tumours, healthy controls; performance: Sequencing model AUC ≈0.90; NN AUC ≈0.93 vs CA125 AUC ≈0.74; qPCR validation AUC ≈0.85; PPV 91.3%, NPV 78.6%, specificity up to 100%	**Participants:** Non-nested case-control/two-gate sampling was used for model evaluation.<br>**Predictors:** The signature was fixed before a substantial validation evaluation and predictors were available at intended use.<br>**Outcome:** Histopathological diagnosis is appropriate.<br>**Analysis:** Internal/external validation was reported, but calibration, missing-data handling, sample size and full model specification were not all verified.<br>**Applicability:** Population spectrum and intended clinical-use match are incompletely defined.
C05	Gockley et al. (2022) [51]	Diagnostic. Comparator: Benign adnexal masses + healthy controls; performance: Validation AUC = 0.87; sensitivity 73%; specificity 91% for early-stage detection	**Participants:** Non-nested case-control/two-gate sampling was used for model evaluation.<br>**Predictors:** The signature was fixed before a substantial validation evaluation and predictors were available at intended use.<br>**Outcome:** Histopathological diagnosis is appropriate.<br>**Analysis:** Internal/external validation was reported, but calibration, missing-data handling, sample size and full model specification were not all verified.<br>**Applicability:** Population spectrum and intended clinical-use match are incompletely defined.
C06	Hamidi et al. (2021) [55]	Diagnostic. Comparator: OC vs non-cancer controls; performance: AUC up to 100% using LR/RF/ANN/XGBoost; ≥95% in external validation	**Participants:** Representativeness, sampling frame and exclusions were not fully verifiable.<br>**Predictors:** The signature was fixed before a substantial validation evaluation and predictors were available at intended use.<br>**Outcome:** Histopathological diagnosis is appropriate.<br>**Analysis:** Internal/external validation was reported, but calibration, missing-data handling, sample size and full model specification were not all verified.<br>**Applicability:** Population spectrum and intended clinical-use match are incompletely defined.
C07	Hong et al. (2022) [54]	Diagnostic. Comparator: Non-cancer serum samples; other cancer types; performance: Sensitivity 97.5–98.33%; specificity 99.21–100%; PPV up to 100%, NPV up to 99.8%; AUC >0.92 for multi-cancer discrimination	**Participants:** Representativeness, sampling frame and exclusions were not fully verifiable.<br>**Predictors:** Assay timing was appropriate, but blinding, transformations and identical measurement across participants were incompletely reported.<br>**Outcome:** Histopathological diagnosis is appropriate.<br>**Analysis:** Model development used limited data and/or data-driven predictor selection without demonstrated adequate validation and calibration.<br>**Applicability:** The population is restricted, prediagnostic, subtype-specific, treatment-specific, or multi-cancer, limiting direct applicability.

Entry	Study [reference]	Target result or model	Recorded rationale
C08	Kan et al. (2012) [59]	Diagnostic. Comparator: OC vs healthy controls; performance: AUC = 0.784 for miR-200b + miR-200c model	**Participants:** Non-nested case-control/two-gate sampling was used for model evaluation.<br>**Predictors:** Assay timing was appropriate, but blinding, transformations and identical measurement across participants were incompletely reported.<br>**Outcome:** Histopathological diagnosis is appropriate.<br>**Analysis:** Model development used limited data and/or data-driven predictor selection without demonstrated adequate validation and calibration.<br>**Applicability:** Population spectrum and intended clinical-use match are incompletely defined.
C09	Kandimalla et al. (2021) [14]	Diagnostic. Comparator: OC vs healthy controls; specificity tested against other diseases/cancers; compared with CA125; performance: AUCs: tissue stage I 0.99; TCGA stage I 0.96; TCGA all stages 0.89; GSE65819 0.83; serum training 0.89; retrospective validation 0.82–0.86; prospective 0.92; stage I serum 0.81–0.90	**Participants:** Non-nested case-control/two-gate sampling was used for model evaluation.<br>**Predictors:** The signature was fixed before a substantial validation evaluation and predictors were available at intended use.<br>**Outcome:** Histopathological diagnosis is appropriate.<br>**Analysis:** Internal/external validation was reported, but calibration, missing-data handling, sample size and full model specification were not all verified.<br>**Applicability:** Population spectrum and intended clinical-use match are incompletely defined.
C10	Kumar et al. (2021) [13]	Diagnostic. Comparator: EOC vs healthy controls; performance: Individual AUCs: miR-205 87.6, miR-200c 78.2, miR-141 86.0; combined AUC 95.9 advanced, 95.2–98.1 early-stage; sensitivity up to 96.6%, specificity up to 100%	**Participants:** Non-nested case-control/two-gate sampling was used for model evaluation.<br>**Predictors:** Assay timing was appropriate, but blinding, transformations and identical measurement across participants were incompletely reported.<br>**Outcome:** Histopathological diagnosis is appropriate.<br>**Analysis:** Model development used limited data and/or data-driven predictor selection without demonstrated adequate validation and calibration.<br>**Applicability:** Population spectrum and intended clinical-use match are incompletely defined.
C11	Kumar et al. (2022) [36]	Diagnostic. Comparator: Healthy controls; normal ovarian tissue and serum; performance: Tissue AUCs: 92.1–97.0; serum AUCs: 85.6–92.0; combined panel AUC: tissue 98.6–98.9, serum 95.5–96.9	**Participants:** Non-nested case-control/two-gate sampling was used for model evaluation.<br>**Predictors:** Assay timing was appropriate, but blinding, transformations and identical measurement across participants were incompletely reported.<br>**Outcome:** Histopathological diagnosis is appropriate.<br>**Analysis:** Model development used limited data and/or data-driven predictor selection without demonstrated adequate validation and calibration.<br>**Applicability:** Population spectrum and intended clinical-use match are incompletely defined.
C12	Liang et al. (2020) [5]	Diagnostic. Comparator: OC vs benign disease vs healthy controls; performance: miR-183 AUC = 0.77; combined model AUC = 0.951, sensitivity 0.97, specificity 0.85	**Participants:** Non-nested case-control/two-gate sampling was used for model evaluation.<br>**Predictors:** Assay timing was appropriate, but blinding, transformations and identical measurement across participants were incompletely reported.<br>**Outcome:** Histopathological diagnosis is appropriate.<br>**Analysis:** Model development used limited data and/or data-driven predictor selection without demonstrated adequate validation and calibration.<br>**Applicability:** Population closely resembles the intended ovarian-cancer diagnostic/prognostic setting.

Entry	Study [reference]	Target result or model	Recorded rationale
C13	Niemira et al. (2023) [11]	Diagnostic. Comparator: HGSOC vs healthy controls; performance: Training AUC = 1.00, sensitivity 100%, specificity 92.3%; qPCR validation AUC = 0.946, sensitivity 100%, specificity 91.7%; external AUC ≈0.895	**Participants:** Non-nested case-control/two-gate sampling was used for model evaluation.<br>**Predictors:** The signature was fixed before a substantial validation evaluation and predictors were available at intended use.<br>**Outcome:** Histopathological diagnosis is appropriate.<br>**Analysis:** Internal/external validation was reported, but calibration, missing-data handling, sample size and full model specification were not all verified.<br>**Applicability:** The population is restricted, prediagnostic, subtype-specific, treatment-specific, or multi-cancer, limiting direct applicability.
C14	Oliveira et al. (2019) [10]	Diagnostic. Comparator: OC vs benign pelvic masses; performance: miR-200c-3p AUC = 0.78; miR-221-3p AUC ≈0.65; 2-miRNA panel AUC ≈0.82; with CA125 AUC ≈0.96, sensitivity ≈80%, specificity ≈95%	**Participants:** Representativeness, sampling frame and exclusions were not fully verifiable.<br>**Predictors:** Assay timing was appropriate, but blinding, transformations and identical measurement across participants were incompletely reported.<br>**Outcome:** Histopathological diagnosis is appropriate.<br>**Analysis:** Model development used limited data and/or data-driven predictor selection without demonstrated adequate validation and calibration.<br>**Applicability:** Population closely resembles the intended ovarian-cancer diagnostic/prognostic setting.
C15	Ren et al. (2018) [3]	Diagnostic. Comparator: EOC vs benign ovarian tumours and healthy controls; performance: miR-193a-5p AUC = 0.708, sensitivity 66.7%, specificity 72.5%; HE4 AUC = 0.877; CA125 AUC = 0.900; combined AUC = 0.996, sensitivity 93.3%, specificity 97.5%	**Participants:** Non-nested case-control/two-gate sampling was used for model evaluation.<br>**Predictors:** Assay timing was appropriate, but blinding, transformations and identical measurement across participants were incompletely reported.<br>**Outcome:** Histopathological diagnosis is appropriate.<br>**Analysis:** Model development used limited data and/or data-driven predictor selection without demonstrated adequate validation and calibration.<br>**Applicability:** Population closely resembles the intended ovarian-cancer diagnostic/prognostic setting.
C16	Stawiski et al. (2024) [65]	Diagnostic. Comparator: Cases close to diagnosis <2 years vs distant ≥2–7 years; no healthy controls; performance: Median AUC = 0.771; average AUC ≈0.781, 95% CI 0.72–0.84	**Participants:** Prospective/nested validation sampling was used.<br>**Predictors:** The signature was fixed before a substantial validation evaluation and predictors were available at intended use.<br>**Outcome:** Histopathological diagnosis is appropriate.<br>**Analysis:** Internal/external validation was reported, but calibration, missing-data handling, sample size and full model specification were not all verified.<br>**Applicability:** The population is restricted, prediagnostic, subtype-specific, treatment-specific, or multi-cancer, limiting direct applicability.
C17	Su et al. (2019) [6]	Diagnostic. Comparator: Benign ovarian tumours and healthy controls; compared with CA125/HE4; performance: miR-1307 AUC = 0.671–0.694; miR-375 AUC = 0.788; CA125 AUC = 0.905–0.906; HE4 AUC = 0.820; miR-1307+miR-375 AUC = 0.837; +CA125 AUC ≈0.970–0.977	**Participants:** Non-nested case-control/two-gate sampling was used for model evaluation.<br>**Predictors:** Assay timing was appropriate, but blinding, transformations and identical measurement across participants were incompletely reported.<br>**Outcome:** Histopathological diagnosis is appropriate.<br>**Analysis:** Model development used limited data and/or data-driven predictor selection without demonstrated adequate validation and calibration.<br>**Applicability:** Population closely resembles the intended ovarian-cancer diagnostic/prognostic setting.

Entry	Study [reference]	Target result or model	Recorded rationale
C18	Wang et al. (2019a) [37]	Diagnostic five-serum-miRNA model (miR-200c-3p, miR-346, miR-127-3p, miR-143-3p, miR-205-5p): training AUC 0.783; validation 0.745.	**Participants:** Non-nested case-control/two-gate sampling was used for model evaluation.<br>**Predictors:** Assay timing was appropriate, but blinding, transformations and identical measurement across participants were incompletely reported.<br>**Outcome:** Histopathological diagnosis is appropriate.<br>**Analysis:** Model development used limited data and/or data-driven predictor selection without demonstrated adequate validation and calibration.<br>**Applicability:** Population spectrum and intended clinical-use match are incompletely defined.
C19	Wang et al. (2022) [53]	Diagnostic. Comparator: Benign peritoneal fluid + benign plasma controls; performance: Ascites AUC = 1.00; plasma AUC ≈0.98 training, 0.94 validation; external datasets AUC ≈0.90–0.96	**Participants:** Representativeness, sampling frame and exclusions were not fully verifiable.<br>**Predictors:** The signature was fixed before a substantial validation evaluation and predictors were available at intended use.<br>**Outcome:** Histopathological diagnosis is appropriate.<br>**Analysis:** Internal/external validation was reported, but calibration, missing-data handling, sample size and full model specification were not all verified.<br>**Applicability:** Population spectrum and intended clinical-use match are incompletely defined.
C20	Webber et al. (2024) [4]	Diagnostic. Comparator: Benign adnexal masses + healthy women; miRNA vs protein/metadata vs combined model; performance: External validation AUC = 0.95, 95% CI 0.90–0.98; sensitivity 92%; specificity 80%; early-stage sensitivity 80%; late-stage 100%	**Participants:** Prospective/nested validation sampling was used.<br>**Predictors:** The signature was fixed before a substantial validation evaluation and predictors were available at intended use.<br>**Outcome:** Histopathological diagnosis is appropriate.<br>**Analysis:** Internal/external validation was reported, but calibration, missing-data handling, sample size and full model specification were not all verified.<br>**Applicability:** Population closely resembles the intended ovarian-cancer diagnostic/prognostic setting.
C21	Wiczling et al. (2019) [57]	Diagnostic. Comparator: OC vs healthy controls; performance: Individual AUC ≈0.65; combined subset AUC ≈0.72; overall limited performance	**Participants:** Non-nested case-control/two-gate sampling was used for model evaluation.<br>**Predictors:** Assay timing was appropriate, but blinding, transformations and identical measurement across participants were incompletely reported.<br>**Outcome:** Histopathological diagnosis is appropriate.<br>**Analysis:** Model development used limited data and/or data-driven predictor selection without demonstrated adequate validation and calibration.<br>**Applicability:** Population spectrum and intended clinical-use match are incompletely defined.
C22	Yang et al. (2024) [56]	Diagnostic. Comparator: EOC vs benign tumours vs healthy controls; performance: miR-223 AUC = 0.812; CA125 AUC = 0.933; miR-223+CA125 AUC = 0.937, sensitivity ≈94.9%, specificity ≈81.5%; early-stage AUC = 0.94, sensitivity ≈97.8%, specificity ≈88.5%	**Participants:** Non-nested case-control/two-gate sampling was used for model evaluation.<br>**Predictors:** Assay timing was appropriate, but blinding, transformations and identical measurement across participants were incompletely reported.<br>**Outcome:** Histopathological diagnosis is appropriate.<br>**Analysis:** Model development used limited data and/or data-driven predictor selection without demonstrated adequate validation and calibration.<br>**Applicability:** Population closely resembles the intended ovarian-cancer diagnostic/prognostic setting.

Entry	Study [reference]	Target result or model	Recorded rationale
C23	Yokoi et al. (2017) [50]	Diagnostic. Comparator: OC vs healthy; early OC vs benign; performance: AUC = 0.97 for OC vs healthy; AUC = 0.90 for early OC vs benign	**Participants:** Representativeness, sampling frame and exclusions were not fully verifiable.<br>**Predictors:** Assay timing was appropriate, but blinding, transformations and identical measurement across participants were incompletely reported.<br>**Outcome:** Histopathological diagnosis is appropriate.<br>**Analysis:** Model development used limited data and/or data-driven predictor selection without demonstrated adequate validation and calibration.<br>**Applicability:** Population closely resembles the intended ovarian-cancer diagnostic/prognostic setting.
C24	Yokoi et al. (2018) [8]	Diagnostic. Comparator: OC vs benign tumours vs healthy controls; performance: AUC ≈0.99; sensitivity/specificity >95%; strong stage I–II performance	**Participants:** Non-nested case-control/two-gate sampling was used for model evaluation.<br>**Predictors:** The signature was fixed before a substantial validation evaluation and predictors were available at intended use.<br>**Outcome:** Histopathological diagnosis is appropriate.<br>**Analysis:** Internal/external validation was reported, but calibration, missing-data handling, sample size and full model specification were not all verified.<br>**Applicability:** Population closely resembles the intended ovarian-cancer diagnostic/prognostic setting.
C25	Zhang & Hu (2022) [68]	Diagnostic. Comparator: Non-cancer controls; performance: Lung validation AUC = 0.999, sensitivity/specificity 99%; multi-cancer sensitivity 68.2% for ovarian and up to 100% for others; specificity 99.3%	**Participants:** Non-nested case-control/two-gate sampling was used for model evaluation.<br>**Predictors:** Assay timing was appropriate, but blinding, transformations and identical measurement across participants were incompletely reported.<br>**Outcome:** Histopathological diagnosis is appropriate.<br>**Analysis:** Model development used limited data and/or data-driven predictor selection without demonstrated adequate validation and calibration.<br>**Applicability:** The population is restricted, prediagnostic, subtype-specific, treatment-specific, or multi-cancer, limiting direct applicability.
C26	Zhu et al. (2022) [47]	Diagnostic. Comparator: OC vs benign vs healthy controls; performance: miR-205 AUC = 0.715, sensitivity 66.7%, specificity 78.1%; combined miR-205+CA125+HE4 AUC up to 0.951	**Participants:** Non-nested case-control/two-gate sampling was used for model evaluation.<br>**Predictors:** Assay timing was appropriate, but blinding, transformations and identical measurement across participants were incompletely reported.<br>**Outcome:** Histopathological diagnosis is appropriate.<br>**Analysis:** Model development used limited data and/or data-driven predictor selection without demonstrated adequate validation and calibration.<br>**Applicability:** Population closely resembles the intended ovarian-cancer diagnostic/prognostic setting.
C29	Uehara et al. (2024) [78]	Prognostic. Outcomes: PFS, treatment selection (PDS vs NACT), tumour respectability; analysis: Cox regression (LOO-LASSO), Kaplan–Meier, log-rank, ROC, propensity score matching	**Participants:** Representativeness, sampling frame and exclusions were not fully verifiable.<br>**Predictors:** Assay timing was appropriate, but blinding, transformations and identical measurement across participants were incompletely reported.<br>**Outcome:** Outcome definition/timing for response, treatment selection or surgical result was incompletely specified.<br>**Analysis:** Model development used limited data and/or data-driven predictor selection without demonstrated adequate validation and calibration.<br>**Applicability:** The population is restricted, prediagnostic, subtype-specific, treatment-specific, or multi-cancer, limiting direct applicability.

Entry	Study [reference]	Target result or model	Recorded rationale
C30	Yoshida et al. (2021) [12]	Prognostic. Outcomes: OS, PFS; analysis: Kaplan–Meier, log-rank, univariate/multivariate Cox regression, prognostic index modelling	**Participants:** Representativeness, sampling frame and exclusions were not fully verifiable.<br>**Predictors:** The signature was fixed before a substantial validation evaluation and predictors were available at intended use.<br>**Outcome:** OS/PFS is an appropriate outcome.<br>**Analysis:** Internal/external validation was reported, but calibration, missing-data handling, sample size and full model specification were not all verified.<br>**Applicability:** Population spectrum and intended clinical-use match are incompletely defined.
C31	Zhong et al. (2025) [71]	Prognostic. Outcomes: Chemotherapy response, survival, prognostic risk factors; analysis: Chi-square, t-test, logistic regression, ROC analysis	**Participants:** Representativeness, sampling frame and exclusions were not fully verifiable.<br>**Predictors:** Assay timing was appropriate, but blinding, transformations and identical measurement across participants were incompletely reported.<br>**Outcome:** Outcome definition/timing for response, treatment selection or surgical result was incompletely specified.<br>**Analysis:** Model development used limited data and/or data-driven predictor selection without demonstrated adequate validation and calibration.<br>**Applicability:** Population spectrum and intended clinical-use match are incompletely defined.
C32	Chen et al. (2020) [85]	Diagnostic. Diagnostic: miR-125b: AUC≈0.73, Se1 76%, Sp2 41.6%; combined with CA125: AUC≈0.90, Se 83.6%, Sp 95.6%; prognostic: Low miR-125b linked to advanced stage, LN metastasis, chemoresistance, poorer prognosis; higher post-surgery	**Participants:** Representativeness, sampling frame and exclusions were not fully verifiable.<br>**Predictors:** Assay timing was appropriate, but blinding, transformations and identical measurement across participants were incompletely reported.<br>**Outcome:** Histopathological diagnosis is appropriate.<br>**Analysis:** Model development used limited data and/or data-driven predictor selection without demonstrated adequate validation and calibration.<br>**Applicability:** Population spectrum and intended clinical-use match are incompletely defined.
C33	Gahlawat et al. (2022) [9]	Diagnostic. Diagnostic: Panel: AUC=0.87; benign vs control AUC=0.77; early-stage AUC=0.81; combined with CA125: AUC=0.97 overall, 0.93 early-stage; prognostic: High cf-miRNA associated with worse OS; independent prognostic marker (HR≈1.83); no PFS association	**Participants:** Representativeness, sampling frame and exclusions were not fully verifiable.<br>**Predictors:** Assay timing was appropriate, but blinding, transformations and identical measurement across participants were incompletely reported.<br>**Outcome:** Histopathological diagnosis is appropriate.<br>**Analysis:** Model development used limited data and/or data-driven predictor selection without demonstrated adequate validation and calibration.<br>**Applicability:** Population spectrum and intended clinical-use match are incompletely defined.
C34	Helmy et al. (2025) [80]	Diagnostic. Diagnostic: miR-200b: AUC=0.743, Se 63.3%, Sp 71%; miR-200b+CA125: Se 100%, Sp 93.1%; prognostic: High miR-200b linked to shorter EFS	**Participants:** Representativeness, sampling frame and exclusions were not fully verifiable.<br>**Predictors:** Assay timing was appropriate, but blinding, transformations and identical measurement across participants were incompletely reported.<br>**Outcome:** Histopathological diagnosis is appropriate.<br>**Analysis:** Model development used limited data and/or data-driven predictor selection without demonstrated adequate validation and calibration.<br>**Applicability:** Population spectrum and intended clinical-use match are incompletely defined.
C35	Li et al. (2023) [83]	Diagnostic. Diagnostic: Training AUC=0.913; testing AUC=0.973; validation AUC=0.924; early-stage AUC=0.903; prognostic: High miR-141-3p and miR-200c-3p linked to poor OS; RiskScore predicts PFS	**Participants:** Representativeness, sampling frame and exclusions were not fully verifiable.<br>**Predictors:** The signature was fixed before a substantial validation evaluation and predictors were available at intended use.<br>**Outcome:** Histopathological diagnosis is appropriate.<br>**Analysis:** Internal/external validation was reported, but calibration, missing-data handling, sample size and full model specification were not all verified.<br>**Applicability:** Population closely resembles the intended ovarian-cancer diagnostic/prognostic setting.

Entry	Study [reference]	Target result or model	Recorded rationale
C36	Li et al. (2023) [83]	Prognostic. Diagnostic: Training AUC=0.913; testing AUC=0.973; validation AUC=0.924; early-stage AUC=0.903; prognostic: High miR-141-3p and miR-200c-3p linked to poor OS; RiskScore predicts PFS	**Participants:** Representativeness, sampling frame and exclusions were not fully verifiable. **Predictors:** The signature was fixed before a substantial validation evaluation and predictors were available at intended use. **Outcome:** OS/PFS is an appropriate outcome. **Analysis:** Internal/external validation was reported, but calibration, missing-data handling, sample size and full model specification were not all verified. **Applicability:** Population closely resembles the intended ovarian-cancer diagnostic/prognostic setting.
C37	Meng et al. (2015) [40]	Diagnostic. Diagnostic: Se 93%; Sp 92%; prognostic: miR-429 predicts OS; miR-7 linked to metastasis; miR-376a linked to advanced stage	**Participants:** Representativeness, sampling frame and exclusions were not fully verifiable. **Predictors:** Assay timing was appropriate, but blinding, transformations and identical measurement across participants were incompletely reported. **Outcome:** Histopathological diagnosis is appropriate. **Analysis:** Model development used limited data and/or data-driven predictor selection without demonstrated adequate validation and calibration. **Applicability:** Population spectrum and intended clinical-use match are incompletely defined.
C38	Meng et al. (2016) [39]	Diagnostic. Diagnostic: miR-200a AUC=0.914; miR-200b AUC=0.815; combined panel AUC=0.925, Se 88%, Sp 90%; prognostic: miR-200b/c associated with poor OS and DFS; independent prognostic factors	**Participants:** Representativeness, sampling frame and exclusions were not fully verifiable. **Predictors:** Assay timing was appropriate, but blinding, transformations and identical measurement across participants were incompletely reported. **Outcome:** Histopathological diagnosis is appropriate. **Analysis:** Model development used limited data and/or data-driven predictor selection without demonstrated adequate validation and calibration. **Applicability:** Population spectrum and intended clinical-use match are incompletely defined.
C39	Minareci et al. (2024) [92]	Diagnostic. Diagnostic: miR-4314 AUC=0.78; miR-1181 AUC=0.76; combined AUC=0.81; prognostic: High expression linked to worse OS and DFS	**Participants:** Representativeness, sampling frame and exclusions were not fully verifiable. **Predictors:** Assay timing was appropriate, but blinding, transformations and identical measurement across participants were incompletely reported. **Outcome:** Histopathological diagnosis is appropriate. **Analysis:** Model development used limited data and/or data-driven predictor selection without demonstrated adequate validation and calibration. **Applicability:** Population spectrum and intended clinical-use match are incompletely defined.
C40	Ranmale et al. (2025) [93]	Diagnostic. Diagnostic: Combined panel: AUC=0.743, Se 81.6%, Sp 60.5%; prognostic: serum miR-130a associated with PFS; tissue miR-20a excluded from circulating evidence; several miRNAs linked to relapse and chemo response	**Participants:** Representativeness, sampling frame and exclusions were not fully verifiable. **Predictors:** Assay timing was appropriate, but blinding, transformations and identical measurement across participants were incompletely reported. **Outcome:** Histopathological diagnosis is appropriate. **Analysis:** Model development used limited data and/or data-driven predictor selection without demonstrated adequate validation and calibration. **Applicability:** Population closely resembles the intended ovarian-cancer diagnostic/prognostic setting.

Entry	Study [reference]	Target result or model	Recorded rationale
C41	Shah et al. (2018) [82]	Diagnostic logistic models combining CA-125 with miR-375 (AUC 0.956), or miR-375 and miR-210 (AUC 0.984), for HGSOC versus healthy controls. This entry does not assess the separate miR-34a-5p surgical-outcome model.	**Participants:** Representativeness, sampling frame and exclusions were not fully verifiable.<br>**Predictors:** Assay timing was appropriate, but blinding, transformations and identical measurement across participants were incompletely reported.<br>**Outcome:** Outcome definition/timing for response, treatment selection or surgical result was incompletely specified.<br>**Analysis:** Model development used limited data and/or data-driven predictor selection without demonstrated adequate validation and calibration.<br>**Applicability:** Population spectrum and intended clinical-use match are incompletely defined.
C42	Song et al. (2020) [87]	Diagnostic. Diagnostic: miR-26b AUC=0.753; miR-21 AUC=0.826; combined AUC=0.905 in Table III (0.916 in source narrative; unresolved discrepancy); prognostic: miR-26b linked to better survival; miR-21 linked to worse survival	**Participants:** Representativeness, sampling frame and exclusions were not fully verifiable.<br>**Predictors:** Assay timing was appropriate, but blinding, transformations and identical measurement across participants were incompletely reported.<br>**Outcome:** Histopathological diagnosis is appropriate.<br>**Analysis:** Model development used limited data and/or data-driven predictor selection without demonstrated adequate validation and calibration.<br>**Applicability:** Population spectrum and intended clinical-use match are incompletely defined.
C43	Wang et al. (2019b) [38]	Diagnostic five-plasma-miRNA model (miR-205-5p, miR-145-5p, miR-10a-5p, miR-346, miR-328-3p): training AUC 0.788; validation 0.763. Tumour-grade association is not survival prediction.	**Participants:** Representativeness, sampling frame and exclusions were not fully verifiable.<br>**Predictors:** Assay timing was appropriate, but blinding, transformations and identical measurement across participants were incompletely reported.<br>**Outcome:** Histopathological diagnosis is appropriate.<br>**Analysis:** Model development used limited data and/or data-driven predictor selection without demonstrated adequate validation and calibration.<br>**Applicability:** Population spectrum and intended clinical-use match are incompletely defined.
C44	Zhang et al. (2020) [81]	Diagnostic. Diagnostic: miR-484: AUC=0.821, Se 77.9%, Sp 80%; combined with CA125: AUC=0.912; prognostic: Low miR-484 linked to poor OS/PFS and aggressive disease	**Participants:** Representativeness, sampling frame and exclusions were not fully verifiable.<br>**Predictors:** Assay timing was appropriate, but blinding, transformations and identical measurement across participants were incompletely reported.<br>**Outcome:** Histopathological diagnosis is appropriate.<br>**Analysis:** Model development used limited data and/or data-driven predictor selection without demonstrated adequate validation and calibration.<br>**Applicability:** Population spectrum and intended clinical-use match are incompletely defined.
C45	Zheng et al. (2013) [95]	Diagnostic. Diagnostic: High diagnostic accuracy; improved with CA125; prognostic: Low let-7f linked to poor prognosis	**Participants:** Representativeness, sampling frame and exclusions were not fully verifiable.<br>**Predictors:** Assay timing was appropriate, but blinding, transformations and identical measurement across participants were incompletely reported.<br>**Outcome:** Histopathological diagnosis is appropriate.<br>**Analysis:** Model development used limited data and/or data-driven predictor selection without demonstrated adequate validation and calibration.<br>**Applicability:** Population closely resembles the intended ovarian-cancer diagnostic/prognostic setting.

Entry	Study [reference]	Target result or model	Recorded rationale
C46	Zhou et al. (2023) [20]	Four-sEV-miRNA surgical residual-disease model	**Participants:** balanced small discovery sample (15 R0/15 non-R0) and selected advanced HGSOC. Predictors: plasma measured preoperatively, with specified log transformations; blinding was not clearly reported. Outcome: surgical residual disease; assessment blinding and consistency across surgical pathways uncertain. Analysis: LASSO selection and model development in n=30; leave-one-out cross-validation not explicitly nested for selection; formal calibration statistics/plots were not identified, despite the abstract's claim of favourable calibration. Separate validation n=154 does not remove development limitations. Applicable to advanced-HGSOC surgical prediction, not population screening.
C47	Zhou et al. (2023) [20]	Four-sEV-miRNA plus CA-125 surgical residual-disease model	**Participants:** balanced small discovery sample (15 R0/15 non-R0) and selected advanced HGSOC. Predictors: plasma measured preoperatively, with specified log transformations; blinding was not clearly reported. Outcome: surgical residual disease; assessment blinding and consistency across surgical pathways uncertain. Analysis: LASSO selection and model development in n=30; leave-one-out cross-validation not explicitly nested for selection; formal calibration statistics/plots were not identified, despite the abstract's claim of favourable calibration. Separate validation n=154 does not remove development limitations. Applicable to advanced-HGSOC surgical prediction, not population screening.
C48	Suryawanshi et al. (2013) [22]	EAOC versus healthy controls: miR-16/21/191	Participants high: selected histotype-specific cases/comparators; commercial healthy controls where applicable. Predictors unclear: assay blinding insufficiently described. Outcome low: histologically confirmed cancer/endometriosis and defined healthy controls. Analysis high: small samples, extensive candidate selection, discovery samples reused, LOOCV not explicitly nested for feature selection, missing measurements, and no calibration or external validation. Applicability high for broad OC detection because the model targets selected histotypes/comparators.
C49	Suryawanshi et al. (2013) [22]	EAOC versus endometriosis: miR-21/362-5p/1274a	Participants high: selected histotype-specific cases/comparators; commercial healthy controls where applicable. Predictors unclear: assay blinding insufficiently described. Outcome low: histologically confirmed cancer/endometriosis and defined healthy controls. Analysis high: small samples, extensive candidate selection, discovery samples reused, LOOCV not explicitly nested for feature selection, missing measurements, and no calibration or external validation. Applicability high for broad OC detection because the model targets selected histotypes/comparators.
C50	Suryawanshi et al. (2013) [22]	Serous OC versus healthy controls: miR-16/191/4284	Participants high: selected histotype-specific cases/comparators; commercial healthy controls where applicable. Predictors unclear: assay blinding insufficiently described. Outcome low: histologically confirmed cancer/endometriosis and defined healthy controls. Analysis high: small samples, extensive candidate selection, discovery samples reused, LOOCV not explicitly nested for feature selection, missing measurements, and no calibration or external validation. Applicability high for broad OC detection because the model targets selected histotypes/comparators.
C51	Suryawanshi et al. (2013) [22]	Serous OC versus endometriosis: miR-362-5p/628-3p/1915	Participants high: selected histotype-specific cases/comparators; commercial healthy controls where applicable. Predictors unclear: assay blinding insufficiently described. Outcome low: histologically confirmed cancer/endometriosis and defined healthy controls. Analysis high: small samples, extensive candidate selection, discovery samples reused, LOOCV not explicitly nested for feature selection, missing measurements, and no calibration or external validation. Applicability high for broad OC detection because the model targets selected histotypes/comparators.
C52	Meng et al. (2016) [25]	Three-miR-200 diagnostic panel (Figure 1.1)	Participants high: selected case-control diagnostic sample. Predictors unclear: assay blinding and handling of missing measurements incompletely specified. Outcome unclear: diagnostic comparator labels or clinical endpoint ascertainment incompletely consistent/described. Analysis high: small development cohort with data-driven model selection; apparent performance without independent validation, no established calibration or adequate optimism correction. Applicability high for the broad diagnostic population owing to selected comparators.

Entry	Study [reference]	Target result or model	Recorded rationale
C53	Tian et al. (2025) [29]	CMD miRNA–circRNA diagnostic model (Figure 8; overall and subgroup evaluations of the same model)	Participants high: selected case-control diagnostic sample. Predictors unclear: assay blinding and handling of missing measurements incompletely specified. Outcome low: histological diagnosis or specified RECIST response. Analysis high: small development cohort with data-driven model selection; apparent performance without independent validation, no established calibration or adequate optimism correction. Applicability high for the broad diagnostic population owing to selected comparators.
C54	Timofeeva et al. (2023) [31]	Chemotherapy-response model 2, source Table 8; circulating miRNA–piRNA combination	Participants unclear: representativeness and complete participant flow insufficiently reported. Predictors unclear: assay blinding and handling of missing measurements incompletely specified. Outcome low: histological diagnosis or specified RECIST response. Analysis high: small development cohort with data-driven model selection; apparent performance without independent validation, no established calibration or adequate optimism correction. Applicability low for the specified serous-cancer treatment/surgical context; not a population-screening model.
C55	Timofeeva et al. (2023) [31]	Chemotherapy-response model 3, source Table 8; circulating miRNA–piRNA combination	Participants unclear: representativeness and complete participant flow insufficiently reported. Predictors unclear: assay blinding and handling of missing measurements incompletely specified. Outcome low: histological diagnosis or specified RECIST response. Analysis high: small development cohort with data-driven model selection; apparent performance without independent validation, no established calibration or adequate optimism correction. Applicability low for the specified serous-cancer treatment/surgical context; not a population-screening model.
C56	Timofeeva et al. (2023) [31]	Chemotherapy-response model 5, source Table 8; circulating miRNA–piRNA combination	Participants unclear: representativeness and complete participant flow insufficiently reported. Predictors unclear: assay blinding and handling of missing measurements incompletely specified. Outcome low: histological diagnosis or specified RECIST response. Analysis high: small development cohort with data-driven model selection; apparent performance without independent validation, no established calibration or adequate optimism correction. Applicability low for the specified serous-cancer treatment/surgical context; not a population-screening model.
C57	Timofeeva et al. (2023) [31]	Chemotherapy-response model 6, source Table 8; circulating miRNA–piRNA combination	Participants unclear: representativeness and complete participant flow insufficiently reported. Predictors unclear: assay blinding and handling of missing measurements incompletely specified. Outcome low: histological diagnosis or specified RECIST response. Analysis high: small development cohort with data-driven model selection; apparent performance without independent validation, no established calibration or adequate optimism correction. Applicability low for the specified serous-cancer treatment/surgical context; not a population-screening model.
C58	Timofeeva et al. (2023) [31]	Chemotherapy-response model 7, source Table 8; circulating miRNA–piRNA combination	Participants unclear: representativeness and complete participant flow insufficiently reported. Predictors unclear: assay blinding and handling of missing measurements incompletely specified. Outcome low: histological diagnosis or specified RECIST response. Analysis high: small development cohort with data-driven model selection; apparent performance without independent validation, no established calibration or adequate optimism correction. Applicability low for the specified serous-cancer treatment/surgical context; not a population-screening model.
C59	Timofeeva et al. (2023) [31]	Chemotherapy-response model 8, source Table 8; circulating miRNA–piRNA combination	Participants unclear: representativeness and complete participant flow insufficiently reported. Predictors unclear: assay blinding and handling of missing measurements incompletely specified. Outcome low: histological diagnosis or specified RECIST response. Analysis high: small development cohort with data-driven model selection; apparent performance without independent validation, no established calibration or adequate optimism correction. Applicability low for the specified serous-cancer treatment/surgical context; not a population-screening model.

Entry	Study [reference]	Target result or model	Recorded rationale
C60	Timofeeva et al. (2022) [32]	Surgical outcome model 1, source Figure 7a	Participants unclear: representativeness and complete participant flow insufficiently reported. Predictors unclear: assay blinding and handling of missing measurements incompletely specified. Outcome unclear: diagnostic comparator labels or clinical endpoint ascertainment incompletely consistent/described. Analysis high: small development cohort with data-driven model selection; apparent performance without independent validation, no established calibration or adequate optimism correction. Applicability low for the specified serous-cancer treatment/surgical context; not a population-screening model.
C61	Timofeeva et al. (2022) [32]	Surgical outcome model 2, source Figure 7a	Participants unclear: representativeness and complete participant flow insufficiently reported. Predictors unclear: assay blinding and handling of missing measurements incompletely specified. Outcome unclear: diagnostic comparator labels or clinical endpoint ascertainment incompletely consistent/described. Analysis high: small development cohort with data-driven model selection; apparent performance without independent validation, no established calibration or adequate optimism correction. Applicability low for the specified serous-cancer treatment/surgical context; not a population-screening model.
C62	Timofeeva et al. (2022) [32]	Surgical outcome model 3, source Figure 7a	Participants unclear: representativeness and complete participant flow insufficiently reported. Predictors unclear: assay blinding and handling of missing measurements incompletely specified. Outcome unclear: diagnostic comparator labels or clinical endpoint ascertainment incompletely consistent/described. Analysis high: small development cohort with data-driven model selection; apparent performance without independent validation, no established calibration or adequate optimism correction. Applicability low for the specified serous-cancer treatment/surgical context; not a population-screening model.
C63	Timofeeva et al. (2022) [32]	Surgical outcome model 4, source Figure 7a	Participants unclear: representativeness and complete participant flow insufficiently reported. Predictors unclear: assay blinding and handling of missing measurements incompletely specified. Outcome unclear: diagnostic comparator labels or clinical endpoint ascertainment incompletely consistent/described. Analysis high: small development cohort with data-driven model selection; apparent performance without independent validation, no established calibration or adequate optimism correction. Applicability low for the specified serous-cancer treatment/surgical context; not a population-screening model.
C64	Timofeeva et al. (2022) [32]	Chemotherapy response model 1, source Figure 7b	Participants unclear: representativeness and complete participant flow insufficiently reported. Predictors unclear: assay blinding and handling of missing measurements incompletely specified. Outcome unclear: diagnostic comparator labels or clinical endpoint ascertainment incompletely consistent/described. Analysis high: small development cohort with data-driven model selection; apparent performance without independent validation, no established calibration or adequate optimism correction. Applicability low for the specified serous-cancer treatment/surgical context; not a population-screening model.
C65	Timofeeva et al. (2022) [32]	Chemotherapy response model 2, source Figure 7b	Participants unclear: representativeness and complete participant flow insufficiently reported. Predictors unclear: assay blinding and handling of missing measurements incompletely specified. Outcome unclear: diagnostic comparator labels or clinical endpoint ascertainment incompletely consistent/described. Analysis high: small development cohort with data-driven model selection; apparent performance without independent validation, no established calibration or adequate optimism correction. Applicability low for the specified serous-cancer treatment/surgical context; not a population-screening model.
C66	Timofeeva et al. (2022) [32]	Chemotherapy response model 3, source Figure 7b	Participants unclear: representativeness and complete participant flow insufficiently reported. Predictors unclear: assay blinding and handling of missing measurements incompletely specified. Outcome unclear: diagnostic comparator labels or clinical endpoint ascertainment incompletely consistent/described. Analysis high: small development cohort with data-driven model selection; apparent performance without independent validation, no established calibration or adequate optimism correction. Applicability low for the specified serous-cancer treatment/surgical context; not a population-screening model.

Entry	Study [reference]	Target result or model	Recorded rationale
C67	Timofeeva et al. (2022) [32]	Chemotherapy response model 4, source Figure 7b	Participants unclear: representativeness and complete participant flow insufficiently reported. Predictors unclear: assay blinding and handling of missing measurements incompletely specified. Outcome unclear: diagnostic comparator labels or clinical endpoint ascertainment incompletely consistent/described. Analysis high: small development cohort with data-driven model selection; apparent performance without independent validation, no established calibration or adequate optimism correction. Applicability low for the specified serous-cancer treatment/surgical context; not a population-screening model.

Tool references

Whiting PF et al. QUADAS-2: a revised tool for the quality assessment of diagnostic accuracy studies. Ann Intern Med. 2011;155:529–536. doi:10.7326/0003-4819-155-8-201110180-00009.

Hayden JA et al. Assessing bias in studies of prognostic factors. Ann Intern Med. 2013;158:280–286. doi:10.7326/0003-4819-158-4-201302190-00009.

Wolff RF et al. PROBAST: a tool to assess the risk of bias and applicability of prediction model studies. Ann Intern Med. 2019;170:51–58. doi:10.7326/M18-1376.
