# Supplementary material for: Circulating miRNAs and Multivariate Biomarker Signatures in Ovarian Cancer: A Systematic Review
Source: Int J Mol Sci. 2026 Oct 2;27(19):8823. doi: 10.3390/ijms27198823 (PMC13664815; doi:10.3390/ijms27198823)
Supplement: Supplementary file 1 [file ijms-27-08823-s001.zip › Supplementary_Table_S5_Source_Verified_AUC_Values.pdf]

Supplementary Table S5. Reported diagnostic AUC values and study-level calculations underlying Figure 8

Figure 8 summarizes the reported values listed below. Source locations and discrepancies are stated where checked; public-data model refits are distinguished from validation of an unchanged model. Eligible estimates were blood-based diagnostic ROC-AUCs comparing ovarian cancer with non-cancer controls (healthy and/or benign comparators, as reported by each study). Each publication contributed at most one study-level representative AUC per Figure 8 category. Post-training test estimates, including explicitly labelled model refits, were preferred over training/discovery estimates when corresponding validation estimates were available. When more than one eligible post-training or otherwise eligible diagnostic AUC was reported within the same publication and category, the median was used as the representative value. No weighting by sample size, study quality, confidence-interval width, or precision was applied. Tissue, urine and ascites results; non-ovarian validation; prognostic, recurrence, treatment-response and surgical-outcome AUCs; and biomarker-only models without miRNA were excluded. The individual-miRNA category includes conventional standalone single-marker ROC analyses, but excludes feature-level AUCs generated solely within machine-learning feature-selection/panel-development workflows. The miRNA + CA125/HE4 category contains models using miRNA plus CA125 and/or HE4 only; models adding other protein biomarkers or clinical variables were classified as multimodal.

Figure 8 calculation: within each category, the minimum and maximum of the study-level representative AUCs define the plotted range, and the plotted point is the arithmetic midpoint: (minimum + maximum)/2. Final midpoints are 0.7365, 0.8425, 0.9480 and 0.9745 for the four categories, respectively.

Figure 8 summary

Category	No. contributing publications	Minimum AUC	Maximum AUC	Arithmetic midpoint
Individual circulating miRNA	39	0.540	0.933	0.7365
Multi-miRNA panel/signature	30	0.685	1.000	0.8425
miRNA + CA125 and/or HE4	12	0.900	0.996	0.9480
Multimodal model	3	0.950	0.999	0.9745

A. Individual circulating miRNA

Study	Biomarker/model	Figure 8 category	Eligible raw AUC value(s)	Representative AUC	Selection note
Gumusoglu-Acar et al. (2023)	miR-1909-5p; miR-885-5p; let-7d-3p	Individual circulating miRNA	1.000; 0.780; 0.700	0.78	OC versus healthy controls; same-cohort blood-miRNA ROC analyses, source Figure 3A. The source alternates serum/plasma terminology despite EDTA collection; coded as plasma in Table 4.
Khan et al. (2023)	miR-223	Individual circulating miRNA	0.7915	0.7915	Whole-blood single-marker ROC.
Kumar et al. (2021)	miR-205; miR-200c; miR-141	Individual circulating miRNA	0.876; 0.782; 0.860; 0.881; 0.789; 0.867	0.8635	Advanced- and early-stage serum ROC estimates.
Kumar et al. (2022)	miR-34a; let-7f; miR-31	Individual circulating miRNA	0.920; 0.879; 0.856; 0.827; 0.820; 0.810	0.8415	Advanced- and early-stage serum ROC estimates.
Liang et al. (2020)	miR-183	Individual circulating miRNA	0.770	0.77	Standalone circulating-miRNA ROC.
Márton et al. (2019)	Eight circulating miRNAs	Individual circulating miRNA	0.845; 0.822; 0.861; 0.825; 0.791; 0.831; 0.655; 0.832	0.828	All eligible standalone single-miRNA AUCs reported.
Oliveira et al. (2019)	Six individual plasma miRNAs	Individual circulating miRNA	0.780; 0.650; 0.630; 0.630; 0.630; 0.620	0.63	Discovery single-marker ROC analyses.
Ren et al. (2018)	miR-193a-5p	Individual circulating miRNA	0.708	0.708	Standalone serum miRNA ROC.
Su et al. (2019)	Exosomal miR-1307; miR-375	Individual circulating miRNA	0.671; 0.788	0.7295	Source Table 3: miR-1307 0.671 and miR-375 0.788. The text reports a conflicting miR-1307 value of 0.694; the tabulated value is used without assuming a typographical error.
Takamizawa et al. (2024)	miR-146a-5p; miR-191-5p	Individual circulating miRNA	0.762; 0.830	0.796	Ovarian clear-cell carcinoma vs healthy controls.
Todeschini et al. (2017)	miR-1246; miR-595; miR-2278	Individual circulating miRNA	0.890; 0.690; 0.760	0.76	All eligible standalone serum-miRNA ROC estimates.
Wiczling et al. (2019)	49 individual circulating miRNAs (source Table A order)	Individual circulating miRNA	0.490; 0.590; 0.500; 0.650; 0.620; 0.520; 0.510; 0.620; 0.650; 0.500; 0.580; 0.470; 0.650; 0.500; 0.570; 0.550; 0.570; 0.520; 0.540; 0.530; 0.580; 0.580; 0.510; 0.560; 0.450; 0.520; 0.600; 0.530; 0.580; 0.610; 0.540; 0.470; 0.570; 0.430; 0.600; 0.500; 0.510; 0.530; 0.600; 0.490; 0.490; 0.600; 0.500; 0.500; 0.500; 0.500; 0.600; 0.570; 0.580	0.54	All 49 one-miRNA-at-a-time cross-validated AUCs, in source S1 Appendix Table A order; median 0.540. These are standalone diagnostic models, not panel feature-ranking AUCs. Original reported directions retained, including AUCs below 0.5. Missingness varies markedly across markers.
Yang et al. (2024)	Exosomal miR-223	Individual circulating miRNA	0.812; 0.839; 0.778; 0.778	0.795	Eligible diagnostic comparisons reported in the study.
Yoshimura et al. (2018)	Individual circulating miRNAs	Individual circulating miRNA	0.880; 0.850; 0.700	0.85	All eligible single-miRNA diagnostic AUCs.
Zhu et al. (2022)	Exosomal miR-205	Individual circulating miRNA	0.715	0.715	Standalone exosomal miRNA ROC.
Chen et al. (2020)	miR-125b	Individual circulating miRNA	0.730	0.73	Source Figure 2A: serum miR-125b diagnostic AUC 0.730; image independently checked.
Gao & Wu (2015)	miR-200c; miR-141	Individual circulating miRNA	0.790; 0.750	0.77	Standalone serum miRNA ROC estimates.
Guo et al. (2013)	miR-92	Individual circulating miRNA	0.803	0.803	EOC vs healthy controls.
Helmy et al. (2025)	miR-200b	Individual circulating miRNA	0.743	0.743	EOC vs non-malignant cases.
Meng et al. (2016)	Exosomal individual miRNAs	Individual circulating miRNA	0.914; 0.815; 0.655	0.815	Eligible exosomal single-miRNA ROC estimates.
Meng et al. (2015)	miR-7; miR-25; miR-93; miR-429	Individual circulating miRNA	0.675; 0.834; 0.790; 0.845	0.812	Standalone serum single-miRNA ROC estimates.
Minareci et al. (2024)	miR-4314; miR-1181	Individual circulating miRNA	0.780; 0.760; 0.740; 0.700; 0.810	0.76	Source Results: overall miR-4314/miR-1181 AUCs 0.780/0.760; stage I 0.740/0.700; serous-histology miR-4314 0.810, each versus healthy controls. Same-cohort subgroup analyses, not independent

Study	Biomarker/model	Figure 8 category	Eligible raw AUC value(s)	Representative AUC	Selection note
					validation.
Song et al. (2020)	miR-26b; miR-21	Individual circulating miRNA	0.753; 0.826	0.7895	OC versus healthy controls; same-cohort single-marker ROC; source Table III. miR-26b CI 0.708–0.825; miR-21 CI 0.821–0.932.
Zhang et al. (2020)	Exosomal miR-484	Individual circulating miRNA	0.821	0.821	OC vs healthy controls.
Zhao et al. (2018)	miR-106a	Individual circulating miRNA	0.879	0.879	Standalone plasma miRNA ROC.
Zuberi et al. (2016)	miR-199a	Individual circulating miRNA	0.704	0.704	Diagnostic EOC-vs-control AUC only; prognostic AUCs excluded.
Zuberi et al. (2020)	miR-145	Individual circulating miRNA	0.928	0.928	Diagnostic EOC-vs-control AUC only; staging/prognostic AUCs excluded.
Shah et al. (2018)	miR-375; miR-210	Individual circulating miRNA	0.765; 0.751	0.758	Standalone serum single-miRNA diagnostic AUCs.
Kan et al. (2012)	Individual miRNAs	Individual circulating miRNA	0.675; 0.722; 0.727; 0.648; 0.684; 0.702	0.693	Source Table 2: volume + miR-103 normalization, then volume-only normalization; all six eligible standalone AUCs retained.
Wang et al. (2019) - five serum miRNAs	Five individual serum miRNAs	Individual circulating miRNA	0.726; 0.693; 0.698; 0.687; 0.689	0.693	Individual diagnostic AUCs from serum study.
Wang et al. (2019) - plasma-based miRNAs	miR-205-5p; miR-145-5p; miR-10a-5p; miR-346; miR-328-3p	Individual circulating miRNA	0.681; 0.702; 0.680; 0.737; 0.700	0.7	OC versus healthy controls; combined training+validation cohort. Source Supplementary Figure S1 and Results diagnostic-value subsection.
Niemira et al. (2023)	Serum miR-1246; miR-150-5p; miR-142-3p; miR-15a-5p; miR-144-3p; miR-126-3p; miR-15b-5p; miR-191-5p; miR-4516; miR-630; miR-106b-5p	Individual circulating miRNA	0.923; 0.872; 0.833; 0.824; 0.822; 0.810; 0.801; 0.796; 0.784; 0.782; 0.750	0.810	HGSOC versus healthy controls; NanoString discovery ROC analyses. Source Supplementary Table S2 and Figure S6. All 11 separately measured miRNAs included; joint miR-4454/miR-7975 probe AUC 0.856 excluded from the individual-miRNA category.
Zheng et al. (2013)	miR-205; let-7f	Individual circulating miRNA	0.609; 0.780	0.6945	Validation-phase single-marker AUCs.
Liu et al. (2021)	Exosomal miR-4732-5p	Individual circulating miRNA	0.889	0.889	OC versus healthy controls; qPCR validation (28 OC/17 controls); source Figure 4d and Results diagnostic-biomarker subsection.
Ali et al. (2022)	miR-204	Individual circulating miRNA	0.924; 0.942	0.933	Cancer-specific early- and late-stage AUCs; benign-vs-control AUC excluded.
Zuberi et al. (2016) [19]	Serum miR-125b	Individual circulating miRNA	0.728	0.728	EOC versus healthy controls; 95% CI 0.64–0.81. Binary survival AUC 0.660 excluded. Probable cohort overlap with [41,42]; publication counts do not represent independent cohorts.
Meng et al. (2016) [25]	Cell-free miR-373 and miR-200a/b/c; three-miR-200 panel	Individual circulating miRNA	0.853; 0.953; 0.903	0.903	Book-chapter report; possible reuse of the Hamburg cohort in [39,40]. No independent validation; comparator labels differ between figure and narrative. Source values: miR-200a/b/c AUCs 0.853/0.953/0.903; combined panel 0.959 (Figure 1.1).
Tian et al. (2025) [29]	miR-338-3p; CMD model combining it with three circRNAs	Individual circulating miRNA	0.905; 0.600	0.7525	Same-cohort logistic models; no independent validation. CMD is a miRNA–circRNA model, not a multi-miRNA or miRNA–protein model. Source values: miR-338-3p AUC 0.905 overall and 0.600 early-stage; CMD AUC 0.9789 overall and 1.000 early-stage (Figures 8B,C).
Günel et al. (2019) [35]	Circulating hsa-let-7d-3p	Individual circulating miRNA	0.700	0.7	qPCR confirmation does not establish an independent cohort. Source calls the specimen serum but describes EDTA collection; coded plasma consistently with [46]. Possible Istanbul-cohort overlap. Source values: AUC 0.700 (95% CI 0.4595–0.9405); no independently validated threshold performance.

B. Multi-miRNA panel/signature

Study	Biomarker/model	Figure 8 category	Eligible raw AUC value(s)	Representative AUC	Selection note
Choi et al. (2020)	Multi-miRNA model	Multi-miRNA panel/signature	0.904; 0.901	0.9025	Eligible panel AUCs.
Cirillo et al. (2021)	miR-320b + miR-141-3p	Multi-miRNA panel/signature	0.723; 0.789; 0.679; 0.979; 0.961; 0.880; 0.637	0.789	Source Table 4: clinical holdout overall/early/late AUCs 0.723/0.789/0.679; Figure 4: four external serum GEO evaluations 0.979/0.961/0.880/0.637. Clinical holdout is not an externally recruited cohort.
Elias et al. (2017)	Serum miRNA classifier	Multi-miRNA panel/signature	0.850	0.85	Final locked independent external qPCR validation.
Gockley et al. (2022)	Multi-miRNA model	Multi-miRNA panel/signature	0.870	0.87	Validation AUC.
Hamidi et al. (2021)	10-miRNA machine-learning signature	Multi-miRNA panel/signature	1.000; 0.926; 1.000; 1.000; 1.000	1	External validation classifier AUCs; individual ML feature AUCs not entered in single-marker category.
Kan et al. (2012)	Two normalization-specific miR-200-family	Multi-miRNA panel/signature	0.784; 0.705	0.7445	Source Table 2: miR-200b + miR-200c (volume + miR-

Study	Biomarker/model	Figure 8 category	Eligible raw AUC value(s)	Representative AUC	Selection note
	panels				103), 0.784; miR-200a + miR-200b (volume only), 0.705. Both normalization-specific models retained.
Kandimalla et al. (2021)	OCaMIR 8-miRNA signature	Multi-miRNA panel/signature	0.850; 0.860; 0.820; 0.860; 0.920; 0.810	0.855	Independent retrospective/prospective serum validation estimates; training values excluded.
Kumar et al. (2021)	Three-miRNA serum panel	Multi-miRNA panel/signature	0.959; 0.952	0.9555	Detailed Results/figure values; abstract discrepancy not used.
Kumar et al. (2022)	miR-34a/let-7f/miR-31 panel	Multi-miRNA panel/signature	0.955; 0.969	0.962	Advanced- and early-stage panel AUCs.
Niemira et al. (2023)	Serum two-miRNA models	Multi-miRNA panel/signature	1.000; 0.952; 0.946; 0.895	0.949	Source Figure 5 and Supplementary Table S4: internal tests of miR-1246/miR-150-5p (1.000) and miR-1246/miR-144-3p (0.952). Figure 6: refitted qPCR test 0.946; Figure 7A/Table 3: refitted GEO test 0.895. Refits are not unchanged-model external validations.
Oliveira et al. (2019)	Two-miRNA plasma model	Multi-miRNA panel/signature	0.820	0.82	Validation AUC; discovery AUC excluded because validation available.
Su et al. (2019)	miR-1307 + miR-375	Multi-miRNA panel/signature	0.837	0.837	Two-miRNA exosomal panel.
Wang et al. (2019) - plasma-based miRNAs	Five-miRNA plasma panel	Multi-miRNA panel/signature	0.763	0.763	OC versus healthy controls; validation 69/66, source Figure 3B. Training AUC 0.788 (Fig. 3A) excluded.
Wang et al. (2019) - five serum miRNAs	Five-miRNA serum panel	Multi-miRNA panel/signature	0.745	0.745	Validation AUC; training AUC 0.783 excluded from representative calculation.
Wang et al. (2022)	OCEM 8-miRNA EV signature	Multi-miRNA panel/signature	0.9375; 0.9515; 0.905	0.9375	Blood-based validation only; ascites, tissue and urine excluded.
Wiczling et al. (2019)	Combined circulating-miRNA subsets	Multi-miRNA panel/signature	0.650; 0.720	0.685	Cross-validated panel AUCs.
Yokoi et al. (2017)	Eight-miRNA serum panel	Multi-miRNA panel/signature	0.968; 0.902	0.935	Eligible panel AUCs.
Yokoi et al. (2018)	Integrated extracellular miRNA models 1 and 3	Multi-miRNA panel/signature	1.000; 0.860	0.93	Source Figures 1 and 3: validation models 1 and 3 compare OC with non-cancer/benign or borderline comparators. Model 2 AUC 0.870 excludes neither other cancers nor their contribution and is excluded from this OC-versus-noncancer summary.
Gahlawat et al. (2022)	Seven-miRNA panel	Multi-miRNA panel/signature	0.900; 0.780; 0.910	0.9	External validation AUCs prioritized.
Li et al. (2023)	sEVmiR-EOC model	Multi-miRNA panel/signature	0.973; 0.924; 0.903	0.924	Testing, validation, and early-stage post-training estimates.
Meng et al. (2016)	Exosomal multi-miRNA panel	Multi-miRNA panel/signature	0.925	0.925	Combined exosomal panel AUC.
Meng et al. (2015)	Four-miRNA serum classifier	Multi-miRNA panel/signature	0.980	0.98	EOC versus healthy controls; four-marker logistic classifier (miR-7, miR-25, miR-93, miR-429), source Figure 1D/E. Seven markers were assayed. Internal test-set result (Figure 1D labels the test set); cases were split 60:40. Control allocation between splits is not clearly specified.
Minareci et al. (2024)	miR-1181 + miR-4314	Multi-miRNA panel/signature	0.810; 0.720	0.765	Source Results: overall two-miRNA model, 0.810; stage I model, 0.720 (95% CI 0.69–0.77). Same-cohort subgroup analysis.
Ranmale et al. (2025)	Serum miR-182 + miR-106a + miR-23b	Multi-miRNA panel/signature	0.743	0.743	OC versus healthy controls; same-cohort diagnostic ROC analysis of the three-marker combination, not all ten measured miRNAs.
Song et al. (2020)	miR-26b + miR-21	Multi-miRNA panel/signature	0.905	0.905	OC versus healthy controls; same-cohort combined-marker ROC. Source Table III: AUC 0.905 (95% CI 0.817–0.953). Narrative AUC 0.916 conflicts with the table; the tabulated value is used without assuming either is a typographical error.
Zhang & Hu (2022)	Four-miRNA model	Multi-miRNA panel/signature	0.967	0.967	Ovarian-cancer-specific validation AUC.
Zheng et al. (2013)	miR-205 + let-7f panel	Multi-miRNA panel/signature	0.831; 0.895; 0.814; 0.813; 0.783; 0.726	0.8135	Validation-phase I/II overall, early-stage, and low-CA125 comparisons.
Suryawanshi et al. (2013) [22]	Plasma three-miRNA LDA classifiers	Multi-miRNA panel/signature	0.96; 0.92; 0.90; 0.88	0.910	Source Figure 2B,C,E,F; median of cancer-versus-healthy/endometriosis AUCs. Figure 2A (endometriosis vs healthy) and 2D (EAOC vs serous OC) excluded. LOOCV with discovery-sample reuse; not independent validation.
Shah et al. (2018)	Serum miR-375 + miR-210	Multi-miRNA panel/signature	0.946	0.946	Source Table 3: OC versus healthy controls; same-cohort two-miRNA diagnostic model. Surgical-outcome analyses are excluded.
Meng et al. (2016) [25]	Cell-free miR-200a/b/c panel	Multi-miRNA panel/signature	0.959	0.959	Source Figure 1.1. Same-cohort estimate; possible overlap with [39,40]. Comparator labels differ between the figure and narrative.

C. miRNA + CA125 and/or HE4

Study	Biomarker/model	Figure 8 category	Eligible raw AUC value(s)	Representative AUC	Selection note
Cirillo et al. (2021)	miR-320b + miR-141-3p + CA125 + HE4	miRNA + CA125 and/or HE4	0.989; 1.000; 0.983	0.989	Source Table 4: clinical holdout overall, early-stage

Study	Biomarker/model	Figure 8 category	Eligible raw AUC value(s)	Representative AUC	Selection note
					and late-stage miRNA/protein-model AUCs, respectively.
Liang et al. (2020)	miR-183 + CA125/HE4 model	miRNA + CA125 and/or HE4	0.920	0.92	Independent validation AUC; training AUC 0.951 excluded.
Ren et al. (2018)	miR-193a-5p + HE4 + CA125	miRNA + CA125 and/or HE4	0.996	0.996	Combined diagnostic model.
Su et al. (2019)	miRNA(s) + CA125 and/or HE4	miRNA + CA125 and/or HE4	0.959; 0.857; 0.970; 0.940; 0.857; 0.945; 0.874; 0.977	0.9425	All eligible miRNA-containing CA125/HE4 combinations; CA125+HE4 alone excluded.
Yang et al. (2024)	Exosomal miR-223 + CA125	miRNA + CA125 and/or HE4	0.937; 0.952; 0.918; 0.944	0.9405	Eligible diagnostic comparisons.
Zhu et al. (2022)	Exosomal miR-205 + CA125/HE4	miRNA + CA125 and/or HE4	0.930; 0.827; 0.951	0.93	All eligible miRNA-containing combinations.
Chen et al. (2020)	miR-125b + CA125	miRNA + CA125 and/or HE4	0.900	0.9	Source Figure 2C: miR-125b + CA125 diagnostic AUC 0.900; image independently checked.
Gahlawat et al. (2022)	miRNA panel + CA125	miRNA + CA125 and/or HE4	0.970; 0.930; 0.980	0.97	Source Figure 4: overall, early- and late-stage OC versus healthy controls, respectively. CA125 was unavailable in the external cohort; these are development-cohort estimates.
Shah et al. (2018)	miRNA(s) + CA125	miRNA + CA125 and/or HE4	0.956; 0.952; 0.984	0.956	All eligible CA125-containing miRNA models.
Zhang et al. (2020)	Exosomal miR-484 + CA125	miRNA + CA125 and/or HE4	0.912	0.912	Combined diagnostic AUC.
Yokoi et al. (2017)	Six-miRNA panel + CA125	miRNA + CA125 and/or HE4	0.994	0.994	Combined miRNA + CA125 model.
Yoshimura et al. (2018)	miRNA + CA125 combinations	miRNA + CA125 and/or HE4	0.950; 0.910; 0.810	0.91	All eligible miRNA + CA125 diagnostic AUCs.

D. Multimodal model

Study	Biomarker/model	Figure 8 category	Eligible raw AUC value(s)	Representative AUC	Selection note
Oliveira et al. (2019)	Two miRNAs + CA125 + age	Multimodal model	0.960	0.96	Validation model; includes a clinical covariate (age).
Webber et al. (2024)	10-miRNA + protein biomarkers + age + menopausal status	Multimodal model	0.950	0.95	External validation AUC; broader protein/metadata model.
Ali et al. (2022)	miR-204 + CA125 + CA19.9	Multimodal model	1.000; 0.998	0.999	Cancer-specific early- and late-stage AUCs; classified as multimodal because CA19.9 is an additional non-CA125/HE4 biomarker.

Studies reviewed but not contributing an AUC to Figure 8

Study	Reason for exclusion from Figure 8 AUC calculation
Frisk et al. (2025)	No eligible ovarian-cancer diagnostic ROC-AUC for Figure 8.
Langhe et al. (2015)	Differential expression study without eligible diagnostic ROC-AUC.
Benson et al. (2015)	Treatment-monitoring rather than OC-vs-control diagnostic performance.
Penyige et al. (2019)	Exploratory profiling without eligible diagnostic ROC-AUC.
Robelin et al. (2020)	Treatment-response/surgical prediction rather than OC diagnosis.
Yunusova et al. (2023)	No eligible diagnostic ROC-AUC reported.
Pal et al. (2024)	AUCs classify treatment responders vs non-responders, not OC vs controls.
Gong et al. (miR-148a)	Primarily prognostic; no eligible OC-vs-control diagnostic ROC-AUC.
Ritter et al. (2020)	Candidate profiling without eligible diagnostic ROC-AUC.
Paliwal et al. (miR-22/miR-21)	No diagnostic ROC-AUC reported.
Halvorsen et al. (2017)	Prognostic/predictive treatment-response study.
Yoshida et al. (2021)	Prognostic/prediction paper, not OC-vs-control diagnosis.
Fukagawa et al. (2017)	Prognostic/biological biomarker study without eligible diagnostic ROC-AUC.
Chen et al. (2016, miR-183)	Prognostic/functional study without eligible diagnostic ROC-AUC.
Habel et al. (2023)	Diagnostic expression differences but no ROC-AUC.
Kapetanakis et al. (2015)	Treatment/PFS monitoring; no eligible diagnostic ROC-AUC.
Uehara et al. (2024)	AUCs predict residual tumor/treatment selection, not OC diagnosis.
Zhong et al. (2025)	AUCs evaluate chemotherapy prognosis/outcome, not OC diagnosis.
Hong et al. (2013, miR-221)	No ROC-AUC reported for OC-vs-control diagnosis.
Maeda et al. (2020)	Stage/lymph-node/recurrence associations; no OC-vs-control diagnostic ROC-AUC.
Chao et al. (2014)	Postoperative/recurrence monitoring; no eligible diagnostic ROC-AUC.
Stawiski et al. (2024)	AUC distinguishes future OC cases sampled <2 years vs 2-7 years before diagnosis; no non-cancer control comparison.
Xu et al. (2013) [18]	No diagnostic ROC-AUC reported; expression and survival associations retained in qualitative synthesis.
Zhou et al. (2023) [20]	Reported model ROC-AUCs predict surgical residual disease, not OC versus non-cancer controls.
Hong et al. (2022) [54]	The 17-miRNA-pair AUC 0.9205 compares OC with other cancers, not non-cancer controls (source Results, 17-miRP classifier). It is excluded from Figure 8. No AUC was inferred from the 13-pair classifier sensitivity/specificity.
Günel et al. (2018) [21]	AUC 0.70 concerns detection of established recurrence; retained in Table 1, excluded from the primary-diagnosis AUC comparison under the existing endpoint rule.
Shapira et al. (2014) [23]	Reported ROC values discriminate short versus long survival, not cancer versus noncancer; no diagnostic AUC contribution.
Xiong et al. (2021) [26]	Expression comparison only; no diagnostic AUC.
Ruan et al. (2018) [27]	Same-dataset clustering accuracy is not ROC AUC; no diagnostic AUC contribution.
Ji et al. (2014) [28]	Expression comparison only; no diagnostic AUC.
Tian et al. (2025) [29]	CMD miRNA–circRNA model: AUC 0.9789 overall and 1.000 early-stage. It does not fit the four predefined Figure 8 categories; retained here and in Table 1, not forced into a miRNA–protein or multi-miRNA category.
Sun et al. (2024) [30]	Clinical expression/response comparisons without diagnostic AUC.
Timofeeva et al. (2023) [31]	Chemotherapy-response models (source Table 8), not cancer-versus-noncancer diagnosis. Tissue and PGR-phenotype performance excluded from Figure 8.
Timofeeva et al. (2022) [32]	Source Figure 7a surgical-outcome AUCs: 0.77, 0.77, 0.74, 0.73; Figure 7b chemotherapy-response AUCs: 0.91, 0.90, 0.90, 0.90 (models 1–4 respectively). These are clinical-outcome predictions, not primary cancer diagnosis. Tissue-based diagnostic models also excluded.
Gumusoglu et al. (2021) [33]	Expression profiling without diagnostic AUC.
An et al. (2020) [34]	Serum expression comparison without diagnostic AUC.
Kumari et al. (2022) [24]	Source Table IV reports OC miR-145 AUC 0.790 (95% CI 0.653–0.927), sensitivity 90% and specificity 73%. The study contains endometrial cancer, endometriosis and benign-control groups, and describes differentiation from other study groups; a pure OC-versus-noncancer ROC comparator cannot be confirmed. Retained in Table 1 but excluded from Figure 8 under the existing comparator rule.

Wiczling source order for the 49 individual-marker AUCs: hsa-let-7e-3p; hsa-let-7f-1-3p; hsa-let-7i-3p; hsa-miR-101-3p; hsa-miR-128-3p; hsa-miR-130a-3p; hsa-miR-134-5p; hsa-miR-140-3p; hsa-miR-142-5p; hsa-miR-144-5p; hsa-miR-145-5p; hsa-miR-146a-5p; hsa-miR-148a-3p; hsa-miR-154-5p; hsa-miR-15a-5p; hsa-miR-15b-5p; hsa-miR-16-5p; hsa-miR-17-5p; hsa-miR-186-5p; hsa-miR-191-5p; hsa-miR-194-5p; hsa-miR-197-3p; hsa-miR-199a-3p; hsa-miR-219a-5p; hsa-miR-221-3p; hsa-miR-221-5p; hsa-miR-223-5p; hsa-miR-22-3p; hsa-miR-22-5p; hsa-miR-26b-3p; hsa-miR-29c-3p; hsa-miR-29c-5p; hsa-miR-30c-5p; hsa-miR-320b; hsa-miR-324-3p; hsa-miR-33b-5p; hsa-miR-346; hsa-miR-362-5p; hsa-miR-374a-5p; hsa-miR-381-3p; hsa-miR-424-3p; hsa-miR-483-5p; hsa-miR-509-3p; hsa-miR-550a-3p; hsa-miR-604; hsa-miR-629-3p; hsa-miR-664a-3p; hsa-miR-877-3p; hsa-miR-99b-5p.
