# Supplementary material for: Circulating miRNAs and Multivariate Biomarker Signatures in Ovarian Cancer: A Systematic Review
Source: Int J Mol Sci. 2026 Oct 2;27(19):8823. doi: 10.3390/ijms27198823 (PMC13664815; doi:10.3390/ijms27198823)
Supplement: Supplementary file 1 [file ijms-27-08823-s001.zip › Supplementary_Table_S6_Full_Text_Exclusions.pdf]

Supplementary Table S6

Reports excluded after full-text assessment and reports not retrieved

Fifteen reports were excluded after full-text assessment. Each report is identified by its complete article title and Rayyan screening record. One primary exclusion reason is reported per record.

Report (author, year)	Published article title / Rayyan record	Standardized category	Primary reason for exclusion
1. Slack & Weidhaas (2008)	MicroRNA in cancer prognosis. (rayyan-380781316)	Ineligible publication type or companion report	Opinion/editorial; not an eligible primary clinical study.
2. Záveský & Slanár (2023)	Discovery and Evaluation of Extracellular MicroRNA Biomarkers in Plasma, Ascites, and Urine. (rayyan-380781326)	Ineligible publication type or companion report	Methods/protocol chapter; no eligible clinical cohort.
3. Hussain & Khaliq (2025)	Determination of ROC Curve based diagnostic strength of miRNA in ovarian cancer. (rayyan-380781449)	Ineligible biospecimen	miRNAs were measured in white blood cells rather than the circulating cell-free fraction.
4. Alharbi et al. (2020)	miRNa signature in small extracellular vesicles and their association with platinum resistance and cancer recurrence in ovarian cancer. (rayyan-380781484)	Clinical outcome outside the selected scope	Cell-based miRNA analysis without eligible measurements in patient-derived blood samples.
5. Kuhlmann et al. (2014)	Circulating U2 small nuclear RNA fragments as a novel diagnostic tool for patients with epithelial ovarian cancer. (rayyan-380781515)	Non-miRNA biomarker	Assessed U2 small nuclear RNA fragments rather than circulating miRNAs.
6. Tuncer et al. (2020)	miRNA expression profile changes in the peripheral blood of monozygotic discordant twins for epithelial ovarian carcinoma: potential new biomarkers for early diagnosis and prognosis of ovarian carcinoma. (rayyan-380781517)	Ineligible biospecimen	miRNAs measured in isolated peripheral-blood mononuclear cells (lymphocytes), outside the eligible blood-miRNA specimen scope.
7. Hu et al. (2021)	Graphene oxide-based qRT-PCR assay enables the sensitive and specific detection of miRNAs for the screening of ovarian cancer. (rayyan-380781571)	Technical or bioinformatic study	Primarily an assay/technology-development study for miRNA detection or identification, outside the clinical biomarker scope of this review.
8. Yang et al. (2025)	Ultrasensitive detection of miRNA with single-nucleotide resolution using a high-electron-mobility transistor biosensor combined with splint-ligation. (rayyan-380781601)	Technical or bioinformatic study	Primarily an assay/technology-development study for miRNA detection or identification, outside the clinical biomarker scope of this review.
9. Robelin et al. (2019)	Comparison of 11 circulating miRNAs and CA125 kinetics in ovarian cancer during first line treatment: data from the randomized CHIVA trial (a GINECO-GCIG study) (rayyan-443071403)	Ineligible publication type or companion report	Conference/companion report; the full eligible publication was retained.
10. Zhang et al. (2025)	Rapid and Sensitive Detection of miRNA by Single-Molecule Fluorescence Dequenching Assay with Target Recycled CRISPR/Cas12a Amplification System (rayyan-462034909)	Technical or bioinformatic study	Primarily an assay/technology-development study for miRNA detection or identification, outside the clinical biomarker scope of this review.
11. Skryabin et al. (2024)	Integrated miRNA Profiling of Extracellular Vesicles from Uterine Aspirates, Malignant Ascites and Primary-Cultured Ascites Cells for Ovarian Cancer Screening (rayyan-462034974)	Ineligible biospecimen	miRNAs were evaluated in uterine aspirates/ascites rather than an eligible blood-derived specimen.
12. Ge et al. (2022)	Plasma circRNA microarray profiling identifies novel circRNA biomarkers for the diagnosis of ovarian cancer (rayyan-462035052)	Non-miRNA biomarker	Evaluated circular RNA rather than circulating miRNA.
13. Dogan et al. (2022)	Integrated bioinformatics analysis of validated and circulating miRNAs in ovarian cancer (rayyan-462035067)	Technical or bioinformatic study	Computational analysis of miRNA target genes and pathways; no clinical evaluation of patient-level circulating-miRNA measurements.
14. Nowak et al. (2015)	Current clinical application of serum biomarkers to detect ovarian cancer (rayyan-462035241)	Ineligible publication type or companion report	Review article; not an eligible primary study.
15. Ivanov et al. (2023)	"Silicon-On-Insulator"-Based Biosensor for the Detection of MicroRNA Markers of Ovarian Cancer. (rayyan-462035044)	Technical or bioinformatic study	Primarily an assay/technology-development study for miRNA detection or identification, outside the clinical biomarker scope of this review.

Category totals: Ineligible publication type or companion report (n = 4); Ineligible biospecimen (n = 3); Non-miRNA biomarker (n = 2); Technical or bioinformatic study (n = 5); Clinical outcome outside the selected scope (n = 1).

Note: Exclusion reasons were standardized to the categories reported in the PRISMA flow diagram.

Reports not retrieved

Kuhlmann, J.D.; Chebouti, I.; Kimmig, R.; Buderath, P.; Reuter, M.; Puppel, S.-H.; Wimberger, P.; Kasimir-Bauer, S. Extracellular vesicle-associated miRNAs in ovarian cancer – design of an integrated NGS-based workflow for the identification of blood-based biomarkers for platinum-resistance. Clin. Chem. Lab. Med. 2019, 57, 1053–1062. https://doi.org/10.1515/cclm-2018-1048. Rayyan record: rayyan-380781535.

Full text unavailable despite retrieval attempts, including institutional access. Eligibility could not be assessed. This report is counted under “Reports not retrieved” in Figure 1 and is not included in the 15 full-text exclusions above.

Chen, Liang; Wang, Kangyu; Li, Lei; Zheng, Baibing; Zhang, Qianru; Zhang, Fang; Chen, Jinlong; Wang, Shiwen. Plasma exosomal miR-1260a, miR-7977 and miR-192-5p as diagnostic biomarkers in epithelial ovarian cancer. Future Oncology 2022, 18, 2919–2931. https://doi.org/10.2217/fon-2022-0321. Rayyan record: rayyan-380781338.

Full text could not be retrieved. Eligibility could not be assessed. Together with Kuhlmann et al., this report contributes to the two reports not retrieved in Figure 1; neither is counted among the 15 full-text exclusions.
